# International Network of Antibiotic Allergy Nations (iNAAN): Protocol for a type 2 hybrid effectiveness-implementation multicentre prospective cohort and target trial emulation study evaluating penicillin allergy delabeling via direct oral challenge

**DOI:** 10.1371/journal.pone.0330724

**Published:** 2025-09-05

**Authors:** Elise A. Mitri, Luke R. Fletcher, Camille Paynter, Sara Vogrin, Fiona James, Irvin Ng, Natasha E. Holmes, Courtney Ierano, Jason A. Roberts, Suran L. Fernando, Marlena Klaic, Jason A. Trubiano

**Affiliations:** 1 Department of Infectious Diseases, at the Peter Doherty Institute for Infection and Immunology, Melbourne Medical School, University of Melbourne, Melbourne, Victoria, Australia; 2 Centre for Antibiotic Allergy and Research, Department of Infectious Diseases and Immunology, Austin Health, Melbourne, Victoria, Australia; 3 National Allergy Centre of Excellence (NACE), Parkville, Victoria, Australia; 4 Department of Anaesthesia, Austin Health, Melbourne, Victoria, Australia; 5 Department of Critical Care, University of Melbourne, Melbourne, Victoria, Australia; 6 Data Analytics Research and Evaluation (DARE) Centre, Austin Health, Melbourne, Victoria, Australia; 7 School of Health Sciences, The University of Melbourne, Melbourne, Victoria, Australia; 8 National Centre for Antimicrobial Stewardship, Department of Infectious Diseases, University of Melbourne, Melbourne, Victoria, Australia; 9 University of Queensland Centre for Clinical Research, Faculty of Medicine, The University of Queensland, Brisbane, Queensland, Australia; 10 Herston Infectious Diseases Institute (HeIDI), Metro North Health, Brisbane, Queensland, Australia; 11 Departments of Pharmacy and Intensive Care Medicine, Royal Brisbane and Women’s Hospital, Brisbane, Queensland, Australia; 12 UR UM 103, University of Montpellier, Division of Anesthesia Critical Care and Emergency and Pain Medicine, Nimes University Hospital, Nimes, France; 13 Department of Clinical Immunology and Allergy, Royal North Shore Hospital, St Leonards, New South Wales, Australia; 14 Faculty of Medicine and Health, University of Sydney, Camperdown, New South Wales, Australia; The University of Lahore, PAKISTAN

## Abstract

**Background:**

Penicillin allergies are reported in 1 in 10 hospitalised patients globally and are associated with inferior patient and health service outcomes. However, more than 95% of low-risk penicillin allergies can be removed by direct oral challenge (DOC).

**Objective:**

The International Network of Antibiotic Allergy Nations (iNAAN) aims to evaluate the utility of an audit and feedback (A&F) and education implementation strategy to increase the adoption of penicillin DOC in patients with a low-risk penicillin allergy, while concurrently assessing the impact of penicillin DOC on antibiotic prescribing and health service outcomes.

**Methods:**

This is an international, multicentre type 2 hybrid effectiveness-implementation trial evaluating the widespread safety, clinical effectiveness and implementation of penicillin allergy delabeling via DOC, following penicillin allergy assessment using a purpose-built digital penicillin allergy toolkit within a smartphone application. The implementation strategy encompasses evaluation of co-designed digitally delivered bimonthly A&F reports to health services and clinicians, beginning a minimum of three months post site-activation, and optional education sessions that are delivered by the study team. The primary effectiveness outcome is the proportion of patients with a low-risk penicillin allergy that are delabeled following DOC. The primary implementation outcome is clinician adoption of the digital penicillin allergy toolkit within 6-months of site activation. Participants include adult patients who report a penicillin allergy and have undertaken a formalised penicillin allergy assessment, with or without DOC, at a participating health service. A target trial emulation approach will be used to analyse secondary effectiveness outcomes, including the impact of penicillin DOC on international antibiotic prescribing, infection-related and health service outcomes.

**Discussion:**

This study protocol presents a type 2 hybrid effectiveness-implementation trial design that will provide high level evidence to aid widespread implementation of penicillin DOC.

**Trial registration:**

ACTRN12623000484640 (https://www.anzctr.org.au/) (https://www.anzctr.org.au/Trial/Registration/TrialReview.aspx?id=385815&isReview=true).

## Introduction

Patient-reported antibiotic allergy labels (AALs) are a significant public health concern with an estimated 18% of all hospitalised Australians reporting an antibiotic allergy [1]. This prevalence is higher amongst vulnerable populations, including individuals with chronic illnesses, cancer, and those who are immunocompromised [2]. Penicillin allergy labels are reported in 9.4% of the global population [3] and negatively impact patient outcomes by increasing the risk of inappropriate antibiotic prescribing, adverse events and acquisition of multidrug resistant organisms [4–7]. At a health service level, AALs are associated with increased hospital length-of-stay (LOS), higher readmission rates, increased hospital costs and increased mortality [4,8,9]. At a public health and population level, AALs are associated with inappropriate antibiotic prescribing and antimicrobial resistance [1,10].

Patient reported penicillin allergies are considered ‘low-risk’ in 50% of patients [11]. Assessment and identification of low-risk penicillin allergies may be undertaken using validated assessment tools, including the Antibiotic Allergy Assessment Tool [12] or clinical decision rules, such as PEN-FAST [13]. Low-risk penicillin allergies are amenable to testing via a direct oral challenge (DOC) (i.e., test dose), without prior skin-testing in up to 98% of patients, as demonstrated in the PALACE international, multicentre randomised clinical trial [14]. Penicillin allergy assessment and DOC for identified low-risk allergies has been successfully deployed in single-centre whole-of-hospital programs but widespread international geographical implementation has been limited [11,15–17].

Antimicrobial stewardship smartphone applications have previously demonstrated improved knowledge of antimicrobial prescribing and increased adherence to prescribing guidelines within hospitals [18]. In the PREPARE randomised controlled trial (RCT), a smartphone app with digital adaptation of validated penicillin allergy assessment tools [19,20] was safely piloted by non-allergists [21]. However, whist there is increasing awareness of penicillin allergy assessment and delabeling via DOC [22], and with the intervention deemed safe when deployed by non-allergists [23], there remains heterogeneity in the approach to assessment and delivery of penicillin DOC internationally, a paucity of data in various hospital settings, geographical regions and populations, and an absence of an implementation evaluation of this clinically effective antimicrobial stewardship (AMS) intervention [24].

In evaluating the widespread implementation of low-risk penicillin allergy delabeling via DOC, the International Network of Antibiotic Allergy Nations (iNAAN) study has two aims:

Aim 1 – Evaluation of clinical effectiveness: Utilising a validated penicillin allergy assessment tool and clinical decision rule (i.e.,: digital penicillin allergy toolkit, embedded within a smartphone app), we aim to audit the safety and subsequent antimicrobial prescribing impacts of multidisciplinary led penicillin allergy assessment and delabeling via DOC.
Aim 2 – Evaluation of an implementation strategy: Using a digital audit and feedback (A&F) implementation strategy, as previously demonstrated to be effective in antimicrobial stewardship (AMS) programs [25] and on antibiotic prescribing in primary care [26], we aim to assess the impact of A&F and optional education sessions on adoption of penicillin DOC in health services.

Using a large, international, multicentre prospective observational cohort design, the iNAAN study seeks to answer the remaining clinical effectiveness questions surrounding penicillin allergy assessment and DOC in low-risk phenotypes, which are not amenable to RCT evaluation. Utilising an explicit target trial emulation framework to estimate causal inference [27], we seek to evaluate the clinical effectiveness of penicillin DOC on international antibiotic prescribing, health service and infection-related outcomes.

## Materials and methods

This study was approved on 13^th^ November 2021 by the Austin Health Human Research Ethics Committee (HREC78719/Austin-2021) with ethics approval secured for participating Australian health services via the National Mutual Acceptance Scheme. Participating international health services will apply and receive ethics approval via their local institutional review boards. All ethical information complies with international guidelines. This study was registered on the Australian New Zealand Clinical Trials Registry (ANZCTR) on 12^th^ May 2023 (ACTRN12623000484640), prior to rapid Australian and international interest and uptake across health services and before deployment of the implementation strategy.

**Fig 1:**
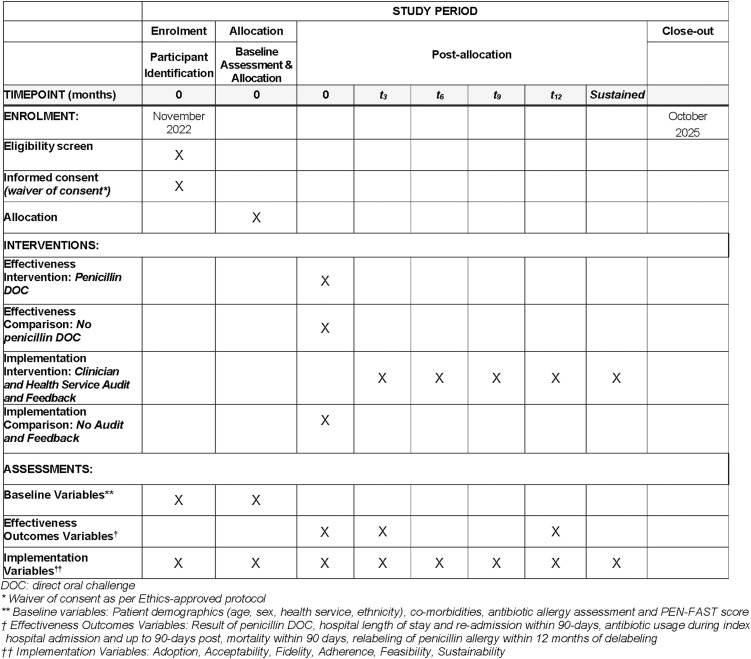
Schedule of enrolment, intervention and assessments.

The first participant was recruited on 9^th^ November 2022 and participant recruitment is ongoing. Participant recruitment for the first iNAAN analysis is expected to be completed by 15^th^ October 2025, after which results will be published. The schedule of enrolment, intervention and assessment is described in Fig 1.

Refer to S1 Checklist for the Standard Protocol Items Recommendations for Interventional Trials (SPIRIT 2025) Checklist used for the study protocol reporting [28].

### Study design

iNAAN is an international multicentre, prospective cohort type 2 hybrid effectiveness-implementation study [29] utilising an A&F and education implementation strategy. Type 2 hybrid implementation trials simultaneously evaluate implementation of a clinical intervention and the effect of the intervention on clinical outcomes. Trial oversight, coordination and statistical analysis will be conducted at Austin Health, Victoria, Australia.

### Study population and setting

iNAAN will prospectively examine penicillin allergy delabeling in adult patients. Participating iNAAN countries, with their respective start date of recruitment, are from; Australia (9^th^ November 2022), South Africa (25^th^ June 2023), Malaysia (15^th^ April 2024), the United Kingdom (16^th^ May 2024), Hong Kong (20^th^ June 2024), Canada (27^th^ June 2024), the United States of America (4^th^ September 2024) and New Zealand (20^th^ December 2024). Participating iNAAN health services include large metropolitan and smaller regional health services, and the relevant Institutional Review Boards are described in S1 Table. Ethnicity and race data of participants will be collected, in accordance with the pre-specified ethnicity and race sub-categories for the country of recruitment (S2 Table).

### Eligibility criteria and patient identification

We will include patients aged ≥ 18 years who report a penicillin allergy and undergo a penicillin allergy assessment + /- delabeling via DOC at a participating health service. Penicillin allergy is defined as a self-reported or documented allergy to any of the following drugs: ‘penicillin unspecified’, phenoxymethylpenicillin, benzylpenicillin, amoxicillin, amoxicillin-clavulanate, ampicillin, flucloxacillin, dicloxacillin, cloxacillin, oxacillin, nafcillin and piperacillin-tazobactam. Patients will be excluded if they are aged < 18 years, or when a penicillin allergy assessment cannot be performed, or a history cannot be obtained. Eligible patients will be identified and assessed by study investigators or their delegates and may include multidisciplinary clinicians such as allergists/immunologists, infectious diseases and antimicrobial stewardship physicians, other specialised medical practitioners, pharmacists, and nurses or nurse practitioners.

### Consent

An ethics-approved waiver of consent was sought for the collection of clinical data pertaining to the penicillin allergy assessment + /- outcomes of delabeling via DOC when performed as part of routine clinical practice at the participating health services. Where an individual health service does not approve a waiver of consent due to local legislative requirements, a Participant Information Consent Form will be utilised to obtain patient consent to collect this same data. Written informed consent will be sought when undertaking the penicillin DOC, as is required as part of routine care by local health services.

### Interventions

#### Effectiveness.

Patients identified as having a low-risk penicillin allergy phenotype utilising the digital penicillin allergy toolkit (‘NAAN App’) and who are deemed appropriate to undertake testing, as determined by the local clinical delabeling protocol and agreed to by the clinician and patient, will be offered a penicillin DOC. Patients who have a non-immune mediated penicillin allergy history may be offered direct removal of the allergy label (‘direct delabel’) or DOC if preferred by the patient. The penicillin DOC procedure will be performed in accordance with the local health service approved delabeling protocol, including patient consent and choice of drug, dose, duration and observation period of the DOC procedure. The result of the DOC, including verification of immune-mediated and non-immune mediated adverse events, will be recorded by the local clinician in the patient medical record and study database.

#### Implementation.

At a minimum of three months following site activation and administration of the digital penicillin allergy toolkit, all iNAAN clinicians and health services will receive bimonthly digitally delivered A&F (‘Health Service Report’) detailing local effectiveness and implementation outcomes of penicillin allergy assessment and delabeling. Additional optional education sessions will be facilitated by the study team and will be open to all investigators and stakeholders at participating health services throughout the study period.

### Comparison

#### Effectiveness.

Patients identified as having a high-risk penicillin allergy phenotype and/or are deemed inappropriate to undertake a DOC, as determined by the clinician, patient and local delabeling protocol, retain their penicillin allergy label and only penicillin allergy assessment data will be submitted to the database.

#### Implementation.

Following health service activation, no A&F will be delivered to the clinicians or health service for a period of at least three months. This will enable analysis of a baseline period of no implementation strategy, compared with the implementation strategy initiated after at least three months for all sites.

### Data collection, management and storage

#### Data sources.

Data sources include patient-provided data at the time of penicillin allergy assessment + /- DOC, and health service medical records to determine antibiotic prescribing pre- and post- evaluation, individual patient infection-related outcomes, and health service outcomes.

#### Minimum data (all sites).

The minimum data for enrolment in the iNAAN study includes; (i) patient demographics (age, sex, health service and ethnicity), (ii) an antibiotic allergy assessment using a standardised format [19] and PEN-FAST score [20] and, (iii) the outcomes of penicillin DOC, if performed.

Where available, additional data may be submitted to the iNAAN database including; (i) patient co-morbidities, (ii) details of hospital admission (length of stay, diagnosis of infection, hospital re-admission within 90-days), (iii) antibiotic usage pre- and up to 90 days post- penicillin allergy evaluation (drug, dose, route, frequency, indication) and relevant microbiological data, and (iv) adverse events related to penicillin DOC (phenotype, grading, severity and outcomes), mortality within 90-days of evaluation and relabeling of penicillin allergy within 12 months of delabeling.

Patients will be identified by either their unique hospital record number or a unique study identifier, depending on the preferences and legislative requirements of the local health service.

#### Data collection – NAAN app.

**Fig 2:**
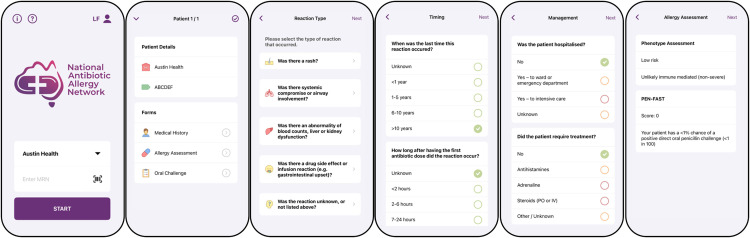
National Antibiotic Allergy Network (NAAN) smartphone App.

Study investigators and clinician delegates utilise the ‘NAAN App’ – a purpose-built digital penicillin allergy toolkit within a smartphone application built for the iOS platform (Apple Inc, CA) – to perform point-of-care patient registration and a standardised, validated penicillin allergy assessment that produces a resultant risk assessment (Antibiotic Allergy Assessment Tool [AAAT]) [19], PEN-FAST score [20] and risk prediction if the patient was to undertake penicillin DOC (Figs 2 and S1). The NAAN App has been rigorously beta-tested against both the AAAT and expert opinion. Re-identifiable data, including a unique patient identifier, patient demographics, the penicillin allergy assessment, medical co-morbidities and the outcomes of penicillin DOC (if performed), are securely transmitted from the NAAN App to the iNAAN database. Long term participant follow-up data may be entered directly into the iNAAN database, without use of the NAAN App. Automated emails are generated from the (Research Electronic Data Capture) REDCap database to remind study investigators to complete individual participant follow-up at 90-days post penicillin allergy evaluation.

Example of penicillin allergy assessment performed using the purpose-built digital penicillin allergy toolkit (NAAN App) with in-built antibiotic allergy assessment tool [19] and PEN-FAST clinical decision rule [20] producing a phenotypic assessment and PEN-FAST score

The NAAN App is not publicly available on any App Store, is available to download only via invitation, and access is protected with individual username and password authentication. At the point of site activation, clinicians are provided with access to the NAAN App and attend an education session on performing penicillin allergy assessment and data entry via the NAAN App and iNAAN REDCap database. Clinicians can only utilise the NAAN App and upload data for the health service in which they are registered. The NAAN App is ‘write-only’ for the purposes of data transmission to the iNAAN database and once submitted, no data remains on the user’s device. The NAAN App does not provide advice regarding specific allergy testing and does not provide instruction on how to perform allergy testing. The decision to perform penicillin DOC remains with the local clinician in accordance with the health service’s clinical protocol.

#### Data management – iNAAN REDCap database.

Study data will be collected by study investigators or their delegates at all health services and transferred from the NAAN App to a single, secure ‘iNAAN database’. This database utilises the REDCap platform (Vanderbilt University, TN) [30] and is hosted by BioGrid Australia, providers of a national data sharing platform that supports local and international health and biomedical research (https://www.biogrid.org.au). Sensitive patient data is handled and processed in accordance with the regulations of each participating country and adhering to ISO27001 standards. Study records will be kept within the password protected REDCap database on password protected computers.

Access to the iNAAN database is restricted to approved study investigators, requiring a secure username and password, and two-factor authentication. The iNAAN project team can view de-identified study data only. Individual health service iNAAN study investigators may access their own site data in a re-identifiable format, for the purposes of completing patient follow-up. The iNAAN REDCap database will be routinely backed up and encrypted on a secure server in accordance with ISO27001 standards. The iNAAN study is anticipated to be ongoing and data will be retained indefinitely.

Approval for access to both the NAAN App and iNAAN REDCap database is administered and managed via an individual site delegation of duties log.

### Outcomes

Given the described Type 2 hybrid implementation trial design, there are two co-primary outcomes: ‘effectiveness’ and ‘implementation’.

#### Primary outcomes.

a) Effectiveness: the primary effectiveness outcome is the proportion of patients with a low-risk penicillin allergy (via NAAN App and/or clinician-determined) that are delabeled following DOC. This is calculated as the number of patients delabeled via DOC divided by the number of patients who undergo DOC. Delabeled is defined as the removal of the penicillin allergy label following a negative DOC result, as determined by the clinician.
b) Implementation: the primary implementation outcome is clinician adoption of the NAAN App, measured by the change in number of active NAAN App users from baseline to 6-months post-site activation.

#### Secondary outcomes.

a) Effectiveness: the secondary effectiveness outcomes include antibiotic prescribing and health service outcomes and are described in Table 1.
b) Implementation: the secondary implementation outcomes include Acceptability, Fidelity, Adherence, Feasibility and Sustainability and are described in Table 1.

**Table 1:** Primary and secondary effectiveness and implementation outcomes.

EFFECTIVENESS
**Primary:** Proportion of patients with a low-risk penicillin allergy that are delabeled following DOC
**Secondary:** **Antimicrobial stewardship (AMS) outcome measures** *Penicillin use* • Proportion of patients that utilise a penicillin pre-testing (at index admission, where relevant) versus post-testing (up to 90 days post-evaluation) • Proportion of patients that utilise a penicillin within 90 days of penicillin allergy assessment in patients who undergo testing compared with patients that do not undergo testing *Narrow spectrum beta-lactam use* • Proportion of patients that utilise a narrow-spectrum beta-lactam^*^ pre-testing (at index admission, where relevant) versus post-testing (up to 90 days post-evaluation) • Proportion of patients that utilise a narrow-spectrum beta-lactam^*^ within 90 days of penicillin allergy assessment in patients who undergo testing compared with patients that do not undergo testing *Restricted antibiotic use* • Proportion of patients that utilise a pre-defined restricted antibiotic^**^ pre-testing (at index admission, where relevant) versus post-testing (up to 90 days post-evaluation) • Proportion of patients that utilise a pre-defined restricted^**^ antibiotic within 90 days of penicillin allergy assessment in patients who undergo testing compared with patients that do not undergo testing • Proportion of patients that utilise a highest priority critically important antimicrobial (HPCIA) as per published definitions^***^ pre-testing (at index admission, where relevant) versus post-testing (up to 90 days post-evaluation) • Proportion of patients that utilise a HPCIA within 90 days of penicillin allergy assessment in patients who undergo testing compared with patients that do not undergo testing • Proportion of patients that utilise a World Health Organisation (WHO) AWaRe ‘Watch’ or ‘Reserve’ antibiotic^****^ as per published definitions pre-testing (at index admission, where relevant) versus post-testing (up to 90 days post-evaluation) • Proportion of patients that utilise a WHO AWaRe ‘Watch’ or ‘Reserve’ antibiotic^****^ within 90 days of penicillin allergy assessment in patients who undergo testing compared with patients that do not undergo testing **Health service outcome measures** • If evaluated as an inpatient, median hospital LOS (days, IQR) for inpatients with a low-risk penicillin allergy that were delabeled versus inpatients with a penicillin allergy that are not delabeled **Economic outcome measures** • Cost-effectiveness of the program model and implementation strategy
**IMPLEMENTATION**
**Primary:** Clinician adoption of the NAAN App, measured by the change in number of NAAN App users from baseline to 6 months post site-activation.
**Secondary:** *Acceptability* • Perception amongst participating hospital clinicians that the penicillin allergy toolkit is agreeable • Measure: Theoretical Framework of Acceptability – adapted for NAAN App *Fidelity* • Proportion of penicillin DOC procedures (intervention) that are adherent to the audited health service local delabeling protocol, including challenge drug, dose and duration. • Data source: Quantitative data from the iNAAN database *Adherence* • Proportion of assessed patients with medical records meeting the Australian National Safety and Quality Health Service Standards (NSQHS) for documentation of antibiotic allergies in the medical record, including accurate documentation of implicated drug, date of reaction, nature of reaction and severity of reaction [31]^†^ • Data source: Quantitative data from the iNAAN database *Feasibility* • Extent to which the digital penicillin allergy toolkit (NAAN App) can be used within a given context. • Measure: Feasibility of Intervention Measure – adapted for NAAN App *Sustainability* • Extent to which the digital penicillin allergy toolkit (NAAN) can be scaled and spread within and across health services. Number of digital penicillin allergy toolkit (NAAN App) users to enter at least one penicillin allergy assessment over a period of baseline to 6 months, then to 12 months post site-activation, incorporating attrition. • Data source: Quantitative data from the iNAAN database – baseline compared with 6 months and then 12 months post activation *Other* • Develop recommendations for national scaling and implementation of the program across the health system, based on feasibility, costs, barriers and facilitators • To determine the changes in health policy

DOC: direct oral challenge.

*Narrow-spectrum beta-lactams: phenoxymethylpenicillin, benzylpenicillin, flucloxacillin, dicloxacillin, ampicillin or amoxicillin.

**Restricted antibiotics: 3^rd^ generation or later cephalosporins, fluoroquinolones, glycopeptides, lincosamides, piperacillin-tazobactam and carbapenems.

***Highest priority critically important antimicrobial (HPCIA): 3^rd^ and 4^th^ generation cephalosporins, fluoroquinolones, polymixins, phosphonic acid derivatives (WHO List of Medical Important Antimicrobials: a risk management tool for mitigating antimicrobial resistance due to non-human use published by the World Health Organization, 2024. Available from: https://cdn.who.int/media/docs/default-source/gcp/who-mia-list-2024-lv.pdf).

****World Health Organization AWaRe ‘Watch’ or ‘Reserve’ antibiotic: Per defined list in WHO Access, Watch, Reserve (AWaRe) classification of antibiotics for evaluation and monitoring of use, published by the World Health Organization, 2021. Available from: https://iris.who.int/handle/10665/345555.

†Australian health services only.

### Adverse events

A positive DOC is defined as any immediate or delayed immune-mediated reaction occurring following penicillin DOC within a timeframe deemed reasonable by the local clinician. Positive DOC events are managed clinically by the local clinicians and sites, including monitoring, treatment, follow-up, relabeling and referral for future allergy testing, where necessary. All reported positive DOC cases will be reviewed and adjudicated by two independent clinicians with antibiotic allergy expertise to categorise the likelihood of immune-mediated vs non-immune mediated events. Serious adverse events are defined as those leading to death, a life-threatening reaction, intensive care unit admission or hospital readmission, persistent or significant disability/incapacity, or an event that requires intervention to prevent permanent impairment or damage, as adjudicated by the local clinician [32]. Following a negative DOC and delabeling, subsequent future re-reactions to penicillin, i.e.,: ‘denovo’ events, will be managed as a new penicillin reaction. This includes relabeling of the allergy in the medical record, documentation in the iNAAN study database and discussion between the local clinician and patient regarding consideration of future penicillin allergy testing.

### Implementation strategy

The iNAAN study utilises the Framework of Implementability [33] to explore acceptability, fidelity and feasibility, subsequently informing sustainability and scalability of a digital penicillin allergy toolkit to increase adoption of DOC as a low-risk penicillin allergy delabeling intervention. The iNAAN implementation strategy is derived from literature describing the delivery of an audit and feedback intervention and an effectiveness-implementation hybrid design [29].

**Fig 3:**
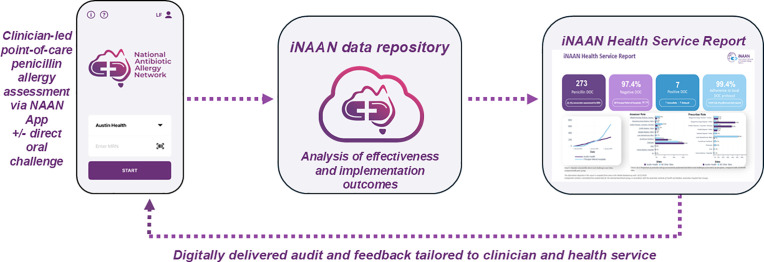
iNAAN data flow.

The implementation strategy consists of two interventions as described in the iNAAN Implementation Manual (S1 Manual). The primary implementation intervention is provision of bi-monthly digital A&F (Health Service Report) containing aggregate, de-identified data delivered to clinicians and health services (S2 Fig). The report is derived from data submitted to the iNAAN database and BioGrid reporting platform, and encompasses local clinical effectiveness outcomes (number of penicillin DOC, negative penicillin DOC rate (%), and number of positive DOC) and implementation outcomes (adoption, fidelity, adherence and sustainability), with hospital peer group benchmarking [34] (Fig 3). Additionally, optional education sessions conducted virtually by the iNAAN study team are open to all activated health services. Attendance data will be used to compare implementation outcomes and provide insights on the influence of education on adoption.

Penicillin allergy assessment + /- delabeling data is captured at point-of-care via the NAAN App and securely transmitted to the iNAAN REDCap database. Bimonthly iNAAN health service reports are provided to clinicians and health services and contain de-identified aggregate data to enable evaluation of the local clinical effectiveness and implementation outcomes of penicillin allergy delabeling via DOC.

#### Data collection – implementation.

Adoption of the NAAN App will be determined using categorical data, disaggregated by discipline, hospital peer group [34] and geographical location, including consideration of urban, regional and remote health services, and by country. Acceptability of the NAAN App and the Health Service Report and will be determined through cross-sectional surveys utilising the Theoretical Framework of Acceptability [35]. Additionally, further evaluation of the iNAAN Health Service Report will be undertaken with end-users via semi-structured focus groups, utilising the Theoretical Domains Framework [36], to ascertain feedback on the content, design and delivery of the Health Service Report, taking into account contextual influences (S1 Manual). Fidelity of DOC procedures, or compliance to the local site penicillin DOC protocol, is measured using quantitative data from the iNAAN database, matching penicillin DOC activity with the health service’s pre-defined protocol, including choice of challenge drug, dose and duration. Adherence of penicillin allergy documentation in the patient medical record, including the drug, date, nature and severity of the reaction, in accordance with the Australian National Safety and Quality Health Service (NSQHS) standards [31], is measured for Australian health services only, both pre- and post- penicillin allergy assessment, utilising quantitative data from the iNAAN database. Feasibility of the NAAN App is evaluated via cross-sectional survey utilising the Feasibility of Intervention Measure [37]. Sustainability will be measured using categorical data from the iNAAN database, evaluating disaggregated data (by discipline, hospital peer group and geographical location), at baseline and at 12-months post activation, to evaluate whether the intervention has evolved into routine practice.

### Sample size

Due to the non-randomised trial design and the defined primary effectiveness and implementation outcomes, a formal sample size calculation is not required. The estimated number of patients undergoing penicillin DOC across all iNAAN health services is 400 per year, however case capture is competitive and will not be capped at this number. This estimate is based on a previous study which performed more than 400 penicillin DOC at two health services over a 2-year period [38].

### Statistical methods

#### Effectiveness evaluation including target trial emulation (TTE).

An analysis of the effectiveness outcomes relating to (i) delabeling, including safety of penicillin DOC led by multidisciplinary clinicians, (ii) antibiotic utilisation pre- and post- penicillin allergy assessment + /- DOC and (iii) economic costs will be performed.

Baseline characteristics will be described using median with interquartile range and frequency with percentage. The primary effectiveness outcome will be presented as count and percentage of all participants who underwent penicillin DOC with 95% confidence intervals.

In participants with complete data, a target trial emulation causative analysis will be performed to evaluate the impact of penicillin DOC on antibiotic prescribing in patients with a pre-defined low-risk penicillin allergy. The target trial specification and emulation, including inclusion and exclusion criteria, intervention (DOC) and control (assessment only) groups, and estimand of interest, was designed prior to study completion (S3 Table). The statistical analysis will mimic per-protocol analysis of target trial using inverse probability of treatment weighting approach. Baseline confounders will be selected with the help of direct acylic graphs and balancing approach (entropy balancing) will be used to generate the weights [39,40]. Generalised linear model with binomial family, log link and robust variance estimator will be used to assess the antibiotic utilisation between those delabeled via DOC and those not delabeled. Results will be expressed as risk ratios with 95% confidence intervals. This analysis will be performed separately for each antibiotic group. Antibiotic utilisation will be defined as the number of participants receiving at least one dose of antibiotic of interest.

A future health economic analysis will be performed outside of the primary publication.

#### Implementation evaluation.

The implementation strategy, as outlined in the iNAAN Implementation Manual (S1 Manual), will use a mixed methods evaluation, balancing in-depth qualitative data with quantitative data from participating sites. Qualitative data, obtained via focus groups utilising semi-structured interview questions, will be thematically analysed to identify barriers and facilitators to behaviour change throughout the audit and feedback implementation strategy. This analysis will be examined using an appropriate theoretical framework, e.g.,: Theoretical Framework of Acceptability [35], Theoretical Domains Framework [36] and the Framework of Implementability [33], to refine the intervention and/or strategy. Constructs from the Consolidated Framework for Implementation Research, such as staffing profile of sites, will be used to explore contextual influences [41].

The effect of the implementation strategy on each of the implementation outcomes, collected on a bimonthly or six-monthly basis, will be evaluated using interrupted time series (ordinary least square methods). This method enables comparison to the time period without the implementation strategy as well as comparison over time, while taking into account the autocorrelation and seasonal effects.

### Inclusivity in global research

Additional information regarding the ethical, cultural, and scientific considerations specific to inclusivity in global research is included in the Supporting Information (S2 Checklist).

### Oversight and monitoring

The iNAAN study governance is overseen by a 10-member international steering committee, with multidisciplinary representation from infectious diseases, allergy/immunology, nursing, pharmacy, international investigators and consumers. The steering committee will provide practical and strategic oversight including proposed study protocol modifications, review study progress, assess requests for study or database expansion, and review proposals for presentation or publication of study results utilising iNAAN data.

### Study progress and dissemination

Following local governance approval, activation of participating iNAAN health services is ongoing. To adequately evaluate both primary effectiveness and implementation outcomes, an initial publication of data is planned after recruitment of a minimum of 1000 penicillin DOC procedures and when more than 85% of activated health services have received at least three episodes of A&F, which is expected to occur in October 2025. The initial publication will include analyses of patient demographics, penicillin allergy assessment and phenotype, outcomes of penicillin DOC and the impact of penicillin DOC on antibiotic prescribing, and will be published and presented in journals and scientific forums relevant to Allergy/Immunology and Infectious Diseases and Antimicrobial Stewardship. The iNAAN study is progressing according to the planned timeline and is anticipated to run ongoing for a minimum of three years from first site activation. Further results, including evaluation of defined secondary effectiveness and implementation outcomes, are planned to be published and/or presented in a variety of scientific forums and journals.

Principal investigators from each participating site will be included as authors. Authorship will be guided by the iNAAN steering committee with reference to the International Committee of Medical Journal Editors guidelines.

## Discussion

This study protocol describes a prospective, international, multicentre type 2 hybrid effectiveness-implementation trial design that will examine the effectiveness and safety of penicillin DOC for low-risk penicillin allergy in adults, and an evaluation of an A&F and education implementation strategy. Whilst the current implementation of the digital penicillin allergy toolkit is limited by use in iOS devices only, future results examining the safety and effectiveness of penicillin allergy evaluation via the NAAN App, and the acceptability of a single operating system, will inform scope to expand to other operating systems. Additionally, while this study is limited to adult patients, future scope exists to adapt the iNAAN platform for evaluation of penicillin DOC in the paediatric population.

The iNAAN study is anticipated to be the largest prospective adult penicillin allergy study since Gadde *et al* [42], with a hybrid implementation trial design that addresses the effectiveness of penicillin DOC on international antibiotic prescribing and examines an implementation strategy to support adoption and sustainability of a key AMS intervention. The iNAAN study will deliver high-level evidence to assist global widespread adoption and implementation of penicillin DOC led by multidisciplinary clinicians, improving equity of access to antibiotic allergy delabeling pathways, particularly in areas where Allergy and Immunology services are limited.

## Supporting information

S1 Checklist Standard Protocol Items Recommendations for Interventional Trials (SPIRIT 2025). (DOCX)

S1 Table Participating iNAAN health services. (DOCX)

S2 Table Ethnicity and race sub-categories for participating iNAAN countries. (DOCX)

S1 Fig NAAN smartphone application risk assessment algorithm. (PDF)

S1 Manual iNAAN implementation manual. (DOCX)

S2 Fig iNAAN health service report example. (DOCX)

S3 Table iNAAN target trial specification and emulation using observational data. (DOCX)

S2 Checklist Inclusivity in global research. (DOCX)

S1 File iNAAN ethics protocol. (DOCX)
