# Supplementary material for: International Network of Antibiotic Allergy Nations (iNAAN): Protocol for a type 2 hybrid effectiveness-implementation multicentre prospective cohort and target trial emulation study evaluating penicillin allergy delabeling via direct oral challenge
Source: PLoS One. 2025 Sep 5;20(9):e0330724. doi: 10.1371/journal.pone.0330724 (PMC12412947; doi:10.1371/journal.pone.0330724)
Supplement: S1 Table — (DOCX) [file pone.0330724.s002.docx]

**S1 Table. Participating iNAAN health services**

| **Health Service, Location** | **Ethics / Institutional Review Board** | **Australian Institute of Health and Welfare Hospital Peer Group**[1] |
| --- | --- | --- |
| Austin Health, Victoria, Australia | Austin Health Human Research Ethics Committee | Principal referral hospital |
| Peter MacCallum Cancer Centre, Victoria, Australia | Austin Health Human Research Ethics Committee | Other acute specialised hospitals |
| Albury Wodonga Health, Victoria, Australia | Austin Health Human Research Ethics Committee | Public acute group A hospital |
| St George Hospital, New South Wales, Australia | Austin Health Human Research Ethics Committee | Principal referral hospital |
| Royal North Shore Hospital, New South Wales, Australia | Austin Health Human Research Ethics Committee | Principal referral hospital |
| Royal Perth Hospital, Western Australia, Australia | Austin Health Human Research Ethics Committee | Principal referral hospital |
| Monash Health, Victoria, Australia | Austin Health Human Research Ethics Committee | Principal referral hospital |
| Groote Schuur Hospital, Western Cape, South Africa | University of Cape Town Faculty of Health Sciences Human Research Ethics Committee | Principal referral hospital*** |
| Eastern Health, Victoria, Australia | Austin Health Human Research Ethics Committee | Public acute group A hospital |
| Fiona Stanley Hospital, Western Australia, Australia | Austin Health Human Research Ethics Committee | Principal referral hospital |
| Royal Adelaide Hospital, South Australia, Australia | Austin Health Human Research Ethics Committee | Principal referral hospital |
| Barwon Health, Victoria, Australia | Austin Health Human Research Ethics Committee | Principal referral hospital |
| Redcliffe Hospital, Queensland, Australia | Austin Health Human Research Ethics Committee | Public acute group A hospital |
| Sunshine Coast University Hospital, Queensland, Australia | Austin Health Human Research Ethics Committee | Principal referral hospital |
| Lismore Base Hospital, New South Wales, Australia | Austin Health Human Research Ethics Committee | Public acute group A hospital |
| Gold Coast Hospital and Health Services, Queensland, Australia | Austin Health Human Research Ethics Committee | Principal referral hospital |
| Westmead Hospital, New South Wales, Australia | Austin Health Human Research Ethics Committee | Principal referral hospital |
| Armadale Health Service, Western Australia, Australia | Austin Health Human Research Ethics Committee | Public acute group A hospital |
| Bendigo Health, Victoria, Australia | Austin Health Human Research Ethics Committee | Public acute group A hospital |
| Campbelltown Hospital, New South Wales, Australia | Austin Health Human Research Ethics Committee | Public acute group A hospital |
| Grampians Health Service, Victoria, Australia | Austin Health Human Research Ethics Committee | Public acute group A hospital |
| Mildura Public Health Service, Victoria, Australia | Austin Health Human Research Ethics Committee | Regional and remote |
| East Grampians Health Service, Victoria, Australia | Austin Health Human Research Ethics Committee | Public acute group C hospital |
| Royal Prince Alfred Hospital, New South Wales, Australia | Austin Health Human Research Ethics Committee | Principal referral hospital |
| Launceston General Hospital, Tasmania, Australia | Austin Health Human Research Ethics Committee | Public acute group A hospital |
| Alfred Health, Victoria, Australia | Austin Health Human Research Ethics Committee | Principal referral hospital |
| Universiti Malaya Medical Centre, Selangor, Malaysia | Medical Research Ethics Committee, Universiti Malaya Medical Centre | Principal referral hospital*** |
| Latrobe Regional Health, Victoria, Australia | Austin Health Human Research Ethics Committee | Public acute group A hospital |
| Royal Cornwall Hospital, Cornwall, United Kingdom | Health Research Authority and Health and Care Research Wales, Cornwall, United Kingdom | Public acute group A hospital*** |
| Western Health, Victoria, Australia | Austin Health Human Research Ethics Committee | Public acute group A hospital |
| Royal Brisbane and Women’s Hospital, Queensland, Australia | Austin Health Human Research Ethics Committee | Principal referral hospital |
| Queen Mary Hospital, Southern District, Hong Kong | Institutional Review Board of the University of Hong Kong/Hospital Authority Hong Kong West Cluster | Principal referral hospital*** |
| Mitchells Plain Hospital, Western Cape, South Africa | University of Cape Town Faculty of Health Sciences Human Research Ethics Committee | Public acute group B hospital*** |
| McGill University Health Centre, Montreal, Canada | McGill University Health Centre (MUHC) Research Ethics Board | Principal referral hospital*** |
| West Moreton Health, Queensland, Australia | Austin Health Human Research Ethics Committee | Public acute group A hospital |
| Duke University Medical Centre, North Carolina, United States of America | Duke Health Institutional Review Board | Principal referral hospital*** |
| St John of God Hospital Subiaco, Western Australia, Australia | St John of God Health Care Human Research Ethics Committee | Private acute group B hospital |
| King’s College Hospital, Greater London, United Kingdom | Health Research Authority and Health and Care Research Wales, Cornwall, United Kingdom | Principal referral hospital*** |
| Central Gippsland Health, Victoria, Australia | Austin Health Human Research Ethics Committee | Public acute group A hospital |
| Northeast Health Wangaratta, Victoria, Australia | Austin Health Human Research Ethics Committee | Public acute group A hospital |
| Orange Hospital, New South Wales, Australia | Austin Health Human Research Ethics Committee | Public acute group A hospital |
| Royal Melbourne Hospital, Victoria, Australia | Austin Health Human Research Ethics Committee | Principal referral hospital |
| Middlemore Hospital, Auckland Region, New Zealand, | Northern B Health and Disability Ethics Committees, Auckland, New Zealand | Principal referral hospital*** |
| London North West University Healthcare, Greater London, United Kingdom | Health Research Authority and Health and Care Research Wales, Cornwall, United Kingdom | Principal referral hospital* |
| Vanderbilt University Medical Center, Tennessee, United States of America | Vanderbilt University Institutional Review Board (pending) | Principal referral hospital* |

*Assigned an Australian hospital peer group classification in accordance with definition described in the Australian Hospital Peer Groups Report considering patient volume, provision of services, specialist facilities and geographical location[1]

**Reference:**

1. Australian Institute of Health and Welfare Australian hospital peer groups. Health services series no. 66. Cat. no. HSE 170. Canberra: Australian Institute of Health and Welfare; 2015 [Available from: <https://www.aihw.gov.au/getmedia/79e7d756-7cfe-49bf-b8c0-0bbb0daa2430/14825.pdf?v=20230605174748&inline=true>.
