# Supplementary material for: International Network of Antibiotic Allergy Nations (iNAAN): Protocol for a type 2 hybrid effectiveness-implementation multicentre prospective cohort and target trial emulation study evaluating penicillin allergy delabeling via direct oral challenge
Source: PLoS One. 2025 Sep 5;20(9):e0330724. doi: 10.1371/journal.pone.0330724 (PMC12412947; doi:10.1371/journal.pone.0330724)
Supplement: S2 Table — (DOCX) [file pone.0330724.s003.docx]

**S2 Table. Ethnicity and race sub-categories for participating iNAAN countries**

| **Country** | **Ethnicity +/- Race Sub-categories** |
| --- | --- |
| Australia | Caucasian  East Asian  Indo Asian  African  Hispanic or Latino  Aboriginal or Torres Strait Islander  Other (specify) |
| South Africa | Caucasian  East Asian  Indo Asian  African  Mixed race  Other (specify) |
| Malaysia | Malay  Chinese  Indian  Other (specify) |
| United Kingdom | White – British  White – Irish  White – Any other White background  Mixed – White and Black Caribbean  Mixed – White and Black African  Mixed – White and Asian  Mixed – Any other Mixed background  Asian or Asian British – Indian  Asian or Asian British – Pakistani  Asian or Asian British – Bangladeshi  Asian or Asian British – Any other Asian background  Black or Black British – Caribbean  Black or Black British – African  Black or Black British – Any other Black background  Other Ethnic Groups – Chinese  Other Ethnic Groups – Any other Ethnic group  Not stated  Other (specify) |
| Hong Kong | Chinese  Indonesian  Filipino  Caucasian  Indian  Pakistani  Other (specify) |
| Canada | Caucasian  East Asian  Indo Asian  African  Hispanic or Latino  Aboriginal  Other (specify) |
| United States of America | *Ethnicity*  Hispanic or Latino  Not Hispanic or Latino  Unknown  *Race*  American Indian or Alaska Native  Asian  Black or African American  Native Hawaiian or Other Pacific Islander  White  Other (specify) |
| New Zealand | NZ European  NZ Māori  Pacific peoples  Asian  Middle Eastern / Latin American / African  Other (specify) |
