# Supplementary figures and images for: International Network of Antibiotic Allergy Nations (iNAAN): Protocol for a type 2 hybrid effectiveness-implementation multicentre prospective cohort and target trial emulation study evaluating penicillin allergy delabeling via direct oral challenge
Source: PLoS One. 2025 Sep 5;20(9):e0330724. doi: 10.1371/journal.pone.0330724 (PMC12412947; doi:10.1371/journal.pone.0330724)

# NAAN Smartphone Decision Support Logic Implementation

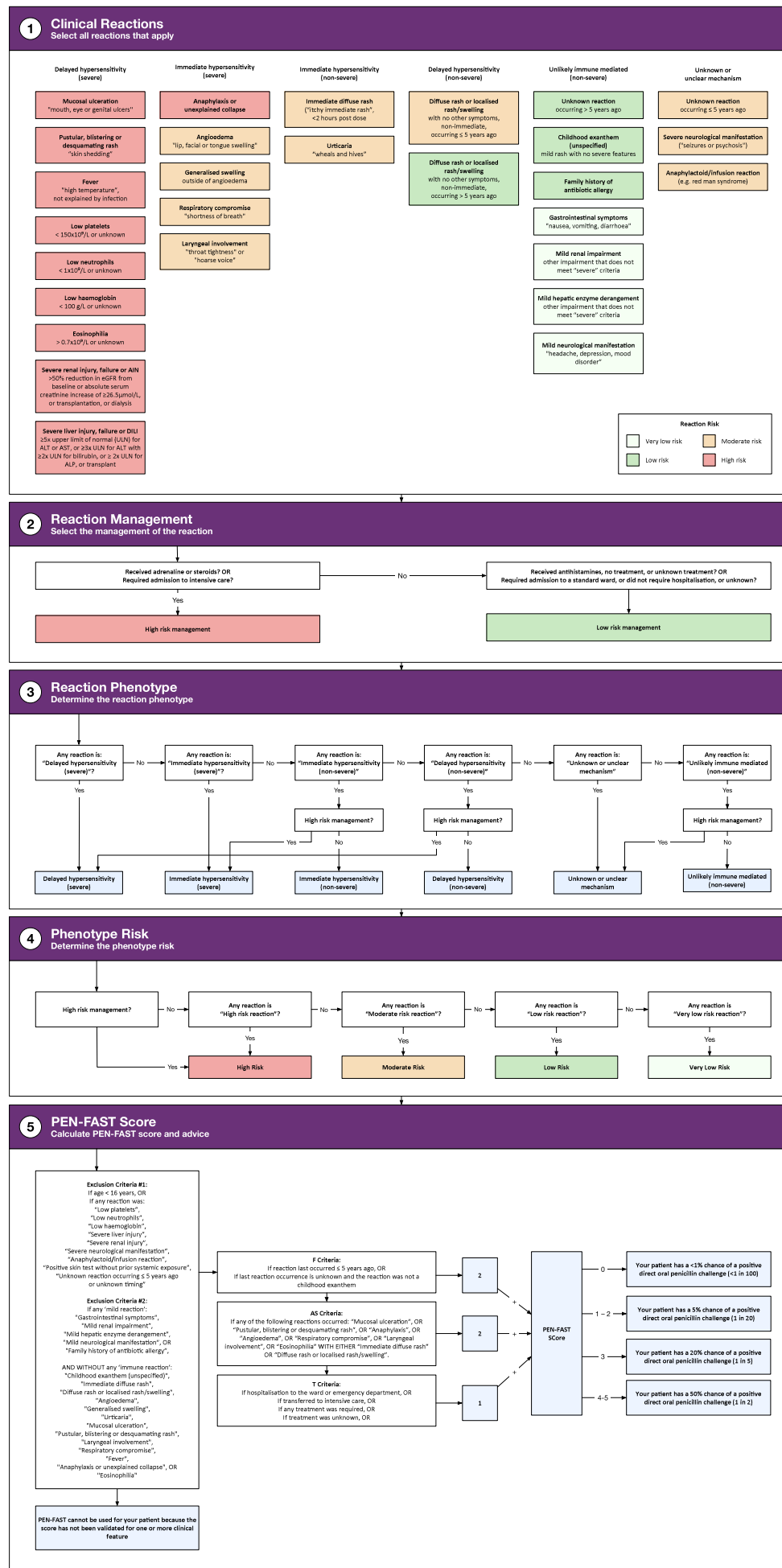

Supplement: S1 Fig — (PDF) [file pone.0330724.s004.pdf]

**S2 Fig. iNAAN Health Service Report Example**


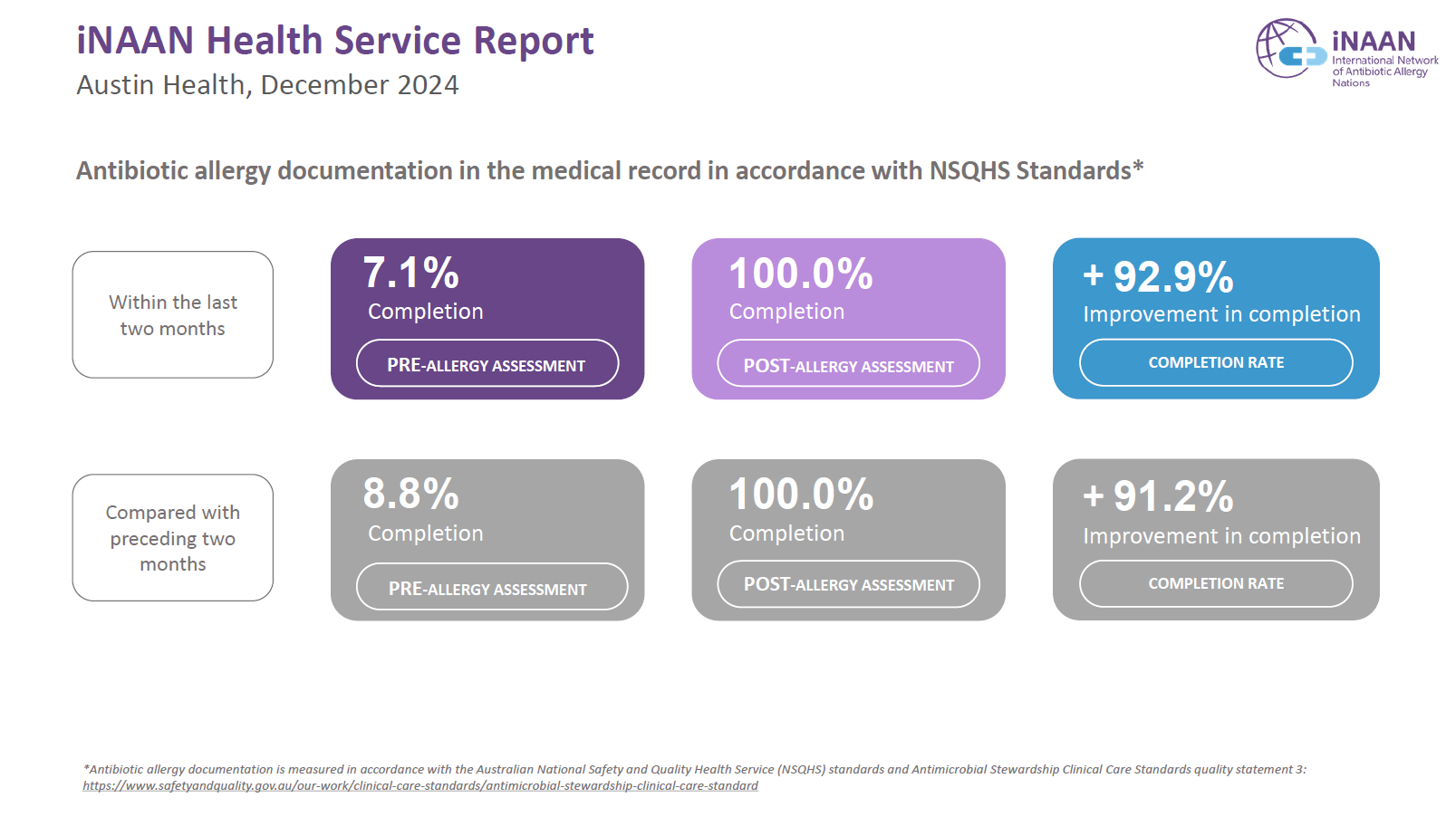

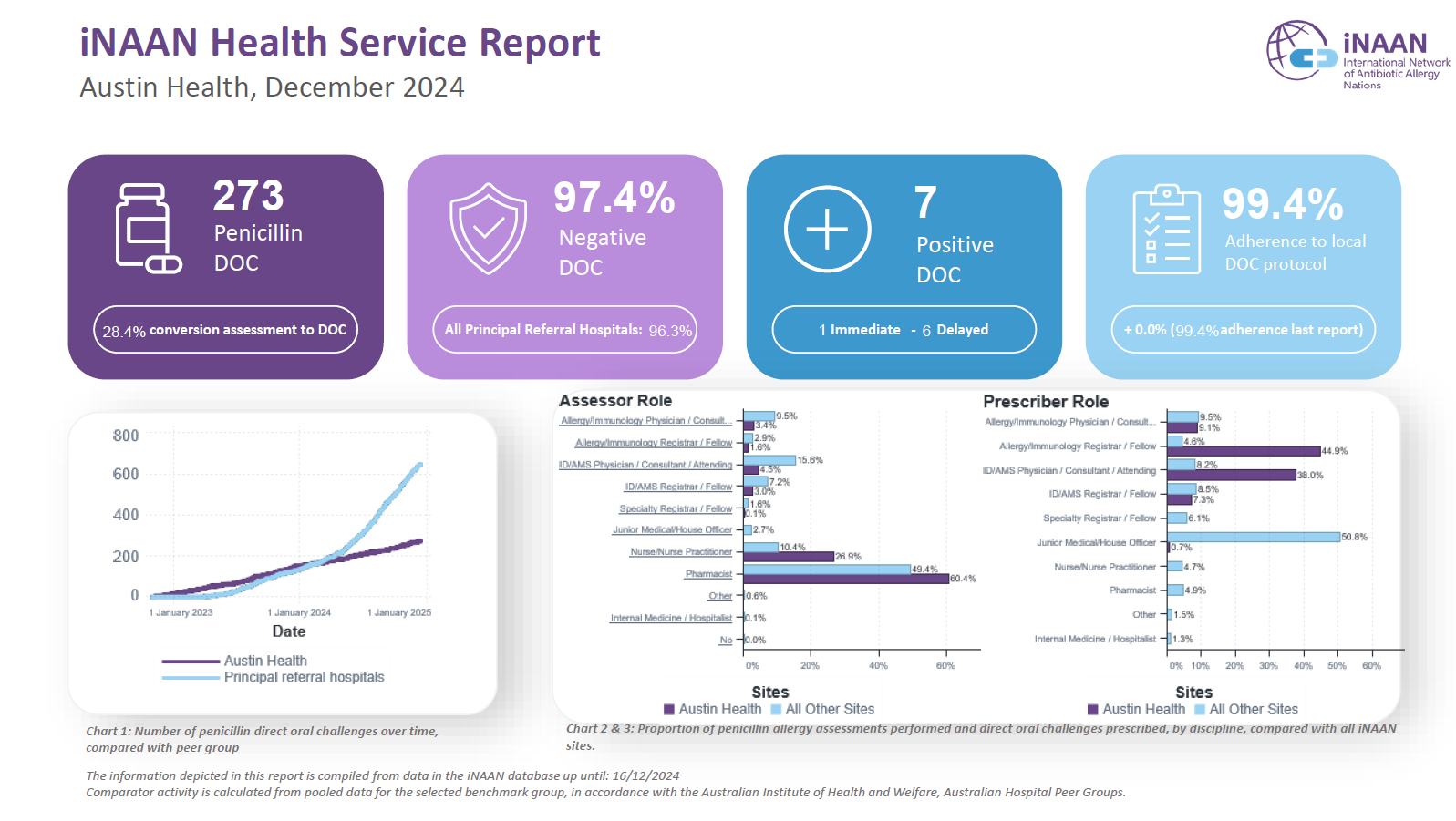

Supplement: S2 Fig — (DOCX) [file pone.0330724.s006.docx]
