# Supplementary material for: International Network of Antibiotic Allergy Nations (iNAAN): Protocol for a type 2 hybrid effectiveness-implementation multicentre prospective cohort and target trial emulation study evaluating penicillin allergy delabeling via direct oral challenge
Source: PLoS One. 2025 Sep 5;20(9):e0330724. doi: 10.1371/journal.pone.0330724 (PMC12412947; doi:10.1371/journal.pone.0330724)
Supplement: S1 Manual — (DOCX) [file pone.0330724.s005.docx]

**S1 Manual. iNAAN Implementation Manual**


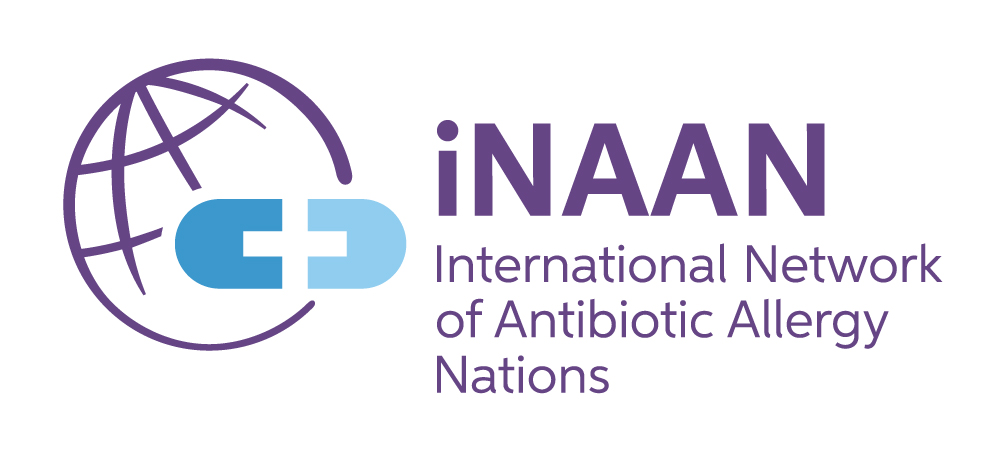


International Network of Antibiotic Allergy Nations (iNAAN)

Implementation Manual

Version 4.0

Date: 12 February 2025

**Introduction**

This manual describes the implementation component of the International Network of Antibiotic Allergy Nations (iNAAN) study. This study will evaluate the effectiveness of a digital penicillin allergy toolkit (clinical intervention) together with implementation strategies (an audit and feedback report and/or education sessions) to aid implementation and uptake of the toolkit in Australian and international health services.

The digital penicillin allergy toolkit is a smartphone app (“NAAN App”) which has been built to permit data collection from study assessors, enabling clinicians to automatically risk assess and phenotype a patient’s penicillin allergy using decision support logic. The NAAN App enables secure upload of allergy assessment data and the results of any subsequent direct oral challenge procedure to the iNAAN REDCap database hosted by BioGrid.

The audit and feedback report (referred to as Health Services Report or HSR) uses health services benchmarking, using the Australian Institute of Health and Welfare hospital peer group categories to provide information on outcome measures. Audit and feedback has been shown to be an effective strategy to modify healthcare professionals’ clinical practice [1]. The HSR will be delivered a minimum of three to six months after site activation, via email to investigator teams and users at participating health services every second month for the duration of the iNAAN study.

Internal facilitation delivered as optional education sessions will be conducted by the iNAAN study team and open to all investigators and stakeholders.

**Study Design**

The iNAAN implementation trial uses a mixed method, pre-post-test Hybrid Type 2 effectiveness-implementation design. Effectiveness-implementation hybrid trials examine both effectiveness and implementation outcomes within the same study and, a priori, take a dual focus on both the impact of implementation factors alongside the impact of the intervention/innovation [2]. Baseline data will be compared with outcomes at 6-months and 12-months.

The implementation framework underpinning the study design is the Framework of Implementability [3] which recommends exploring acceptability, fidelity, and feasibility. These factors are proposed to influence sustainability and scalability. Constructs from the Consolidated Framework for Implementation Research (CFIR) [4] (primarily inner setting and individuals) will be used to explore contextual influences.

**Study aims**

**Clinical effectiveness aims**

1. To determine the clinical effectiveness of a validated point-of-care digital penicillin allergy toolkit (the intervention).

**Implementation aims**

1. To determine if an audit and feedback implementation strategy, delivered digitally, for a validated point-of-care digital penicillin allergy toolkit, influences uptake.

2. To determine if an internal facilitation (education) implementation strategy influences uptake.

3. Explore contextual factors that may influence uptake of the digital penicillin allergy toolkit.

4. To identify strategies that may aid national scaling and implementation of the intervention across different health system that address feasibility, cost, barriers and enablers.

**Outcome measures**

The following outcomes will be assessed during the project:

**Clinical effectiveness outcomes**

**Primary:** proportion of patients discovered with a low-risk penicillin allergy that are delabeled following direct oral (DOC)

**Secondary:**

1. Penicillin utilisation pre-testing (at index admission) vs post-testing (up to 90 days post-discharge). Penicillin utilisation within 90 days of penicillin allergy assessment in patients who undergo testing compared with patients that do not undergo testing
2. Narrow spectrum beta-lactam utilisation pre-testing (at index admission) vs post-testing (up to 90 days post-discharge). Narrow spectrum beta-lactam utilisation within 90 days of penicillin allergy assessment in patients who undergo testing compared with patients that do not undergo testing
3. Restricted antibiotic utilisation pre-testing (at index admission) vs post-testing (up to 90 days post-discharge). Restricted antibiotic utilisation within 90 days of penicillin allergy assessment in patients who undergo testing compared with patients that do not undergo testing
4. Appropriate antibiotic utilisation pre-testing (at index admission) vs post-testing (up to 90 days post-discharge). Appropriate antibiotic utilisation within 90 days of penicillin allergy assessment in patients who undergo testing compared with patients that do not undergo testing
5. Median hospital LOS (days, IQR) for patients with a low-risk penicillin allergy that were delabeled versus patients with a penicillin allergy that are not delabeled
6. To determine the cost-effectiveness of the program model and implementation strategy

**Implementation outcomes (including definitions)**

**Primary:** Adoption - adoption (i.e. uptake) of the intervention by clinicians within participating sites and hospitals across Australia i.e: number of multi-disciplinary clinicians and health services with access to the ‘NAAN App’ from baseline to 6 months.

**Secondary:**

1. Acceptability: defined as “a multi-faceted construct that reflects the extent to which people delivering or receiving a healthcare intervention consider it to be appropriate, based on anticipated or experienced cognitive and emotional responses to the intervention” [5] i.e: perception amongst participating health clinicians that the penicillin allergy toolkit is agreeable.
2. Fidelity: for the purpose of this study defined as “compliance of penicillin DOC activity in accordance with individual health service protocol” (referred to as ‘compliance’ in the HSR) i.e: Proportion of penicillin DOC procedures (intervention) that are adherent to the audited health service local delabeling protocol
3. Adherence: for the purpose of this study defined as “adherence to the Australian National Safety and Quality Health Service (NSQHS) Standards for recording penicillin allergy documentation in the medical record, both pre- and post- allergy assessment”.
4. Feasibility: the extent to which the NAAN App can be successfully used within a given context [6]
5. Sustainability: defined as “after a defined period of time, a program, clinical intervention, and/or implementation strategy continue to be delivered (or the individual behaviour change) is maintained” [7]. In the case of the iNAAN project, the defined number of App users over a period baseline to 6 months and then to 12 months.
6. To develop recommendations for national scaling and implementation of the program across the health system (based on feasibility, costs, barriers and facilitators)

**Method and data collection – clinical intervention effectiveness data**

**Method:** The effectiveness of the digital penicillin allergy toolkit will be measured using the pre-defined effectiveness outcome measures. Data will be obtained from all sites enrolled in the study using the iNAAN REDCap database. Full details of the iNAAN study can be found in Protocol 78719 v3.2.

**Method – implementation intervention**

**Implementation strategies**

**Audit and feedback strategy- Health Services Report**

**Method:** Feedback via a Health Services Report (HSR) is provided to named investigators and NAAN App users (see Appendix A) and aims to increase uptake of the toolkit by providing feedback on their performance [1]. The implementation strategy will be consistently delivered across all sites and outcomes will be compared within and between sites, accounting for context, for example, within hospital peer group and clinical disciplines.

Following site initiation and commencement of the iNAAN study, no audit and feedback will be provided to clinicians and health services for a minimum of 3-6 months post site activation.

**Education / internal facilitation**

Internal facilitation delivered as optional education sessions will be conducted by the iNAAN study team and open to all investigators and stakeholders. Attendance data will be collected and used to compare implementation outcomes at sites or, where appropriate, compare clinicians. This comparison data may provide insights into whether education influences uptake and could be used as a future implementation strategy.

**Data Collection and Analysis – Implementation intervention**

| **1. ADOPTION** | **Who** | **Framework** | **How** | **Timepoints** |
| --- | --- | --- | --- | --- |
| **To determine the adoption (i.e. uptake) of the toolkit** | Number of NAAN App users  Assess discipline-specific uptake and use; number of medical practitioners (by specialty), number of pharmacists, number of nurses, number of others.  Individual health service enrolment by jurisdiction and country  Number of countries with a participating health service | Quantitative (number of) | File audit | Baseline  6 months (i.e. post implementation strategy) |
| **Data analysis** | Quantify categorical data using frequencies and percentages.  Disaggregated data by clinical group, hospital peer group, geographical location as appropriate.  Measure the change from baseline to 6 months. | | | |

| **2A. ACCEPTABILITY** | **Who** | **Framework** | **How** | **Timepoints** |
| --- | --- | --- | --- | --- |
| **2a. Perceived acceptability of the NAAN App** | NAAN App users | Theoretical framework of acceptability [5] | TFA Questionnaire (Appendix B) via NAAN App | Survey will trigger a minimum of 6-months after user’s first use, then 6-monthly ongoing  Survey will not trigger if App is not being used  Survey will trigger after patient care, and data will transfer to REDcap. |
| **Data analysis** | Descriptive statistics will be used to document the acceptability of the NAAN App across the seven TFA component constructs and the one general acceptability question.  Free text responses will be thematically analysed and organised into categories or domains identified through analysis. | | | |
| **Data collection tool** | See Appendix B - Acceptability of NAAN App | | | |

**Rationale for assessing NAAN App Acceptability**

Intervention acceptability is an important consideration in the development and appraisal of healthcare interventions as it is likely linked to engagement with the intervention [8]. Further, acceptability is a likely precursor to intervention sustainability [3]. Obtaining information regarding acceptability of the toolkit will provide useful data to inform engagement with, and possible modifications needed, to the intervention. Acceptability of the NAAN App will be assessed using a theory informed questionnaire (Theoretical Framework of Acceptability) via the NAAN App (see Appendix B).

**Data collection method for assessing NAAN App Acceptability**

A NAAN App acceptability questionnaire will trigger a minimum of 6-months post site activation, on a 6-monthly basis, to NAAN App users. The questionnaire is a theory-informed, validated questionnaire comprising of eight questions using 5-point Likert scale responses. It is designed to measure clinicians’ perceptions of acceptability of the NAAN App, which encompasses the embedded penicillin allergy toolkit.

The questionnaire will be delivered via the NAAN App and data will be collected and stored in the existing iNAAN REDCap database, hosted by BioGrid Australia. Results will be provided to the iNAAN study team but will not be disseminated back to health services.

| **2B. ACCEPTABILITY** | **Who** | **Framework** | **How** | **Timepoints** |
| --- | --- | --- | --- | --- |
| **2b. Perceived Acceptability of the iNAAN HSR Report** | Recipients of the HSR ie., iNAAN investigators, and NAAN App users | Theoretical framework of acceptability [5] | TFA Questionnaire (Appendix C) via RedCap survey link delivered via email | Concurrent with HSR reporting |
| **Data analysis** | Descriptive statistics will be used to document the acceptability of the HSR across the seven TFA component constructs and the one general acceptability question.  Free text responses will be thematically analysed and organised into categories or domains identified through analysis. | | | |
| **Data collection tool** | See Appendix C - Acceptability of HSR | | | |

**Rationale for assessing HSR Acceptability**

Acceptability of the HSR will be investigated using a theory informed questionnaire (Theoretical Framework of Acceptability) (see Appendix C) with a similar rationale as above. Engagement with the HSR is likely to be linked to perceptions of acceptability of those using this tool. Obtaining information regarding acceptability of the HSR will provide useful data to inform adaption or modification of the audit and feedback report.

**Data collection method for assessing NAAN App Acceptability**

The HSR acceptability questionnaire will be sent to investigators along with the HSR on a bi-monthly basis. The REDCap questionnaire will be delivered via email and data will be collected and stored in the existing iNAAN REDCap database, hosted by BioGrid Australia. Results will be provided to the iNAAN study team and disseminated back to health services when/if changes to the HSR report are made.

| **3. FIDELITY** | **Who** | **Framework** | **How** | **Timepoints** |
| --- | --- | --- | --- | --- |
| Were penicillin DOC procedures consistent with local penicillin allergy delabeling clinical protocols? | Sites | N/A | File audit via NAAN App data collection  Matches to Hospital protocol in REDcap | Baseline  6-months  12-months |
| **Data analysis** | Quantitative data obtained from iNAAN database | | | |

| **4. ADHERENCE** | **Who** | **Framework** | **How** | **Timepoints** |  |
| --- | --- | --- | --- | --- | --- |
| Pre-assessment, what proportion of patients at participating sites had the implicated drug, date, nature, and severity of the penicillin allergy documented in the medical record?  Post-assessment, what proportion of patients at participating sites had the implicated drug, date, nature, and severity of the penicillin allergy documented in the medical record? | Site-based data  Australian health services only | National Safety and Quality Health Service Standards (NSQHS) | File audit via NAAN App data collection | Ongoing/continuous | |
| **Data analysis** | Quantitative data obtained from iNAAN database | | | | |

| **5. FEASIBILITY** | **Who** | **Framework / Tool** | **How** | **Timepoints** |
| --- | --- | --- | --- | --- |
| Determine the extent to which the NAAN App can be successfully used within a given context. | All investigators and clinicians with access to the NAAN App to ensure a cross section of clinicians using NAAN App.  Consider collecting data from other hospital stakeholders eg., AMS, EMR, Immunology, Medication Safety, Gen Medicine, Director Pharmacy | Feasibility of Intervention Measure (FIM) [9] | Survey | Distributed to NAAN App users at least 3 months post site activation and after at least 1 participant has been entered into the iNAAN database. |
| **Data analysis** | Quantitative analysis of questionnaire responses.  Qualitative analysis of open text responses  Scales can be created for each measure by averaging responses. Scale values range from 1 to 5. Cut-off scores for interpretation not yet available; however, higher scores indicate greater feasibility. | | | |
| **Data collection tool** | Appendix D | | | |

| **6. SUSTAINABILITY** | **Who** |  | **How** | **Timepoints** |  |
| --- | --- | --- | --- | --- | --- |
| Adoption at 6 months and at 12months  *Has the intervention become routine practice?* | Number of NAAN App users  Assess discipline-specific uptake and use; number of medical practitioners (by specialty), number of pharmacists, number of nurses, number of others  Individual health service enrolment by jurisdiction and country  Number of countries with a participating health service | | File audit / quantitative | Baseline  6 months  12 months | |
| **Data analysis** | Categorical data will be quantified using frequencies and percentages.  Data will disaggregate by clinical group, hospital peer group, geographical location as appropriate.  Data collected at baseline will be compared to timepoint at 6 months and at 12 months. | | | | |

**Additional qualitative data: HSR Evaluation and Influencing Contextual Factors**

**A: Evaluation of the Audit and Feedback Report (HSR)**

Semi-structured focus groups will be undertaken with HSR users to obtain their feedback regarding the HSR. Participants will be asked questions about the content and their use of the HSR. This information will be used to inform changes to the HSR according to stakeholder needs.

| **CONTEXT** | **Who** | **Framework** | **How** | | **Timepoints** |
| --- | --- | --- | --- | --- | --- |
| Obtain feedback regarding the content of the HSR and how users are engaging with or utilising HSR information. | HSR users – investigators and clinicians | Theoretical Domains Framework [10] | Semi-structured focus groups  Content analysis of interview transcripts | 2-12 months post receipt of HSR | |
| **Data analysis** | Content analysis of focus group transcripts | | | | |
| **Data collection tool** | See Appendix E – HSR stakeholder focus group | | | | |

**B: Contextual influences**

Contextual influences will be gathered throughout the study period to explore factors which may impact implementation. Constructs from the Consolidated Framework for Implementation Research (CFIR) [4] (primarily inner setting and individuals) will be used to organise this data.

| **CONTEXT** | **Who** | **Framework** | **How** | | **Timepoints** |
| --- | --- | --- | --- | --- | --- |
| Which elements of context impact adoption, fidelity, or other outcomes? | Consider:  Organisation site, staffing profile, financial support | Consolidated Framework for Implementation Research (CFIR) | Deductive content analysis of study documents from each site | Baseline | |
| Which elements of context impact adoption, fidelity, or other outcomes? | Consider:  Partnerships and connection  Compatibility with other systems  Culture | Consolidated Framework for Implementation Research (CFIR) | Descriptive information identified during discussion and data collection | Ongoing | |

**References**

1. Ivers N, Jamtvedt G, Flottorp S, Young JM, Odgaard-Jensen J, French SD, et al. Audit and feedback: effects on professional practice and healthcare outcomes. Cochrane Database Syst Rev. 2012;2012(6):Cd000259.

2. Bauer MS, Damschroder L, Hagedorn H, Smith J, Kilbourne AM. An introduction to implementation science for the non-specialist. BMC Psychol. 2015;3(1):32.

3. Klaic M, Kapp S, Hudson P, Chapman W, Denehy L, Story D, et al. Implementability of healthcare interventions: an overview of reviews and development of a conceptual framework. Implement Sci. 2022;17(1):10.

4. Damschroder LJ, Reardon CM, Widerquist MAO, Lowery J. The updated Consolidated Framework for Implementation Research based on user feedback. Implement Sci. 2022;17(1):75.

5. Sekhon M, Cartwright M, Francis JJ. Development of a theory-informed questionnaire to assess the acceptability of healthcare interventions. BMC Health Serv Res. 2022;22(1):279.

6. Proctor E, Silmere H, Raghavan R, Hovmand P, Aarons G, Bunger A, et al. Outcomes for implementation research: conceptual distinctions, measurement challenges, and research agenda. Adm Policy Ment Health. 2011;38(2):65-76.

7. Moore JE, Mascarenhas A, Bain J, Straus SE. Developing a comprehensive definition of sustainability. Implement Sci. 2017;12(1):110.

8. Skivington K, Matthews L, Simpson SA, Craig P, Baird J, Blazeby JM, et al. A new framework for developing and evaluating complex interventions: update of Medical Research Council guidance. Bmj. 2021;374:n2061.

9. Weiner BJ, Lewis CC, Stanick C, Powell BJ, Dorsey CN, Clary AS, et al. Psychometric assessment of three newly developed implementation outcome measures. Implement Sci. 2017;12(1):108.

10. Atkins L, Francis J, Islam R, O'Connor D, Patey A, Ivers N, et al. A guide to using the Theoretical Domains Framework of behaviour change to investigate implementation problems. Implement Sci. 2017;12(1):77.

11. Ritchie J LJ, Nicholls C, Ormston R Qualitative Research Practice. 2nd ed. London, United Kingdom: Sage Publications Ltd; 2014.

**Appendix A – iNAAN Health Service Reports**

The iNAAN SAS Visual Analytics Reporting Platform, hosted by BioGrid Australia, will be utilised to produce reports that will be delivered bi-monthly, as part of the audit and feedback intervention, commencing from at least 3-6 months post site initiation.

The development of the content and format of the iNAAN Health Service Report has been in consultation with the iNAAN Steering Committee and an investigator working group to ensure the reports meet the needs of investigator teams and thus optimises opportunities for improvement of the clinical intervention at a local health service level.

Comparator activity is calculated from pooled data for the selected benchmark group, in accordance with the Australian Institute of Health and Welfare, Australian Hospital Peer Groups publication.

**Reporting outcomes within the digitally delivered audit and feedback (iNAAN HSR)**

Data source for all reporting outcomes: REDCap

**Clinical effectiveness outcomes**

1. Number of penicillin DOC performed
2. Proportion (%) of penicillin allergy assessments converted to a DOC
3. Safety - Proportion (%) of penicillin DOC that were negative
   - Benchmarked against hospital peer group
4. Number of positive penicillin DOC
   - Stratified by number of immediate positive DOC and number of delayed positive DOC

**Implementation outcomes**

**Adoption – penicillin allergy activity delivered by discipline**

1. Proportion (%) penicillin allergy assessments performed by discipline at the health service
2. Proportion (%) penicillin allergy assessments prescribed by discipline at the health service
   - Benchmarked against all iNAAN health services

**Fidelity**

- - - 1. Proportion (%) of penicillin DOC that are compliance to the individual health service clinical protocol
- Includes evaluation of choice of drug, challenge dose, single vs split dose challenge, single vs prolonged dose challenge
  - - - - Benchmarked against self (previous HSR)

**Adherence**

1. Proportion (%) of penicillin allergy assessments with that adhere to the Australian National Safety and Quality Health Service (NSQHS) Standards for penicillin allergy documentation in the medical record i.e: complete and accurate implicated drug, date of reaction, nature and severity of reaction
   - - Measured PRE- and POST- penicillin allergy assessment
       - - Benchmarked against self (previous HSR)

**Sustainability -**

1. Number penicillin DOC over time
   - - - - Benchmarked against hospital peer group

**Note: For the purposes of the iNAAN HSR, health service benchmarking priorities are as follows:**

**Priority 1:** Benchmark against hospital peer group, providing there is at least 1 other comparator

**Priority 2:** For iNAAN sites that don't have a comparator within their hospital peer group, benchmark against state/province, providing there is at least 1 other comparator within that state/province.

**Appendix B – NAAN App Acceptability Questionnaire**

| **Acceptability Questionnaire**  We would appreciate your time to answer eight questions regarding the NAAN App. These questions assess different constructs of acceptability. There is space at the end of the questionnaire for you to provide any other feedback. We will use this information to inform implementation and optimisation of the NAAN App. Completion of this survey will indicate that you are providing implied consent to participate in the survey.  **Q1: Do you like or dislike the NAAN App?**  Options: 1 Strongly dislike / 2 Dislike / 3 No opinion / 4 Like / 5 Strongly like  **Q2: How much effort does it take to use the NAAN App?**  Options: 1 No effort at all / 2 A little effort / 3 No opinion / 4 A lot of effort / 5 Huge effort  **Q3: There are moral or ethics consequences to using/engaging with the NAAN App?**  Options: 1 Strongly disagree / 2 Disagree / 3 No opinion / 4 Agree / 5 Strongly agree  **Q4: The NAAN App has improved my ability to perform a point of care penicillin allergy risk assessment and consider oral challenge as a potential delabeling strategy in appropriately assessed patients**  Options: 1 Strongly disagree / 2 Disagree / 3 No opinion / 4 Agree / 5 Strongly agree  **Q5: It is clear to me the NAAN App will help improve access to antibiotic allergy assessment and appropriate penicillin allergy delabeling strategies?**  Options: 1 Strongly disagree / 2 Disagree / 3 No opinion / 4 Agree / 5 Strongly agree  **Q6: How confident do you feel about using the NAAN App?**  Options: 1 Very unconfident / 2 Unconfident / 3 No opinion / 4 Confident / 5 Very confident  **Q7: The NAAN App interfered with my other priorities.**  Options: 1 Strongly disagree / 2 Disagree / 3 No opinion / 4 Agree / 5 Strongly agree  **Q8: How acceptable is the NAAN App to you?**  Options: 1 Completely unacceptable / 2 Unacceptable / 3 No opinion / 4 Acceptable / 5 Completely Acceptable  **Q9: Do you have other feedback or comments about the NAAN App?**  Freetext response |
| --- |

**Appendix C – HSR Acceptability Questionnaire**

| **Acceptability Questionnaire**  The iNAAN study uses a digitally delivered Audit and Feedback implementation strategy. Feedback is provided via a Health Services Report (HSR) to participating investigators and non-investigator clinicians at each site. An iNAAN HSR acceptability survey, based on the Theoretical Framework of Acceptability (TFA) questionnaire [5], will be delivered electronically to participating iNAAN investigators and non-investigator clinicians with the accompanying HSR. The survey is designed to assess different constructs of acceptability pertaining to the iNAAN HSR and to enable an avenue to provide feedback. The information will inform changes to the HSR.  The online survey will be conducted via REDCap, hosted by BioGrid Australia to meet data management requirements, and takes approximately 3-5 minutes to complete.  **Q1: Which iNAAN health service do you work in?**  **Q2: Please select your role in the iNAAN study at your health service**  Investigator (with access to REDCap database and the NAAN App) / Non-investigator clinician (with access to NAAN App)  **Q3: What is your healthcare discipline?**  Options: Medical Practitioner / Nurse or Nurse Practitioner / Pharmacist / Other clinician - Specify:  **Q4: Do you like or dislike the Health Services Report (HSR)?**  Options: 1 Strongly dislike / 2 Dislike / 3 No opinion / 4 Like / 5 Strongly like  **Q5: How much effort does it take to use the HSR?**  Options: 1 No effort at all / 2 A little effort / 3 No opinion / 4 A lot of effort / 5 Huge effort  **Q6: There are moral or ethics consequences to using the HSR?**  Options: 1 Strongly disagree / 2 Disagree / 3 No opinion / 4 Agree / 5 Strongly agree  **Q7: The HSR has improved my understanding of how to optimise my use of the NAAN App to positively impact patient care at my hospital?**  Options: 1 Strongly disagree / 2 Disagree / 3 No opinion / 4 Agree / 5 Strongly agree  **Q8: It is clear to me how the HSR will help improve access to antibiotic allergy assessment and appropriate penicillin allergy delabeling strategies?**  Options: 1 Strongly disagree / 2 Disagree / 3 No opinion / 4 Agree / 5 Strongly agree  **Q9: How confident do you feel about using the information in the HSR?**  Options: 1 Very unconfident / 2 Unconfident / 3 No opinion / 4 Confident / 5 Very confident  **Q10: Reading the HSR interfered with my other priorities.**  Options: 1 Strongly disagree / 2 Disagree / 3 No opinion / 4 Agree / 5 Strongly agree  **Q11: How acceptable was the HSR to you?**  Options: 1 Completely unacceptable / 2 Unacceptable / 3 No opinion / 4 Acceptable / 5 Completely Acceptable  **Q12 Please tell us what was the MOST useful information in the HSR for you?**  **Q13 What was the LEAST useful information in the HSR for you?**  **Q14 Do you have other feedback or comments about the HSR? Feedback will inform modifications to the HSR.** |
| --- |

**Appendix D – NAAN App Feasibility Questionnaire**

We are seeking your time to answer this short 2-minute survey to obtain your opinion regarding feasibility of the NAAN App. Feasibility means “the extent to which the NAAN App can be successfully used within a given context” [6]. Feasibility is a factor that is likely to influence sustainability and scalability of the NAAN App.

Please answer the following survey questions utilising the Feasibility of Intervention Measure [9]. Free text responses in relation to your perceptions of feasibility (according to the above definition) are welcome in Question 5.

**Please select the type of health service in which you perform penicillin allergy assessment and delabeling (according to the Australian Institute of Health and Welfare hospital peer groups):**

- - - Principal referral hospital
    - Other specialised hospital
    - Public acute group A hospital
    - Public acute group B hospital
    - Public acute group C hospital
    - Private acute group A hospital
    - Private acute group B hospital
    - Regional and remote hospital

| Feasibility of Intervention Measure (FIM) [9] | Completely disagree | Disagree | Neither agree nor disagree | Agree | Completely agree |
| --- | --- | --- | --- | --- | --- |
| 1. The NAAN App seems implementable. | □ | □ | □ | □ | □ |
| 2. The NAAN App seems possible. | □ | □ | □ | □ | □ |
| 3. The NAAN App seems doable. | □ | □ | □ | □ | □ |
| 4. The NAAN App seems easy to use. | □ | □ | □ | □ | □ |
| 1. Please share anything further regarding your perception of the feasibility of the NAAN App. |  | | | | |

**Appendix E – HSR stakeholder focus group**

**Background**

The health services report (HSR) is the main implementation strategy being applied in the iNAAN implementation trial (a pre-post-test hybrid type 2 implementation trial). The HSR is an audit and feedback tool. The intervention in the iNAAN study is the NAAN App (or ‘toolkit’). The HSR uses health services benchmarking, using the Australian Institute of Health and Welfare hospital peer group categories to provide information on outcome measures. A minimum of three to six months after site activation, feedback will be provided via email to investigator teams and users at participating health services every second month for the duration of the iNAAN study.

Audit and feedback has been chosen as the implementation strategy as it is widely used as a strategy to improve healthcare professionals (HCP) modify their practice when provided with feedback on their performance [1]. A 2012 Cochrane review concluded that audit and feedback generally leads to small but important improvements [1]. Further they report audit and feedback is most effectiveness when: HCP are not performing well to start out with; the person responsible for the audit and feedback is a supervisor or colleague; it is provided more than once; it is given both verbally and in writing; and, it includes clear targets and an action plan.

**Aim**

The implementation strategy (ie., the HSR) aims to increase uptake of the toolkit / NAAN App. The research team seek to understand stakeholders’ and users’ perspectives to revise and improve the HSR.

**Study Design / Methodology**

Focus groups will be undertaken with stakeholders to understand what audit information they would find most useful, and what information is most likely to change their behaviour. Stakeholders will also be asked about perceived barriers and enablers to accessing and using the HSR.

Focus group duration should be limited to 30minutes so that participation is feasible for potential participants. Therefore, consider conducting more than one focus group to keep size to 4-6 participants at each time.

**Sampling, participants, time commitment, and group size**

*Sampling:* Direct recruitment of engaged stakeholders

*Participants:* Project stakeholders – users and investigators (groups could be mixed)

*Consent:* Consent process explained in invitation email. Consent via agreement to record session on Zoom

*Demographic information:* Obtain clinician (profession), investigator (profession)

*Time commitment:* 30 minutes

*Group size:* 4-6 participants per group, likely grouped in consideration of time-zones

**Facilitators**

CIA to introduce session but not attend the whole session.

Implementation science researcher to conduct / facilitate the focus group with support from subject matter expert and project investigator.

**Data Collection - Focus groups**

Preparation - 2 days prior to focus group

Send reminder email and an example HSR report to participants

Opening – stimulus material (prepare slides if useful)

*Permission to record*

*Check participant names on Zoom*

Thank for you attending.

Explain purpose of focus group;

- Explore views of stakeholders of the HSR to inform modification of the current report (eg., data points, information, structure, functionality)
- Using the HSR (audit and feedback) as an implementation strategy to improve uptake of the NAAN toolkit or App
- Provide 2 mins of background on what makes effective A&F [depending on timing]
- Barriers and facilitators to accessing HSR

**Focus group – questions**

*Firstly, we wish to explore the information in the HSR (and what you think is missing)*

- What audit/HSR information did you find most useful? Why?
- What information did you find least useful? Why?
- Was any information was missing?

*Next, we wish to explore what you did (if anything) after receiving the HSR.*

- After reading the HSR, what did you do differently?
- After reading the HSR, can you recall what you INTENDED to do differently? And did you act on your intention?
- [for investigators] what did you do with the HSR information? (prompt; how did you disseminate or feedback to colleagues / team members? Was that verbally or in-writing?)
- What action plan did you generate as a result of the HSR?

*Last question about the function and usability of the actual report.*

- What is MOST important to you in relation to audit information; the type information, the frequency, the ‘look’, the functionality (eg., that it links to other information)
- Any thoughts about what is LEAST important to you?

*A couple of questions to finish up – we’d like to know how you accessed the HSR and possible barriers and enablers to access.*

- How / when / where do you access (or read) the HSR?
- When would be the ideal time for *you* to receive the HSR?

**Data analysis**

Focus group transcripts and field notes will be analysed using content thematic analysis [11]. Data will be reported descriptively to identify data points of interest. Deductive content analysis using the Theoretical Domains Framework [10] constructs to identify barriers and enablers.

**Report findings**

Descriptive findings with illustrative quotes.
