# Supplementary material for: International Network of Antibiotic Allergy Nations (iNAAN): Protocol for a type 2 hybrid effectiveness-implementation multicentre prospective cohort and target trial emulation study evaluating penicillin allergy delabeling via direct oral challenge
Source: PLoS One. 2025 Sep 5;20(9):e0330724. doi: 10.1371/journal.pone.0330724 (PMC12412947; doi:10.1371/journal.pone.0330724)
Supplement: S3 Table — (DOCX) [file pone.0330724.s007.docx]

**S3 Table. iNAAN target trial specification and emulation using observational data**

| **Protocol Component** | **Description under target trial** | **Description under emulation using observational data** |
| --- | --- | --- |
| *Eligibility Criteria* | Include: Adult inpatients assessed as having an immune-mediated or unknown low-risk penicillin allergy label.   - Penicillin defined as: ‘penicillin unspecified’, amoxicillin, ampicillin, phenoxymethylpenicillin, benzylpenicillin, flucloxacillin, amoxicillin-clavulanate, dicloxacillin, cloxacillin - Low-risk defined as: PEN-FAST score < 3 OR ‘green’ on the AAAT*   Exclude:   - Patients that report a Type A or non-immune mediated penicillin allergy labels - Patients that had exposure to any penicillin prior to penicillin allergy assessment during the index hospital admission - Patients that report reaction to piperacillin-tazobactam | As per target trial. Assessed between November 2022 and date of data censure in the iNAAN database. |
| *Treatment Strategies* | Intervention group: Penicillin direct oral challenge (DOC) in the inpatient setting within one week of penicillin allergy assessment date.   - The approach to penicillin DOC, including choice of drug, dose, duration and observation period, is in accordance with the local site protocol.   Comparator group: No penicillin DOC and patient retains penicillin allergy label. | Same as for target trial.   - Participants that were delabeled via any other mechanism e.g: direct delabel, or skin-testing then oral challenge, were excluded from the analysis. - Participants that had an amoxicillin-clavulanate allergy label that did not receive an amoxicillin-clavulanate DOC were excluded from the analysis |
| *Assignment Procedures* | Following penicillin allergy assessment, participants were randomly assigned to either strategy within a week of penicillin allergy assessment. Participants were aware of the strategy to which they were assigned. | Participants who undergo penicillin DOC as per protocol within one week of assessment will be allocated to the intervention arm, while participants who don’t receive a penicillin DOC will be assigned to the control arm. |
| *Outcome* | **Primary**   1. Penicillin utilisation within 90 days of randomisation (Number of participants receiving at least one dose of any penicillin antibiotic, excluding the dose used for DOC)   **Secondary**   1. Narrow-spectrum beta-lactam utilisation within 90 days of randomisation (number of participants receiving at least one dose of narrow spectrum beta lactam antibiotic) 2. Restricted antibiotic**** utilisation within 90 days of randomisation (number of participants receiving at least one dose of narrow spectrum beta lactam antibiotic) 3. Hospital length of stay (days) following randomisation 4. Presence of multi-drug resistant organism*** or *Clostridioides difficile* infection within 90 days of randomisation | As per target trial |
| *Follow-up Period* | 90-days post randomisation | Starts at date of penicillin allergy assessment and ends at 90-days post penicillin allergy assessment. |
| *Statistical Analysis* | Per protocol analysis | Per protocol analysis  Baseline confounders will be selected with the help of direct acrylic graphs and entropy balancing approach will be used to generate weights.  Average treatment effect (ATE) will be estimated in this pseudo population using generalized linear model with binomial family, log link and robust variance estimator. Results will be presented as risk ratios with 95% confidence intervals. |
| *Sub-group analysis* | Immunocompromised patients   - Defined as history of solid organ tumour in the past five years (excluding skin other than melanoma), leukaemia, lymphoma, myeloma, transplant recipient, HIV, asplenia, autoimmune disease, inborn errors of immunity, or iatrogenic immunosuppression within the past three months^†^   Individual PEN-FAST score   - 0 - 2 | As per target trial |

* AAAT: Antibiotic Allergy Assessment Tool: ‘green’ = unknown reaction > 5 years ago, childhood exanthem unspecified (mild rash with no severe features), non-immediate diffuse rash or localised rash/swelling > 5 years ago (with no other symptoms)

** a) Pre-defined restricted antibiotics: 3rd generation or later cephalosporins, fluoroquinolones, glycopeptides, lincosamides, piperacillin-tazobactam, carbapenems

b) Highest priority critically important antimicrobial (HPCIA): 3rd and 4th generation cephalosporins, fluoroquinolones, polymixins, phosphonic acid derivatives (WHO List of Medical Important Antimicrobials: a risk management tool for mitigating antimicrobial resistance due to non-human use, published by the World Health Organization, 2024. Available from: <https://cdn.who.int/media/docs/default-source/gcp/who-mia-list-2024-lv.pdf>)

c) World Health Organization AWaRe ‘Watch’ or ‘Reserve’ antibiotic: Per defined list in WHO Access, Watch, Reserve (AWaRe) classification of antibiotics for evaluation and monitoring of use, published by the World Health Organization, 2021. Available from: <https://iris.who.int/handle/10665/345555>

^***^Vancomycin-resistant enterococcus (VRE), Methicillin-resistant Staphylococcus aureus (MRSA), Multidrug-resistant Gram-negative bacteria

^†^Iatrogenic immunosuppression: prednisolone >0.5mg/kg/day for >14 days, ciclosporin, tacrolimus or sirolimus, azathioprine or mycophenolate, cyclophosphamide, leflunomide or methotrexate, monoclonal antibodies, cancer chemotherapy
