# Supplementary material for: International Network of Antibiotic Allergy Nations (iNAAN): Protocol for a type 2 hybrid effectiveness-implementation multicentre prospective cohort and target trial emulation study evaluating penicillin allergy delabeling via direct oral challenge
Source: PLoS One. 2025 Sep 5;20(9):e0330724. doi: 10.1371/journal.pone.0330724 (PMC12412947; doi:10.1371/journal.pone.0330724)

| protocol |
| --- |
| 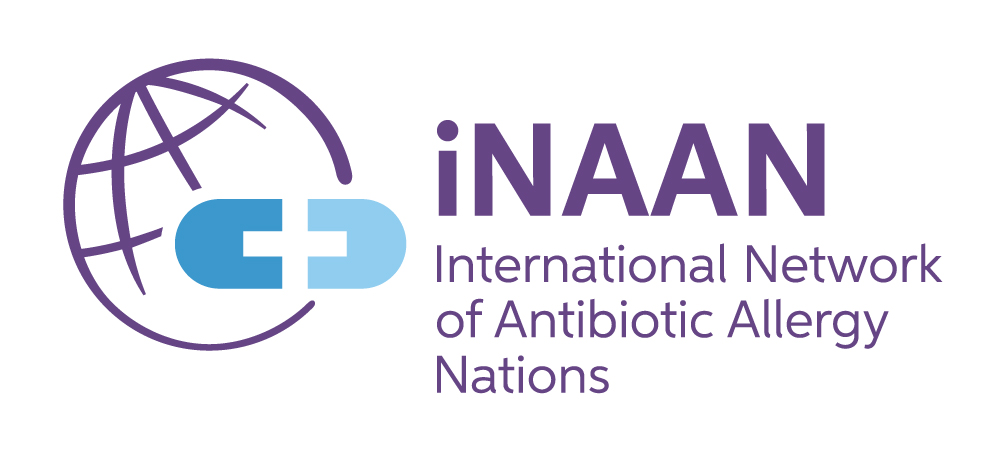 |
| International Network of Antibiotic Allergy Nations (iNAAN) |
| Protocol Number: 78719  Version: 3.2  Date: 20 September 2024 |
|  |
| **Author/s:**  Prof Jason Trubiano  **Sponsor/s:**  Austin Health |
| **CONFIDENTIAL**  This document is confidential and the property of Austin Health. No part of it may be transmitted, reproduced, published, or used without prior written authorization from the institution.  **Statement of Compliance**  This document is a protocol for a research project. This study will be conducted in compliance with all stipulation of this protocol, the conditions of the ethics committee approval, the NHMRC National Statement on ethical Conduct in Human Research (2007) and the Note for Guidance on Good Clinical Practice (CPMP/ICH-135/95). |

| **Version** | **Date** | **Summary of changes** |
| --- | --- | --- |
| 1.1 | 13 December 2021 | First version approved by Austin Health HREC |
| 2.0 | 25 May 2022 | BioGrid Australia contracted for database hosting and management |
| 2.1 | 28 September 2022 | 1. Removal of participating site list from protocol to allow for flexibility in adding and removing sites throughout project lifespan 2. Inclusion criteria amended |
| 2.2 | 10 February 2023 | Addition of waiver of consent wording |
| 2.3 | 28 March 2023 | Addition of wording to stipulate that the iNAAN project team and Austin Health staff will not have access to data from any other site in an identifiable format. Addition of wording to stipulate that the iNAAN project is now an international study. |
| 2.4 | 14 July 2023 | Addition of implementation strategy and secondary implementation study outcomes. Revision of the statistical analysis and implementation evaluation to incorporate implementation strategy. |
| 3.0 | 1 Sept 2023 | 1. Change name of study to: ‘International Network of Antibiotic Allergy Nations (iNAAN)’ to reflect international health service participation. 2. Inclusion of further iNAAN Steering Committee details 3. Inclusion of a publication and authorship guidelines |
| 3.1 | 22 January 2024 | 1. Inclusion of further details and process pertaining to de-identified data access by researchers, governing bodies and other organisations 2. Inclusion of process for Project Requests by current investigators to propose and conduct sub-studies using iNAAN data. |
| 3.2 | 20 September 2024 | 1) Refined definition of primary and secondary effectiveness implementation outcomes to provide clarity  2) Included wording to support addition of appendices to describe sub-studies and/or populations, regions, or settings with particular legislative, logistical, governance or ethical considerations not covered in the main protocol.  3) Refined statistical analysis plan and implementation evaluation details |

**Document History**

Contents

[1. Glossary of Definitions and Abbreviations 5](#_Toc144474966)

[2. Introduction and Background Information 6](#_Toc144474967)

[a. Lay Summary 6](#_Toc144474968)

[b. Background information 6](#_Toc144474969)

[3. Study Objectives 6](#_Toc144474970)

[a. Hypothesis 6](#_Toc144474971)

[b. Study Aims 6](#_Toc144474972)

[**4.** **Outcome Measures** 7](#_Toc144474973)

[**5.** **Study Design** 7](#_Toc144474974)

[a. Study design 7](#_Toc144474975)

[b. Study population 8](#_Toc144474976)

[c. Patient identification 8](#_Toc144474977)

[d. Waiver of consent 8](#_Toc144474978)

[e. Allergy assessment and oral challenge 9](#_Toc144474979)

[f. Implementation Strategy 9](#_Toc144474980)

[g. Data collection 9](#_Toc144474981)

[**6.** **Statistical Methods** 10](#_Toc144474982)

[a. Sample size 10](#_Toc144474983)

[b. Statistical Methods To Be Undertaken 11](#_Toc144474984)

[c. Implementation Evaluation 11](#_Toc144474985)

[**7.** **Data Security & Handling** 11](#_Toc144474986)

[a. Database Hosting and Management 11](#_Toc144474987)

[b. Data Storage 12](#_Toc144474988)

[c. Confidentiality and Security 12](#_Toc144474989)

[**8.** **Data Access and Database Reporting** 12](#_Toc144474990)

[a. Access for Contributing Sites 12](#_Toc144474991)

[b. Access for Reporting 13](#_Toc144474992)

[c. Access for Research 13](#_Toc144474993)

[d. Database Reporting 13](#_Toc144474994)

[e. Individual Reports 13](#_Toc144474995)

[f. Publication and Authorship Guidelines 14](#_Toc144474996)

[**9.** **Governance** 14](#_Toc144474997)

[a. iNAAN Project Team 14](#_Toc144474998)

[b. iNAAN Steering Committee 14](#_Toc144474999)

[**10.** **References** 16](#_Toc144475000)

| **STUDY SYNOPSIS** |  |
| --- | --- |

| **Title:** | International Network of Antibiotic Allergy Nations |
| --- | --- |
| **Short Title:** | iNAAN |
| **Design:** | Prospective cohort and implementation study and audit of a clinical health services program |
| **Coordinating Study Centre:** | Austin Health |
| **Study Questions:** | 1. Can targeted discovery of hospitalised patients with a penicillin antibiotic allergy label (AAL) improve access to antibiotic allergy testing (AAT) and appropriate penicillin allergy delabelling? 2. Can an oral challenge and delabelling program increase the use of appropriate antibiotics and decrease the use of expensive restricted antibiotics? 3. Can an oral challenge and delabelling program lead to reduced antimicrobial resistance? 4. Is it cost effective to implement oral challenge and delabelling? 5. Can an audit and feedback implementation strategy, delivered digitally, for a validated point-of-care penicillin allergy toolkit, improve uptake? |
| **Study Objective:** | To audit the outcomes of implementation of direct oral penicillin challenge and delabelling at participating sites |
| **Primary Objective:** | To establish an international database for direct oral penicillin challenge and delabelling data |
| **Secondary Objectives** | 1. To determine the proportion of patients with a low-risk penicillin AAL delabelled following direct oral challenge (DOC) 2. To determine the proportion of patients that utilize a penicillin pre and post allergy assessment +/- testing 3. To determine the proportion of patients that utilize an appropriate antibiotic pre and post allergy assessment +/- testing 4. To determine the proportion of patients that utilize a restricted antibiotic pre and post allergy assessment +/- testing 5. To determine hospital length of stay in patients that undergo penicillin allergy testing 6. To determine the cost-effectiveness of oral penicillin challenge 7. To develop recommendations for national and international scaling and implementation across health systems (based on feasibility, costs, barriers and facilitators) 8. To assess the efficacy of the implementation strategy, delivered digitally:    - Acceptability; Adoption; Fidelity; Feasibility, Implementation Cost; Sustainability; Adherence to clinical standards; changes in health policy |
| **Inclusion Criteria:** | Patients ≥ 18 years of age reporting a penicillin allergy*  **Please note: sites may seek separate ethics approval to include paediatric patients aged <18 years.* |
| **Exclusion Criteria:** | None |
| **Number of Planned Subjects:** | Flexible |
| **Investigational product:** | N/A |
| **Statistical Methods:** | Basic statistical methods & implementation evaluation |

## **Glossary of Definitions and Abbreviations**

| **Abbreviation** | **Description or definition** |
| --- | --- |
| AAL | Antibiotic allergy label |
| AAT | Antibiotic allergy testing |
| ADR | Adverse drug reaction |
| BioGrid | BioGrid Australia Ltd |
| Course | Greater than one dose of prescribed systemic antibiotic |
| Delabelling | The removal of a patient-reported antibiotic allergy label on their medical record – either via direct de-labelling (medical reconciliation/assessment; no testing required) or test dose procedure (direct oral challenge) |
| DOC | Direct oral challenge: administration of an oral test dose of a drug |
| IBM | International Business Machines Corporation |
| ICER | Incremental cost effectiveness ratio |
| IPTWS | Inverse probability treatment weighted propensity score |
| LOS | Length of stay |
| iNAAN | International Network of Antibiotic Allergy Nations |
| Narrow spectrum beta-lactam | “Penicillin” as defined here, cephalexin, cefaclor, cefazolin |
| PEN-FAST | Penicillin Allergy Clinical Decision Rule (1) |
| Penicillin | Penicillin VK, benzylpenicillin, benzathine penicillin, amoxicillin, ampicillin, amoxicillin clavulanate, flucloxacillin, dicloxacillin, penicillin “unspecified” |
| REDCap | Research Electronic Data Capture |
| Restricted antimicrobial agents | Cefepime, ceftazidime, ceftriaxone, ciprofloxacin, clindamycin, meropenem, moxifloxacin, piperacillin/tazobactam, teicoplanin, tobramycin and vancomycin |
| SAS | Statistical software |
| SCAR | Severe cutaneous adverse reaction |
| SSL | Secure Socket Layer |

##

## **Introduction and Background Information**

### Lay Summary

More than two million Australians report an antibiotic or penicillin allergy. Patients’ reports are not always accurate, which may lead to them receiving second-line antibiotic therapies. It has been found that 85% of reported allergies are false and can be removed by testing: in fact, more than 95% of low-risk penicillin allergies. The ***International Network of Antibiotic Allergy Nations*** is a health services program that seeks to improve access to direct oral penicillin challenge for Australian and international patients. This will reduce the burden and cost of antibiotic allergies in healthcare. At present, many of the patients carrying an antibiotic allergy label remain undiscovered with 50% of these patients not having access to vital testing. We seek to prospectively audit this clinical program and implementation to assess safety and the impact on reducing the burden of penicillin allergy in hospitalized patients and the subsequent effects on antibiotic prescribing and patient outcomes.

### Background information

Patient-reported antibiotic allergy labels (AALs), most commonly penicillin, are a major public health concern with an estimated 18% of all hospitalised Australians reporting an antibiotic allergy.(1) The prevalence of AALs is significantly higher in our most vulnerable patients. Up to 1 in 4 patients admitted to hospital with a cancer diagnosis have an AAL.(2)

Additionally, patients with an AAL are more likely to receive an inappropriate antibiotic, suffer a hospital-associated adverse event and acquire a multidrug resistant organism.(3-6) In Australia AALs are associated with increased length-of-stay (LOS), higher readmission rates, increased hospital costs and mortality rates.(2, 3, 7) Internationally, the literature shows an adverse drug reaction (ADR) is associated with an increased LOS of 1.0 - 2.2 days and hospital costs of USD$3200 per patient.(8-11)

Our pilot Penicillin Allergy Delabelling Program demonstrated a 3-day reduction in LOS for those who received an oral penicillin rechallenge (2). Utilising published LOS costing data, this would result in a saving of AUD$3558 per/patient.(12) Prior cohort studies have demonstrated that antibiotic allergy testing (AAT) can successfully remove 83% of all penicillin AALs and improve appropriate antibiotic prescribing.(13)

Previously, the implementation of a penicillin allergy toolkit (antibiotic allergy assessment tool and oral penicillin challenge program) in a multi-centre study demonstrated that 97% of patients were negative on testing, and that inpatient direct oral challenge improved prescribing and was cost-effective.(14) However, a national or international implementation strategy for an effective penicillin allergy toolkit has not been previously published.

## **Study Objectives**

### Hypothesis

iNAAN will enable the implementation of a locally adaptable penicillin allergy toolkit throughout Australian and international hospitals, increasing service capacity to discover and delabel more patients reporting a penicillin AAL. Greater than 90% of discovered low-risk penicillin AALs are hypothesized to be delabelled following DOC, subsequently improving antibiotic utilization and health outcomes and reducing hospital costs. That the penicillin allergy toolkit can be implemented via audit and feedback implementation strategy, delivered digitally.

### Study Aims

Co-Primary^1^: To audit the outcomes of implementation of direct oral penicillin challenge and delabelling at Australian and international participating sites.

Co-Primary^2^: To assess the effects of a strategy to implement a penicillin allergy toolkit on implementation outcomes,

### **Outcome Measures**

To audit the implementation strategy and impacts of the penicillin allergy toolkit at Australian and international participating hospital sites with respect to the following domains:

***Primary Effectiveness Outcome:***

To determine the proportion (n, %) of patients discovered with a low-risk penicillin allergy that are delabelled following DOC

***Secondary Effectiveness Outcomes:***

1. a) To determine the proportion (n, %) of patients that utilize a penicillin pre-testing (at index admission) vs post-testing (up to 90 days post-discharge)

b) Penicillin utilization within 90 days of penicillin allergy assessment in patients who undergo testing compared with patients that do not undergo testing

1. a) To determine the proportion (n, %) of patients that utilize a narrow-spectrum beta-lactam pre-testing (at index admission) vs post-testing (up to 90 days post-discharge)

b) Narrow-spectrum beta-lactam utilization within 90 days of penicillin allergy assessment in patients who undergo testing compared with patients that do not undergo testing

1. a) To determine the proportion (n, %) of patients that utilize an appropriate antibiotic pre-testing (at index admission) vs post-testing (up to 90 days post-discharge)

b) Appropriate antibiotic utilization within 90 days of penicillin allergy assessment in patients who undergo testing compared with patients that do not undergo testing

1. a) To determine the proportion (n, %) of patients that utilize a restricted antibiotic pre-testing (at index admission) vs post-testing (up to 90 days post-discharge)

b) Restricted antibiotic utilization within 90 days of penicillin allergy assessment in patients who undergo testing compared with patients that do not undergo testing

1. To compare the median hospital LOS (days, IQR) for delabelled patients with a low-risk AAL against patients with a high-risk AAL that were not delabelled
2. To determine the cost-effectiveness of the program model and implementation strategy
3. To develop recommendations for national scaling and implementation of the program across the health system (based on feasibility, costs, barriers and facilitators)

***Primary Implementation Outcome:***

To determine the adoption (i.e. uptake) of the intervention by clinicians within participating sites (n, %) and hospitals across Australia

***Secondary Implementation Outcomes:***

1. To determine the perception amongst participating hospital clinicians that the penicillin allergy toolkit is agreeable (i.e. acceptability)
2. To determine the fidelity (n, %) of the intervention (i.e. adherence) to the audited hospital site-specific protocol
3. To determine the proportion of patients (n, %) who meet the NSQHS standards for antibiotic allergy assessment (i.e. adherence), stratified for site.
4. To determine the changes in health policy

# **Study Design**

### Study design

Audit of antibiotic allergy assessment and direct oral penicillin challenge: prospective multicenter, international cohort study with audit and feedback implementation strategy.

### Study population

1. Patients ≥ 18 years of age reporting a penicillin allergy*

**Please note: sites may seek separate ethics approval to include paediatric patients aged <18 years.*

### Patient identification

Eligible inpatients will be identified by study investigators or their delegates: in most cases by Antimicrobial Stewardship (infectious diseases physicians or pharmacists) or allergy service staff. Participants will include all eligible inpatients that undergo allergy assessment. Patients reporting a low-risk penicillin allergy will additionally receive a direct oral penicillin challenge. Participants may only be included once.

### Waiver of consent

Penicillin allergy assessment and direct oral challenge are considered routine clinical practice in the hospital setting as per the National Safety and Quality Health Service Standards. A waiver of consent has been sought as the proposed study is a low-risk audit of routine clinical practice with an adequate plan to protect the confidentiality of data. Clinical consent is still required per routine practice for the direct oral penicillin challenge procedure.

As per section 2.3.10 of the National Statement we are requesting a prospective waiver of consent to access 4,000 records over 10 years due to the following reasons:

- it is impracticable to obtain consent due to the number of records to be accessed. This study will collect 4,000 records from 27 sites around Australia and internationally. Please note the number of sites may increase as we approach new investigators to participate in the project.
- the benefits from the research justify any risks of harm associated with not seeking consent because this project will encompass data from around Australia and internationally, which we hope will allow us to assess the real impact on patients and their outcomes within the National Antibiotic Allergy Network. This information can then be used to aid in the development of recommendations for national and international scaling and implementation across the health system.
- there is sufficient protection of patient privacy. All data will be stored, as per section 7 of this document. The iNAAN project team and Austin Health staff will not have access to data from any other participating health service in an identifiable format. When the data is shared via publication or presentation it will be deidentified and aggregated to ensure that no singular individual can be identified.
- there is an adequate plan to protect the confidentiality of data, as per section 7 of this document.

Additionally, the project also meets the following voluntary requirements:

involvement in the research carries no more than low risk to participants.

there is no known or likely reason for thinking that participants would not have consented if they had been asked

in case the results have significance for the participants’ welfare there is, where practicable, a plan for making information arising from the research available to them (for example, via a disease-specific website or regional news media). As there is no patient follow-up as part of this project, patients will not be individually informed of the outcome of this project. However, we do intend to publish the results to the public.

there is no possibility of commercial exploitation of derivatives of the data or tissue will not deprive the participants of any financial benefits to which they would be entitled.

the waiver is not prohibited by State, federal, or international law.

### Allergy assessment and oral challenge

Antibiotic allergy assessment will be conducted with the Antibiotic Allergy Assessment Tool (Appendix 1) (15) OR PEN-FAST (Appendix 2) (16), as adapted by each clinical site. The oral challenge protocol will be conducted with per the guideline in Appendix 3 or per the site’s hospital guideline.

### Implementation Strategy

**The implementation strategy** will be based on published advice on the delivery of the audit and feedback strategy and effectiveness-implementation hybrid design **(17).** The strategy, outlined in the implementation manual, will gather quantitative and qualitative data to measure implementation outcomes and is underpinned by the Framework of Implementability (19). The AACTT (Action, Actor, Context, Target, Timeframe) framework will be to used to identify behaviors key to audit and feedback implementation strategy (18). The study implementation plan is outlined below:

**Primary Implementation Strategy**: Digitally delivered audit and feedback (de-identified) to clinicians and health services (from participating sites, at least 6 months following post site approval).

- Delivered via BioGrid digital reporting (clinician & hospital): Bimonthly data on assessment, delabeling, oral challenges, antibiotic prescribing, serious adverse events (SAEs), mortality and antimicrobial resistance – across all implementation outcomes.
- **Implementation Comparison**: No audit and feedback to clinicians. After administration of the ‘toolkit’ at each site, no audit and feedback will be provided for the first 3-6 months of the implementation.

### Data collection

Data will be collected by study investigators or their delegates and stored in a secure REDcap database hosted by BioGrid. BioGrid is contracted by Austin Health to host and manage the iNAAN REDCap database and associated API software as well as provide dynamic reporting for the iNAAN participating sites.

**Data can be entered into iNAAN REDCap database using either the:**

- 1. National Antibiotic Allergy Network smartphone app, a tool designed specifically for the collection of data and secure upload to the REDCap database, which will be utilised by most investigators (see Smartphone App Front-End section below, Appendix 4) or
  2. REDCap web portal directly, which will be used by the study investigators and site administrators.

**Data sources will include:**

1. Patients at the time of assessment and oral challenge
2. NAPS (National Antibiotic Prescribing Survey) data
3. Hospital electronic medical records and business intelligence unit (for costing data, baseline penicillin allergy denominator data)

**Data to be collected includes (see Appendix 5 for complete REDCap CRF):**

*Minimum Data (all sites)*

1. Patient demographics and comorbidities
2. Antibiotic Allergy Assessment and PEN-FAST assessment
3. Penicillin oral challenge data (if performed)

*Additional data (optional for sites)*

1. Admission details (including hospital metrics)
   1. Date of admission/discharge
   2. Diagnoses at admission/discharge
   3. ICU admission
   4. Readmission(s) within 30 days
2. Antibiotic usage pre/post-testing
3. For each antibiotic: drug, dose, duration, frequency, route
4. Appropriateness score as per published definition (19)
5. Preferred antibiotic per hospital guidelines (per therapeutic guidelines if not applicable) (20))
6. Narrow spectrum beta-lactam, penicillin or restricted antibiotic status
7. Cost per antibiotic course (per pharmacy dispensing)
8. Exclude topical and inhaled antibiotics
9. Exclude antifungal and antiviral prophylaxis agents
10. Antibiotic adverse events
    1. Agent, reaction phenotype, severity, grade, outcome

**Smartphone App Front-End (see Appendix 4)**

A smartphone app has been built to permit data collection from study assessors, automatic phenotype and risk assessment using decision support logic, and secure upload of this data to the iNAANREDCap database hosted by BioGrid.

The smartphone app will be provided to users on their own personal smartphone or hospital devices, as required. Access to the smartphone app will be protected via individual username/password authentication.

- 1. Authenticated smartphone app users have write-only access and can upload data to REDCap to facilitate data collection, but cannot access previous patient data from REDCap. Users can only upload data to clinical sites that they are associated with and are unable to upload data from other sites.
  2. All data are transmitted to REDCap securely, and once submitted, no patient data are stored on the user’s device.
  3. Additional information
     1. The smartphone app IP is owned by the chief investigator
     2. The smartphone app does not provide advice regarding specific allergy testing, nor does it provide instruction on how to perform allergy testing

**Implementation Outcomes Data**

This data is summarized below but is readily available from the audit aforementioned data collection and methods.

| **Implementation Outcome** | **Data** | **Data Source** |
| --- | --- | --- |
| Acceptability | Survey (end-users) – **Appendix 6** | REDCap database |
| Adoption | % Australian sites with uptake  % Clinicians within sites utilising | REDCap database |
| Fidelity | Adherence to protocol | REDCap database |
| Implementation Cost | Administrative data | Hospital Costings |
| Sustainability | Assessment & Oral Challenge data | REDCap database |
| Adherence - clinical standards | No. patients meeting NSQHS | REDCap database |

### Appendices for Sub-studies and Special Populations

Throughout the conduct of this audit and feedback implementation strategy study, sub-studies may be commenced, and will be fully described in sub-study appendices, to be read in conjunction with the main study protocol.

Appendices for sub-studies may include the following elements (as applicable):

1. Background and rationale
2. Sub-study governance
3. Sub-study objectives
4. Sub-study design
5. Sub-study population
6. Sub-study conduct
7. Sub-study data collection, management, security and analysis
8. Sub-study Ethical considerations
9. Consent where study conduct does not fall within the waiver of consent granted for the main study

Appendices may also be included to describe the application of the study in populations (eg paediatric) or regions (national, international) or settings with particular legislative, logistical, governance or ethical considerations not covered in the main protocol

# **Statistical Methods**

### Sample size

The estimated sample size across all sites over 10 years is 4000 (400 per year), however case capture is competitive and will not be capped at this number. The estimate is based on a previous study which performed 400 oral challenges at two sites over a 2-year period.

### Statistical Methods To Be Undertaken

An analysis of the impacts of the program on named objectives relating to (i) de-labelling, (ii) antibiotic utilization and (iii) model costs will be performed.

Baseline characteristics will be presented using median with interquartile range and frequency with percentage. Primary outcome (number of participants delabelled) will be presented as count and percentage of all participants who received oral challenge with 95% confidence intervals.

Allergy utilization will be coded as number of participants receiving at least one dose of antibiotic of interest. Logistic regression will be used to compare those delabelled with those not undergoing oral challenge, adjusted for baseline (pre-assessment/testing) antibiotic use and other participants’ characteristics.

Additionally a trial emulation causative analysis will be performed to evaluate the impact of oral challenge on prescribing. Target trial will be designed prior the study completion (with details of inclusion/exclusion criteria, intervention (oral challenge) and control arm (assessment only) and estimand of interest). Logistic regression will be used to calculate probability of treatment (oral challenge) and inverse probability of treatment weighting will be used to evaluate the effect of oral challenge on antibiotic prescribing. This analysis will be performed separately for each antibiotic group. Detailed analysis plan and protocol of target trial will be prepared separately prior to study completion.

The following is specific to the economic evaluation. The mean cost difference will be determined between the retrospective group and the intervention Group I, as well as between the retrospective group and the intervention Group II, using independent t-tests to report significance. Incremental cost-effectiveness ratios (ICERs) will be determined for the retrospective group and the intervention Group I, as well as for the retrospective group and the intervention Group II, based on a ratio between the incremental difference in cost per patient de-labelled. Confidence intervals around the individual ICERs will be calculated using bootstrapping methods (5,000 repetitions), the difference in the number of patients de-labelled and total cost. Confidence ellipses will be generated via the individual ICERs.

### Implementation Evaluation

A mixed methods evaluation, balancing in-depth qualitative data with quantitative data from participating sites (some which is already collected as part of the audit framework) will be utilised. Qualitative data, obtained via semi-structured focus groups of open-ended survey questions, will be thematically analysed to identify barriers and facilitators for adoption. This analysis will be examined using an appropriate theoretical framework (e.g., Behaviour Change Wheel (19) and the Framework of Implementability (19), to refine the intervention and/or strategy.

Effect of the implementation strategy (ie,. audit and feedback) on each of the implementation outcomes (collected on a bimonthly or six-monthly basis) will be evaluated using interrupted time series (ordinary least square methods). This method enables the comparison to the 3-6 months without the implementation strategy as well as comparison over time, while taking into account the autocorrelation and seasonal effects. The iNAAN data will be used to identify new areas for action and to monitor Program impact on named outcomes.

# **Data Security & Handling**

### Database Hosting and Management

The iNAAN REDCap database will be managed by, and hosted within the BioGrid environment behind internal security firewalls at Melbourne Health with access only being staff employed by BioGrid and authenticated users. Austin Health has contracted BioGrid to host, manage and provide reporting for the iNAAN on its behalf.

BioGrid is a not for profit company limited by guarantee. Its members are health services, universities and research institutes that utilise this trusted virtual real-time data sharing platform to conduct collaborative multi-site research. BioGrid specialises in collection and real-time linkage of hospital-based and managed clinical treatment outcome, genomic, biospecimen, imaging and patient administration system data. BioGrid is viewed by the health and medical research sector as a trusted independent neutral provider of research data management, linkage and curation services (i.e. independent of government and industry, owned by research sector organisations).

BioGrid utilises monitoring software to provide database management and ongoing maintenance services. Regular monthly security patching is applied to BioGrid servers and applications and network and server performance is monitored daily to ensure optimum performance for users. Database management includes creating and managing authorised users who will have approved administration access to the iNAAN REDCap database.

BioGrid has extensive experience managing servers, databases and the flow of data from multiple institutions. BioGrid currently hosts several source databases providing hosting as well as data management and reporting services. These hosted databases include the Australian Psoriasis Registry (for the Skin Health Institute), Centre for the Analysis of Rare Tumours (Global Consumer Data Collection), Advanced Metastatic Colorectal Cancer (for Prince of Wales Hospital, Hong Kong), HER2 Breast Cancer (for Canberra Hospital, ACT), the Australian Rare Cancer Portal (for Australian Genomic Cancer Medicine Centre now known as Omico) and the Australian and New Zealand Thoracic Clinical Quality Registry (for Australian and New Zealand Society of Cardiac and Thoracic Surgeons).

The iNAAN REDCap database will be routinely backed up and encrypted on a secure server (according to ISO27001 standards) to minimise the event of unauthorised access to the database. All server and database backups are provided by Melbourne Health IT as part of the hosting service agreement with BioGrid. BioGrid monitors database backups daily and manages any backup failures and restorations with Melbourne Health. All source data is stored on BioGrid secure servers within the Melbourne Health IT environment. The iNAAN Study is expected to be ongoing and data will be retained indefinitely.

### Data Storage

Electronic patient data will be stored indefinitely in the iNAAN REDCap database hosted by BioGrid Australia (refer to Section 7a. Database Hosting and Management section for more information). Data will only be accessible by approved study investigators and their delegates (refer to Section 7c. Confidentiality and Security and section 8. Data Access and Database Reporting for more information).

### Confidentiality and Security

Records will be kept on a password protected computer within a password protected Redcap database and will only be accessible by named study investigators. The iNAAN project team will have access to all data, however this will not be in an identifiable format. Individual site investigators will have access to their own site data in an identifiable format. Sites will only have access to site specific data via REDcap export. Data for all sites will be stored in the iNAAN REDCap database hosted at BioGrid. As BioGrid’s core business is accessing, ingesting, linking and curating health and medical data on behalf of its’ members, collaborators and clients to enable research activities, all BioGrid staff undergo data and privacy confidentiality as well as data and information security training and monitoring.

The iNAAN REDCap database hosted at BioGrid will be secured through IBM Security Access Manager and WebSEAL Authenticating Reverse Proxy. It provides SSL (Secure Socket Layer) communications with fine grained access controls. This will facilitate securely encrypted connections to the iNAAN REDCap web application hosted at BioGrid. SSL ensures that a private key on the server encrypts all data before it is sent to the user where it is decrypted by a public key. This ensures the data is not compromised in transit. For additional security, two-factor authentication will be activated on the iNAAN REDCap database. All users of the iNAAN REDCap database will login via a secure username and password controlled by administrators of the system.

# **Data Access and Database Reporting**

### Access for Contributing Sites

Contributing clinicians will receive access to their own data in both raw record-level format and aggregated reporting via the BioGrid platform. This is to enable participating clinicians to review their own data in relation to its primary purpose for collection, i.e. to improve outcomes for their patients. The raw data available to the contributing clinicians will be via the BioGrid platform in a de-identified form to protect patient privacy. Contributing clinicians will be able to re-identify their patients by referencing the database ID for each participating patient.

Whilst individual clinicians will be able to view and access their own data, they will only be able to edit data using tracked changes. Random site audits for accuracy and completion is planned and the database will bet set up such that all data can be recorded and audited including any changes to the data. Training on data entry will be provided by the iNAAN team and supported by the PI at each hospital site.

### Access for Reporting

BioGrid staff will access the iNAAN REDCap database to prepare periodic static monthly and annual data reports as required by the iNAAN Project Team, contributing clinicians and other key stakeholders. BioGrid will provide the iNAAN Project Team with de-identified aggregated reporting data that contains information from across the whole database for the purposes of quality assurance.

### Access for Research

The iNAAN REDCap database will be available to access de-identified data for ethically approved research studies via the BioGrid data platform. External researchers, including governing bodies and other organisations, may apply for access to the iNAAN REDCap database via the online BioGrid Access Request System. The application process includes:

- Completion of the BioGrid Australia online application to access de-identified iNAAN data providing scientific relevance and significance for the research study of interest.
- Agreement by all researchers to [BioGrid Terms and Conditions of Data Access](https://www.biogrid.org.au/page/58/terms-and-conditions). These terms and conditions align with the BioGrid Collaboration Agreement and define the researcher’s responsibilities with regards to data accessed via BioGrid.
- Review and approval of the researchers’ application for access to the iNAAN dataset by the iNAAN Steering Committee. The iNAAN Project Team and Steering Committee retains control over to whom and for what purpose the iNAAN data is accessed and is well placed to advise researchers on the appropriateness of the iNAAN’s data for proposed projects.
- Approval from a properly constituted Human Research Ethics Committee for the researchers’ project if data request or objectives are outside the current approved protocol.
- Completion of a data or material transfer agreement established between Austin Health and the requesting researchers, governing body or organization, including the agreed frequency and duration of access to iNAAN data.

Access to iNAAN REDCap data will only be provided to researchers by BioGrid if the iNAAN Project Team and Steering Committee provide approval and the proposed research project has received appropriate ethical approval.

Access to de-identified iNAAN REDCap data can be shared with rekevant government health bodies for consumer facing reports using the same Data Request process outlined above and an appropriate data sharing agreement in situ.

Any projects accessing data from the iNAAN REDCap database will need to acknowledge the contribution of the Registry.

### Database Reporting

To facilitate the provision of periodic static and dynamic (refreshed nightly with new and/or edited data) reporting utilising SAS Visual Analytics, the iNAAN REDCap database will be linked to the BioGrid platform. This will enable the use of SAS Enterprise Guide statistical software to ingest and standardise the data for creating statistical outputs for reporting as well as making the data research-ready for linkage to other external datasets, such as the AIHW National Death Index, if/when required.

The creation and maintenance of periodic static monthly and annual data reporting will enable the preparation of monthly and annual reports to participating clinicians and other key stakeholders.

All data related to participant information will be presented in aggregate summary form and will not be identifiable to protect participant privacy. No data from patients who have declined to participate will be included in any data analysis or reporting.

Information related to individual clinicians will also be reported in aggregate summary form and will not be identifiable to protect clinician privacy. Contributing hospitals will be listed in reports but data related to the procedures and outcomes from those sites will not be reported publicly or to other parties without prior consent.

### Individual Reports

Contributing clinicians and hospitals will receive access to their own data in both raw format and aggregated reporting via the BioGrid platform. These reports can be tailored for participating sites as required to be meaningful and useful.

Dynamic reports can be made available on mobile devices such as tablets and phones for each participating clinicians, enabling them to view their data in a timely and dynamic manner. This encourages participation and when all site data is combined, can provide dynamic reporting across the entire dataset for review by the iNAAN Project Team. Dynamic reports may reflect the periodic reporting, and/or other items of interest. These reports will form part of the audit and feedback implementation strategy.

### Publication and Authorship Guidelines

Criteria for authorship for manuscripts arising will be those of the International Committee of Medical Journal Editors (ICMJE). Specifically, authors should satisfy:

- Substantial contributions to the conception or design of the work; or the acquisition, analysis or interpretation of data for the work; AND
- Drafting the work or revising it critically for important intellectual content; AND
- Final approval of the version to published; AND
- Agreement to be accountable for all aspects of the work in ensuring that questions related to the accuracy or integrity of any part of the work are appropriately investigated and resolved.

The author by-line will include: the iNAAN study group. An updated database of members of the broader iNAAN study group will be maintained and where appropriate, the list of members will be provided as an appendix to submitted manuscripts. The author by-line will include the key participating regional research networks. All sources of funding will be acknowledged.

Abstracts and manuscripts (including pre-prints) reporting results or other data from the iNAAN study must obtain written approval by the iNAAN Steering Committee prior to submission. Prior to public release, interim or final results will not be publicised, including in oral presentations without permission from the iNAAN Steering Committee.

All participating investigators may propose sub-studies to lead with utilisation of the iNAAN data for consideration of publication. The application process includes:

- Submission of a Project Request Form (Appendix 7) to the iNAAN Steering Committee detailing the objectives, scientific relevance and clinical significance of the proposed sub-study.
- Investigator project requests will be reviewed by the iNAAN Steering Committee and endorsed by majority.
- Authorship of sub-studies will also follow ICMJE guidelines.

# **Governance**

### iNAAN Project Team

The iNAAN Project Team has been established to provide day-to-day management of the project. The group is led by the Coordinating Principal Investigator, Prof Jason Trubiano and members will include associate investigators and key research staff from the lead site, Austin Health. The group will meet regularly to discuss the ongoing management of the project. Responsibilities include:

- Managing ethics submissions and reporting requirements
- Assisting participating sites with local governance processes
- Distributing project documentation and communicating updates to key participating site personnel
- Discussing issues or barriers to the project’s success
- Liaising with BioGrid and other internal or external stakeholders regarding data management, storage and analysis
- Ensuring the project is conducted in accordance with ICH-GCP guidelines

### iNAAN Steering Committee

The iNAAN Steering Committee is comprised of 5-7 members and has been established to provide practical and strategic advice regarding the conduct and progress of the project. Representatives from each discipline have been invited to participate as members and contribute to the project design, share expertise and local antibiotic allergy assessment resources and guide the project from inception. A representative (outside of the Chair and Project manager) may serve as a steering committee member two years before nominations for a new representative within discipline will be sought. A previously elected steering committee member may seek re-election.

Members include:

- Chair
- Project manager
- Allergist/Immunologist
- Infectious Diseases Physician
- Nurse or Nurse Practitioner
- Pharmacist
- Consumer
- International representative

The iNAAN Steering Committee will be chaired by the Chief Investigator and meet regularly. Responsibilities include:

- Contributing to the project design
- Reviewing the progress of participating site activation and commencement
- Providing recommendations to the iNAAN Project Team
- Considering the strategic direction of the project
- Contributing to funding strategy
- Consideration of requests to amend study documents or the iNAAN REDCap database
- Consideration of project development requests from sites
- Approval of manuscripts reporting results utilising iNAAN data

# **References**

1. Trubiano JA, Chen C, Cheng AC, Grayson ML, Slavin MA, Thursky KA, et al. Antimicrobial allergy 'labels' drive inappropriate antimicrobial prescribing: lessons for stewardship. The Journal of antimicrobial chemotherapy. 2016;71(6):1715-22.

2. Trubiano JA, Leung VK, Chu MY, Worth LJ, Slavin MA, Thursky KA. The impact of antimicrobial allergy labels on antimicrobial usage in cancer patients. Antimicrob Resist Infect Control. 2015;4:23.

3. Macy E, Contreras R. Health care use and serious infection prevalence associated with penicillin "allergy" in hospitalized patients: A cohort study. J Allergy Clin Immunol. 2014;133(3):790-6.

4. MacFadden DR, LaDelfa A, Leen J, Gold WL, Daneman N, Weber E, et al. Impact of Reported Beta-Lactam Allergy on Inpatient Outcomes: A Multicenter Prospective Cohort Study. Clinical infectious diseases : an official publication of the Infectious Diseases Society of America. 2016;63(7):904-10.

5. Blumenthal KG, Lu N, Zhang Y, Li Y, Walensky RP, Choi HK. Risk of meticillin resistant Staphylococcus aureus and Clostridium difficile in patients with a documented penicillin allergy: population based matched cohort study. Bmj. 2018;361:k2400.

6. Blumenthal KG, Ryan EE, Li Y, Lee H, Kuhlen JL, Shenoy ES. The Impact of a Reported Penicillin Allergy on Surgical Site Infection Risk. Clin Infect Dis. 2018;66(3):329-36.

7. Knezevic B, Sprigg D, Seet J, Trevenen M, Trubiano J, Smith W, et al. The revolving door: antibiotic allergy labelling in a tertiary care centre. Intern Med J. 2016;46(11):1276-83.

8. Bates DW, Spell N, Cullen DJ, Burdick E, Laird N, Petersen LA, et al. The costs of adverse drug events in hospitalized patients. Adverse Drug Events Prevention Study Group. Jama. 1997;277(4):307-11.

9. Charneski L, Deshpande G, Smith SW. Impact of an antimicrobial allergy label in the medical record on clinical outcomes in hospitalized patients. Pharmacotherapy. 2011;31(8):742-7.

10. Sousa-Pinto B, Cardoso-Fernandes A, Araujo L, Fonseca JA, Freitas A, Delgado L. Clinical and economic burden of hospitalizations with registration of penicillin allergy. Annals of allergy, asthma & immunology : official publication of the American College of Allergy, Asthma, & Immunology. 2018;120(2):190-4 e2.

11. Wu JH, Langford BJ, Schwartz KL, Zvonar R, Raybardhan S, Leung V, et al. Potential Negative Effects of Antimicrobial Allergy Labelling on Patient Care: A Systematic Review. Can J Hosp Pharm. 2018;71(1):29-35.

12. Morello RT, Barker AL, Watts JJ, Haines T, Zavarsek SS, Hill KD, et al. The extra resource burden of in-hospital falls: a cost of falls study. The Medical journal of Australia. 2015;203(9):367.

13. Trubiano JA, Thursky KA, Stewardson AJ, Urbancic K, Worth LJ, Jackson C, et al. Impact of an Integrated Antibiotic Allergy Testing Program on Antimicrobial Stewardship: A Multicenter Evaluation. Clinical infectious diseases : an official publication of the Infectious Diseases Society of America. 2017;65(1):166-74.

14. Chua KYL, Vogrin S, Bury S, Douglas A, Holmes NE, Tan N, et al. The Penicillin Allergy Delabeling Program: A Multicenter Whole-of-Hospital Health Services Intervention and Comparative Effectiveness Study. Clinical infectious diseases : an official publication of the Infectious Diseases Society of America. 2020.

15. Devchand M, Urbancic KF, Khumra S, Douglas AP, Smibert O, Cohen E, et al. Pathways to improved antibiotic allergy and antimicrobial stewardship practice: The validation of a beta-lactam antibiotic allergy assessment tool. The journal of allergy and clinical immunology In practice. 2019;7(3):1063-5 e5.

16. Trubiano J, Vogrin S, Holmes NE, Chua K, Douglas A, Bourke J, et al. Development and Validation of a Penicillin Allergy Clinical Decision Rule. JAMA internal medicine. 2020.

17. Curran GM, Bauer M, Mittman B, Pyne JM, Stetler C. Effectiveness-implementation hybrid designs: combining elements of clinical effectiveness and implementation research to enhance public health impact. Med Care. 2012;50(3):217-26.

18. Presseau J, McCleary N, Lorencatto F, Patey AM, Grimshaw JM, Francis JJ. Action, actor, context, target, time (AACTT): a framework for specifying behaviour. Implement Sci. 2019;14(1):102.

19. James R, Upjohn L, Cotta M, Luu S, Marshall C, Buising K, et al. Measuring antimicrobial prescribing quality in Australian hospitals: development and evaluation of a national antimicrobial prescribing survey tool. J Antimicrob Chemother. 2015;70(6):1912-8.

20. Groups. AE. Therapeutic guidelines: antibiotic. Version 15 ed. Melbourne: Therapeutic Guidelines Limited; 2014.

21. Devchand M, Urbancic K, Khumra S, Walker S, Douglas A, Smibert O, et al. Pathways to improved antibiotic allergy practice - the validation of a beta-lactam antibiotic allergy assessment tool to aid accurate phenotyping and management. Clinical and Translational Allergy Conference: 8th Drug Hypersensitivity Meeting, DHM. 2018;8(Supplement 3).

22. Rischin KJ, Mostaghim M, Rao A, Smith B, O'Brien TA, Trubiano JA, et al. ESCAPE-Allergy: Evaluating screening for children and adolescents with penicillin allergy. Journal of paediatrics and child health. 2022;58(1):83-9.

23. Trubiano JA, Vogrin S, Chua KYL, Bourke J, Yun J, Douglas A, et al. Development and Validation of a Penicillin Allergy Clinical Decision Rule. JAMA Intern Med. 2020;180(5):745-52.

24. Bourke J, Pavlos R, James I, Phillips E. Improving the Effectiveness of Penicillin Allergy De-labeling. The journal of allergy and clinical immunology In practice. 2015;3(3):365-34 e1.

25. Blumenthal KG, Shenoy ES, Varughese CA, Hurwitz S, Hooper DC, Banerji A. Impact of a clinical guideline for prescribing antibiotics to inpatients reporting penicillin or cephalosporin allergy. Annals of allergy, asthma & immunology : official publication of the American College of Allergy, Asthma, & Immunology. 2015;115(4):294-300 e2.

26. Trubiano JA, Smibert O, Douglas A, Devchand M, Lambros B, Holmes NE, et al. The Safety and Efficacy of an Oral Penicillin Challenge Program in Cancer Patients: A Multicenter Pilot Study. Open forum infectious diseases. 2018;5(12):ofy306.

27. Sekhon M, Cartwright M, Francis JJ. Development of a theory-informed questionnaire to assess the acceptability of healthcare interventions. BMC Health Serv Res. 2022;22(1):279.

#

| 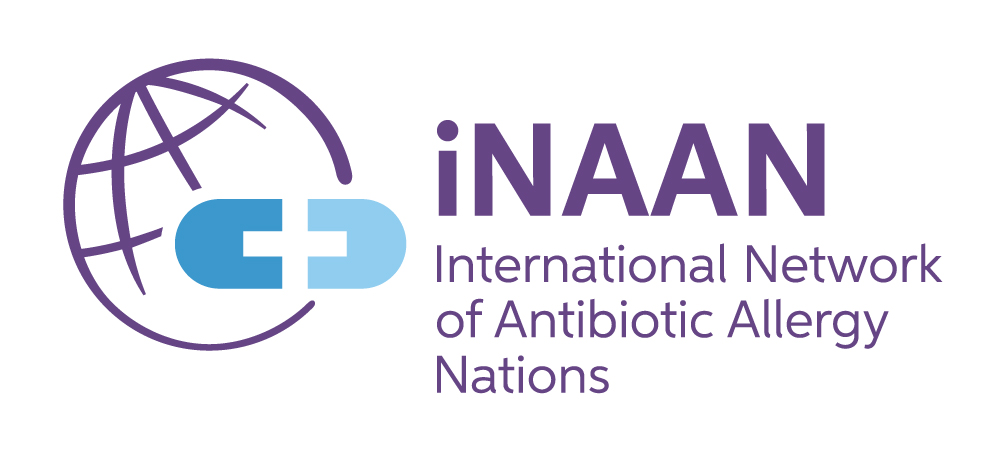 |
| --- |
| International Network of Antibiotic Allergy Nations (iNAAN) |
| Appendix 1: Antibiotic Allergy Assessment Tool |
| Version: 1.0  Date: 27 May 2022 |

# **Appendix 1** – Antibiotic Allergy Assessment Tool

The Antibiotic Allergy Assessment Tool(21) is a validated tool for the assessment of beta-lactam antibiotic allergies, including by non-allergists. The tool may be adapted for local use at participating sites, including paediatric sites(22).

**
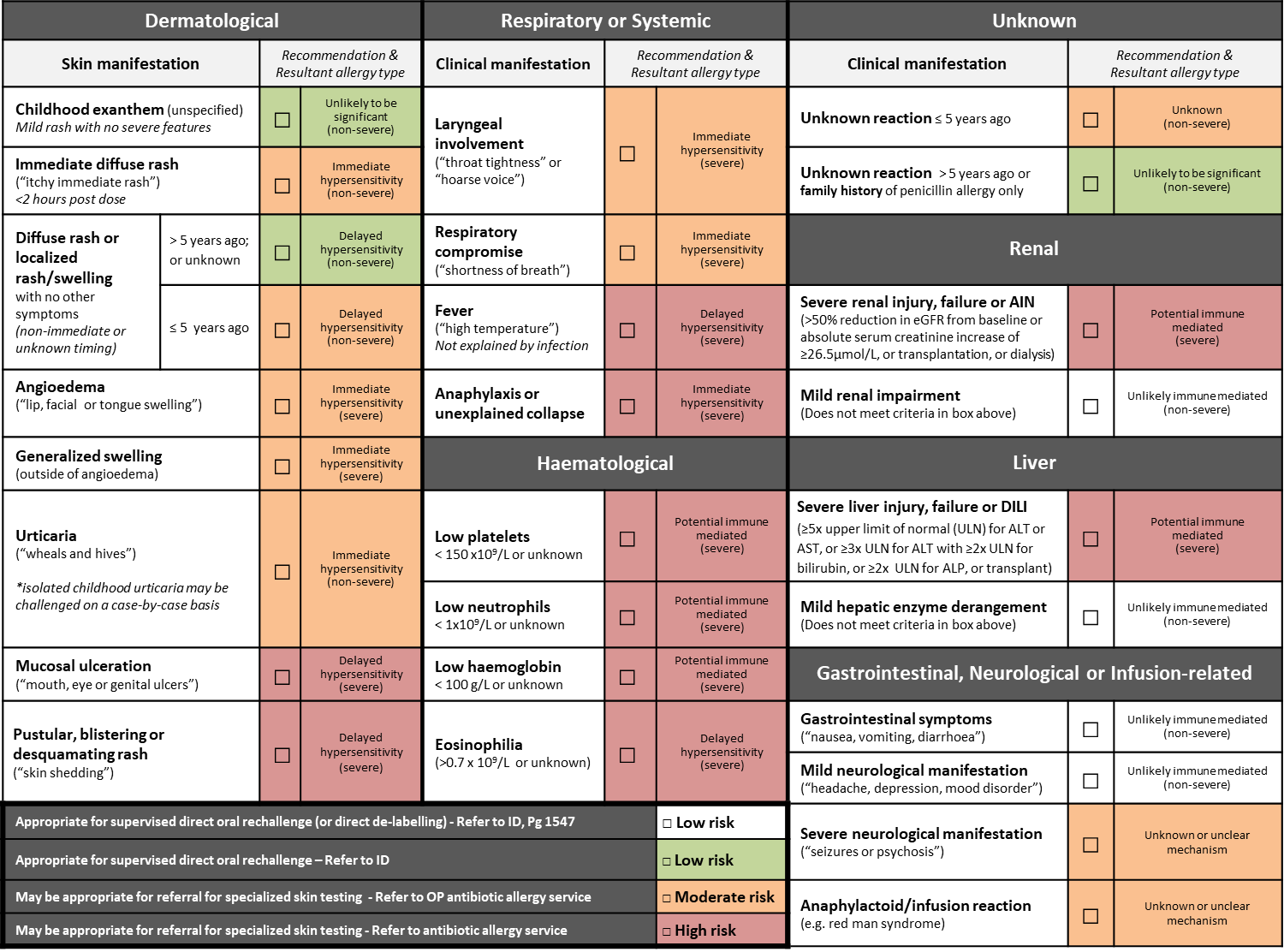
**

| 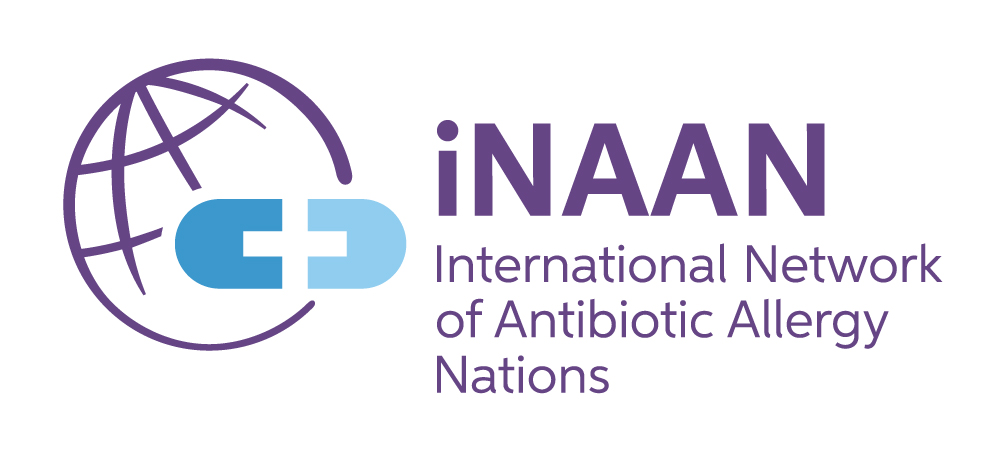 |
| --- |
| International Network of Antibiotic Allergy Nations (iNAAN) |
| Appendix 2: PEN-FAST |
| Version: 1.0  Date: 27 May 2022 |

## **Appendix 2.** The PEN-FAST Penicillin allergy clinical decision rule

PEN-FAST (*Pen*icillin allergy, *f*ive or fewer years ago, *a*naphylaxis/angioedema or *S*CAR and *t*reatment required for episode) is a simple point-of-care risk assessment tool for patient-reported penicillin allergies(23).


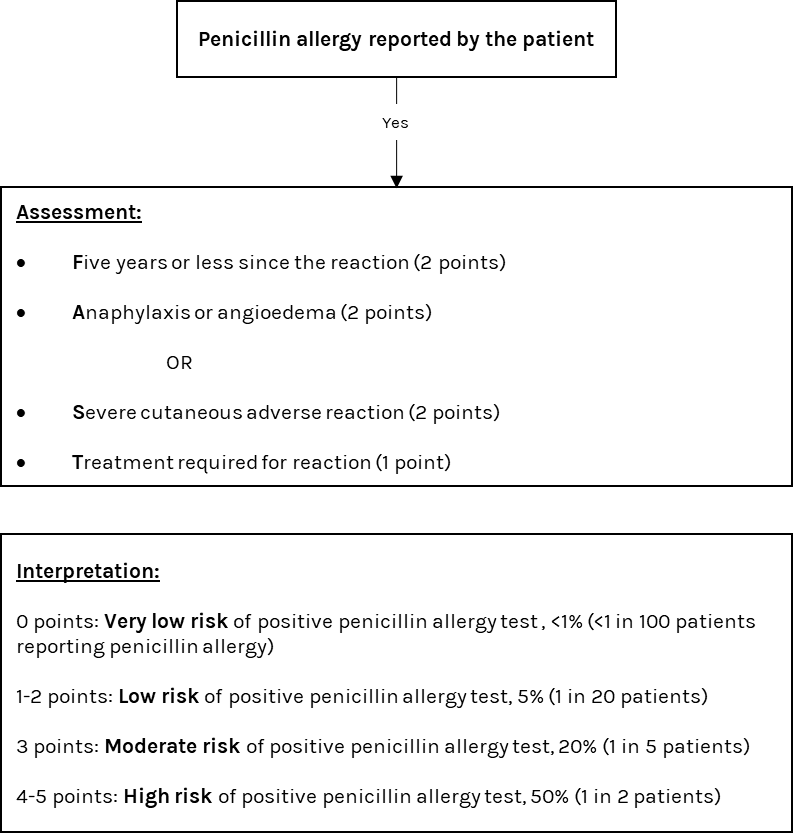


| 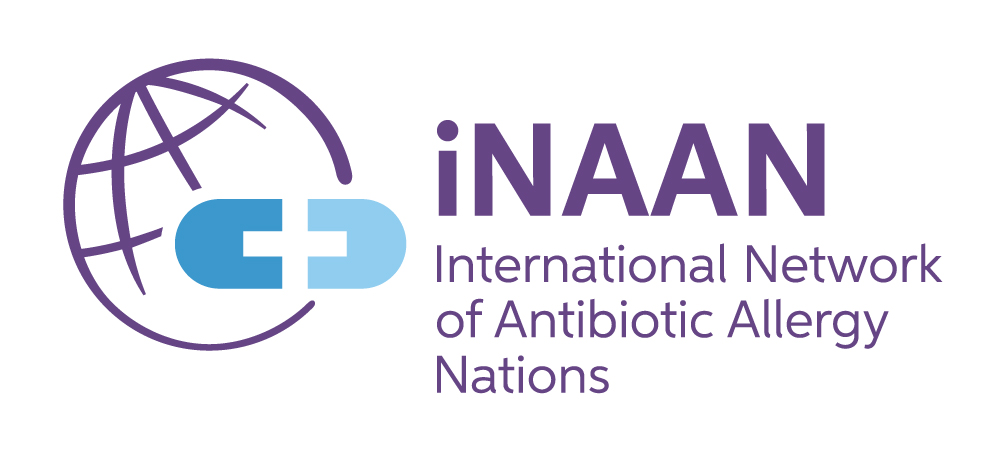 |
| --- |
| International Network of Antibiotic Allergy Nations (iNAAN) |
| Appendix 3: Oral Challenge Clinical Guideline |
| Version: 2.0  Date: 28 March 2023 |

**
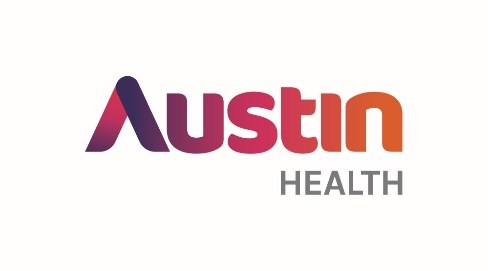
INFECTIOUS DISEASES DEPARTMENT**

GUIDELINE

| **ANTIBIOTIC ALLERGY ORAL CHALLENGE** |
| --- |

| **Staff this document applies to:** |
| --- |

- Infectious Diseases Department
- Austin Health nurses, doctors, and pharmacy staff

| **Related Austin Health policies, procedures or guidelines:** |
| --- |

[Austin Health Drug & Antibiotic Allergy Services (DAAS) Protocol](https://austinhealth.sharepoint.com/:w:/r/sites/OPPIC/_layouts/15/Doc.aspx?sourcedoc=%7BF15CD41E-EEA6-4F53-B439-D9E895704102%7D&file=austin_health_drug_antibiotic_allergy_services_daas_protocol.docx&action=default&mobileredirect=true&DefaultItemOpen=1)

[Antimicrobial Stewardship Antibiotic Allergy Ward Round](https://austinhealth.sharepoint.com/:w:/r/sites/OPPIC/_layouts/15/Doc.aspx?sourcedoc=%7B06593506-F83A-41F6-8160-314EA7223DD7%7D&file=antimicrobial_stewardship_antibiotic_allergy_ward_round_290520.docx&action=default&mobileredirect=true&DefaultItemOpen=1)

[Trimethoprim/Sulfamethoxazole Adverse Drug Reaction Protocol](https://austinhealth.sharepoint.com/:w:/r/sites/OPPIC/_layouts/15/Doc.aspx?sourcedoc=%7BD7BCBEB9-60DE-4F21-96E0-D80D7DB2C7E6%7D&file=tmp_smx_adr_protocol_2020_update.docx&action=default&mobileredirect=true&DefaultItemOpen=1)

[Antimicrobial Desensitisation Protocol](https://austinhealth.sharepoint.com/:w:/r/sites/OPPIC/_layouts/15/Doc.aspx?sourcedoc=%7B53796E1F-1A16-4D42-ADD8-6753887696E9%7D&file=antimicrobial_desensitisation_protocol_052020.docx&action=default&mobileredirect=true&DefaultItemOpen=1)

[Anaphylaxis – Initial Management](https://austinhealth.sharepoint.com/:w:/r/sites/OPPIC/_layouts/15/Doc.aspx?sourcedoc=%7B0C2BAA6F-73A7-4A35-9A12-BB50C1965680%7D&file=Anaphylaxis%20-%20initial%20management.docx&action=default&mobileredirect=true&DefaultItemOpen=1)

[Perioperative drug allergy guideline](https://austinhealth.sharepoint.com/sites/OPPIC/Guideline/Forms/AllItems.aspx?id=%2Fsites%2FOPPIC%2FGuideline%2Fperi%5Foperative%5Fdrug%5Fallergy%5Ftesting%2Epdf&parent=%2Fsites%2FOPPIC%2FGuideline)

| **Purpose**: |
| --- |

To provide guidance on antibiotic allergy assessment and management of an oral antibiotic challenge in patients who have an antibiotic allergy.

| **Background**: |
| --- |

Adverse drug reactions to antibiotics can be divided into two broad categories:

- **Type A reactions**: Non-immune mediated – for example, cytopenia, increased serum creatinine and gastrointestinal intolerance.
- **Type B reactions**: Immune-mediated – for example, rash, anaphylaxis, urticaria, angioedema, acute interstitial nephritis and severe cutaneous adverse reaction (SCAR). SCAR refers to a distinct group of diagnoses including Stevens-Johnson syndrome (SJS), Toxic epidermal necrolysis (TEN), Drug reaction with eosinophilia and systemic symptoms (DRESS) and Acute generalised erythematous pustulosis (AGEP).

The most common antibiotic allergies are to penicillins and sulfonamides.

**Penicillin Allergy**

A patient-reported “penicillin” allergy is documented in 9-15% of hospitalised patients^1^. These penicillin allergies are associated with inappropriate prescribing and inferior patient outcomes ^(1, 3)^. Protocolised oral antibiotic challenge has been successful in patients with remote and mild allergy histories^(24, 25)^. Austin Health experience with oral penicillin rechallenge has also been published^(26)^. Penicillin oral challenge should be considered in patients that currently need or may need beta-lactam based therapy in the future.

Note:

- Penicillin allergy pre-1960 is likely due to phenoxymethylpenicillin or benzylpenicillin. Penicillin allergy post-1960 may be related to flucloxacillin/dicloxacillin or amoxicillin (post-1972).

**Trimethoprim-sulfamethoxazole Allergy**

Sulfonamide antibiotic allergy is the second most reported class of antibiotic allergy^6^.

Patients with HIV are at a higher risk for developing allergic reactions to sulfonamide antimicrobials.

Due to molecular structure differences, there is a very low risk of cross-allergenicity between sulfonamide antibiotics and sulfonamide non-antibiotic agents (e.g.; furosemide, sulfonylureas).

Protocolised direct oral challenge in patients with a low to moderate risk trimethoprim-sulfamethoxazole allergy has been demonstrated to be a safe and effective alternative to trimethoprim-sulfamethoxazole desensitisation protocols^6,7^. Trimethoprim-sulfamethoxazole oral challenge should be considered in patients that currently need or may need sulfonamide antibiotic -based therapy in the future. Delabeling of low to moderate risk sulfonamide antibiotic allergy labels enables optimal antibiotic prescribing, particularly in immunocompromised patients who may require trimethoprim-sulfamethoxazole treatment or prophylaxis therapy.

| **Definitions and Abbreviations:** |
| --- |

- **Penicillin –** Phenoxymethylpenicillin, benzylpenicillin, benzathine penicillin, penicillin “unspecified”
- **Aminopenicillin** – Amoxicillin, ampicillin, amoxicillin-clavulanate
- **TMP**-**SMX** – Trimethoprim-sulfamethoxazole
- **ADR** – Adverse drug reaction
- **MPE** – Maculopapular exanthema (rash without angioedema, urticaria, blistering, desquamation, internal organ involvement and/or mucosal involvement).
- **SCAR** – Severe cutaneous adverse reaction (e.g., Stevens-Johnson syndrome, toxic epidermal necrolysis, drug reaction with eosinophilia and systemic symptoms, acute generalised exanthematous pustulosis)
- **Type A** – Non-immune mediated pharmacologically predictable side effect (e.g., headache, gastrointestinal upset)
- **Type B** – Immune mediated ADR (e.g., anaphylaxis, urticaria, angioedema, rash, SCAR, acute interstitial nephritis, drug induced liver injury)

| **Oral Antibiotic Challenge - Inclusion & Exclusion criteria (Inpatient/ Outpatient):** |
| --- |

**Inclusion criteria:**

1. Age > 16 years
2. Active Infectious Diseases inpatient/consult patient or patient identified by antimicrobial stewardship or Drug and Antibiotic Allergy Service (DAAS)
3. **For an INPATIENT oral challenge,** history of penicillins, aminopenicillin, TMP-SMX, or unspecified “sulfa” allergy that is **low risk.** (See [Figure 1](#_Flow_chart_for))

- **Low risk** allergy criteria (either/or) - Use [Appendix 1](#_Appendix_1:_Clinical) and [Appendix 2](#_Appendix_1:_Austin) to assess antibiotic allergy risk:
  1. Unknown reaction > 5 years previous or date that can’t be recalled
  2. Type A ADR reaction where direct delabeling is not accepted by the patient
  3. History of benign childhood rash, non-urticarial rash, MPE or rash unspecified > 5-10 years previous*
  4. Local IM penicillin injection site reaction (only)

******Patients reporting a benign childhood exanthem that is described as isolated urticaria may be challenged on a case-by-case basis*

- The PEN-FAST Penicillin Allergy Clinical Decision Rule (Appendix 3) may be used in lieu of the Antibiotic Allergy Assessment Tool (Appendix 2) to guide a risk assessment and consideration of oral challenge in patient-reported penicillin allergies.

**Exclusion criteria:**

1. Haemodynamically unstable patients
2. Pregnancy
3. Allergy history unavailable due to patient cognitive impairment and no collateral history
4. History of antibiotic-associated anaphylaxis
5. History of antibiotic-associated Severe Cutaneous Adverse Reactions (SCAR)
6. History of acute kidney injury or severe liver impairment associated with antibiotic therapy
7. If ICU admission during current episode (relative contraindication) – use clinical discretion
8. Currently prescribed: prednisolone > 25 mg daily (or equivalent), systemic vasoconstrictors including terlipressin or H1-antagonist antihistamines

If the patient does not meet inclusion/exclusion criteria, they can be referred to the Outpatient Antibiotic Allergy Clinic if likely to require future antibiotic therapy.

| **Figure 1: Flow chart for determining suitability of patients for INPATIENT antibiotic oral challenge** |
| --- |


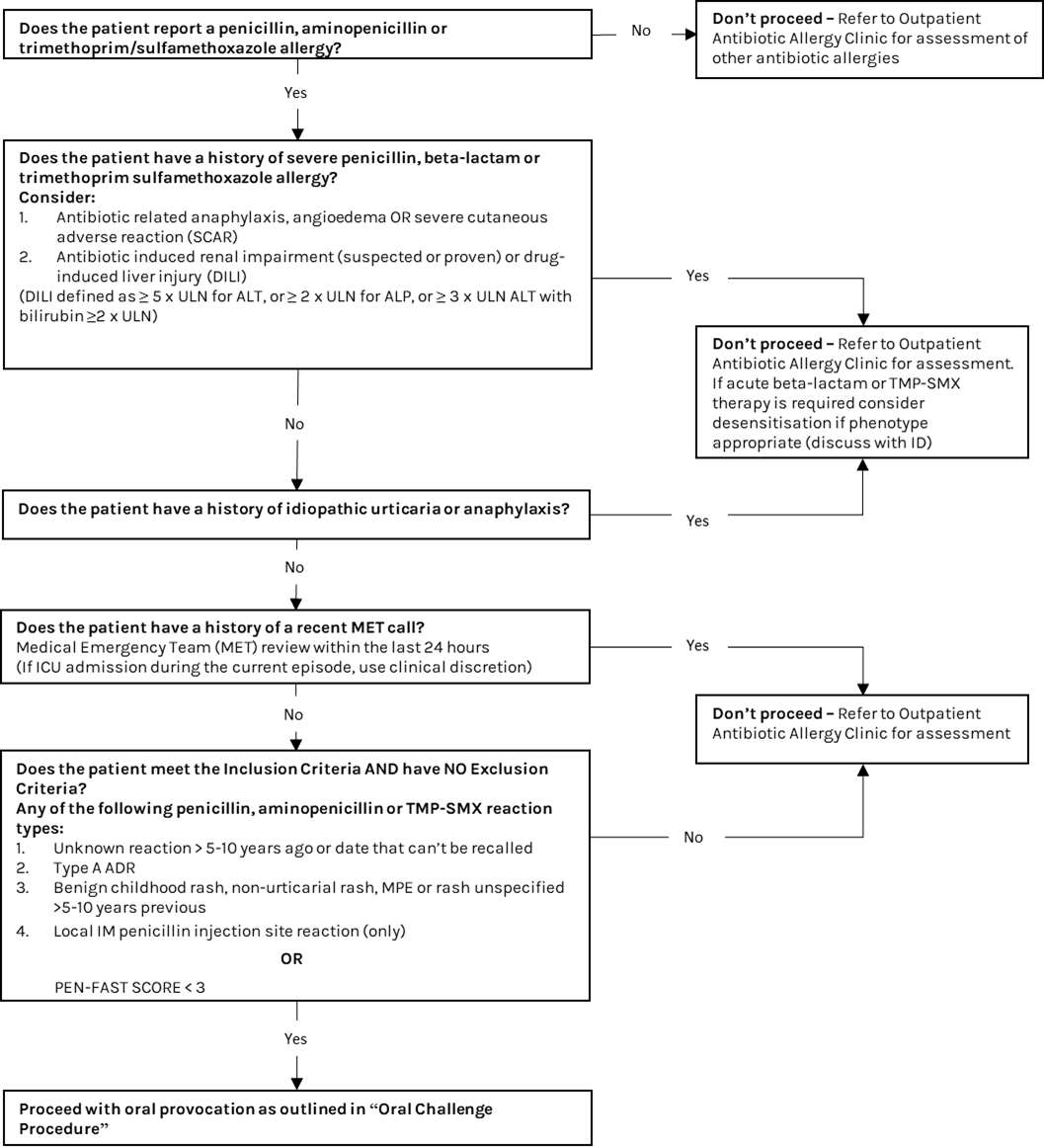


| **Oral challenge procedure** |
| --- |

**Inpatient antibiotic oral challenge**

**Inpatient oral challenge to be performed only after consultation with and consent by the Infectious Diseases Department,** with infectious diseases medical or allergy nursing staff on site and available to be in attendance in the setting of an acute adverse event**.**

1. Ensure patient meets the criteria for antibiotic oral challenge ([See Figure 1](#_Figure_1:_Flow))

If the patient does not meet the criteria for inpatient antibiotic oral challenge, see [Appendix](#_Appendix_4:_Recommendations_1) 4 for antibiotic recommendations for patients who report a penicillin allergy.

For recommendations on alternative agents in patients with trimethoprim-sulfamethoxazole allergy, see the [Trimethoprim/Sulfamethoxazole Adverse Drug Reaction Protocol or consult ID.](https://austinhealth.sharepoint.com/:w:/r/sites/OPPIC/_layouts/15/Doc.aspx?sourcedoc=%7BD7BCBEB9-60DE-4F21-96E0-D80D7DB2C7E6%7D&file=tmp_smx_adr_protocol_2020_update.docx&action=default&mobileredirect=true&DefaultItemOpen=1)

1. Obtain verbal consent from patient’s treating Unit to perform inpatient antibiotic oral challenge.
2. Patient to be consented by Infectious Diseases / Immunology Consultant, Fellow or Registrar, or delegate as per Infectious Diseases advice.
3. Coordinate timing of oral challenge with Infectious Diseases Department: 9am-3pm Monday to Friday
4. Drug order to be charted by Infectious Diseases / Immunology Consultant, Fellow or Registrar., using Cerner “ID Oral” Antibiotic Challenge Careset.

***Oral Penicillin Challenge***

Single dose: phenoxymethylpenicillin 250 mg or amoxicillin 250 mg or flucloxacillin 250 mg

- If reported allergy is phenoxymethylpenicillin or benzylpenicillin – give phenoxymethylpenicillin
- If reported allergy is amoxicillin or ampicillin – give amoxicillin
- If reported allergy is flucloxacillin – give flucloxacillin
- If reported allergy is “unknown penicillin” – give amoxicillin. Phenoxymethylpenicillin may be considered if patient reports “unknown penicillin” prior to 1970.
- If reported allergy is a Type A ADR (with clear history) and acute beta-lactam therapy required, administration of full treatment dose can proceed without test dose

***Oral Trimethoprim-Sulfamethoxazole Challenge***

Single dose: ½ x Trimethoprim-sulfamethoxazole 160/800 mg tablet. (This is half a “double strength” tablet).

1. Medical or specialised allergy nursing staff to be available during 1.5 hours of oral challenge observation period and available thereafter to attend patient immediately if required.
2. Resuscitation equipment must be available on the ward, however PRN prescription NOT required
3. NURSING requirements:
   1. Immediately prior to oral challenge, perform baseline patient observations (HR, BP, Sats, RR)
   2. Administer **orally** either a single dose of phenoxymethylpenicillin 250 mg or amoxicillin 250 mg or flucloxacillin 250 mg or ½ x trimethoprim-sulfamethoxazole 160/800 mg tablet as charted in Cerner (unscheduled order) by Infectious Diseases Department **(only)**
   3. Perform 30 minutely observations for 1.5 hours post oral challenge
4. If there is a history of delayed MPE and no current antibiotic requirements, consider additional testing with a 3-day oral challenge (phenoxymethylpenicillin 500 mg BD or amoxicillin 500 mg BD or flucloxacillin 500 mg BD or trimethoprim-sulfamethoxazole 160/800 mg DAILY) to exclude delayed hypersensitivity.
5. If no evidence of reaction, Infectious Diseases Department to remove penicillin, aminopenicillin or trimethoprim-sulfamethoxazole allergy from electronic medical record (Cerner) immediately post challenge. A letter will be sent to the patient, their other treating clinicians and general practitioner to notify them of the allergy removal.
6. The patient is to be provided with Drug and Antibiotic Allergy Service (DAAS) dedicated 24-hour phone contact details to report any symptoms of a delayed reaction after the initial monitoring period.

**Outpatient antibiotic oral challenge (at Antibiotic Allergy Clinic)**

(Internal referrals can be made by [paper referral](https://austinhealth.sharepoint.com/:b:/s/Clinical/EasfX64M71pEvwpGxaAmpM4BWmuBtx6CX4GZeForLaDB4g?e=UBCQ1l) - email: [antibiotic.allergy@austin.org.au](mailto:antibiotic.allergy@austin.org.au) )

As per Inpatient antibiotic oral challenge procedure above, with the following modifications:

***Oral Penicillin Challenge***

Oral challenge may be preceded by skin testing.

Patients with a history of moderate to severe immediate allergy symptoms or PEN-FAST score >2 ^8^ (see [Appendix](#_Appendix_3:_Trimethoprim-sulfametho) 3) receive a two-dose oral challenge:

- 50 mg phenoxymethylpenicillin /amoxicillin/flucloxacillin, then if no reaction after 30 minutes
- 200 mg phenoxymethylpenicillin /amoxicillin/flucloxacillin.

Patients with a history of moderate to severe delayed allergy symptoms receive a single dose oral challenge:

- 250 mg phenoxymethylpenicillin /amoxicillin/flucloxacillin, with nursing observations for 1.5 hours, then;
- 3-day course oral challenge phenoxymethylpenicillin /amoxicillin/flucloxacillin, 500 mg BD.
- If a patient has received skin testing, routine follow-up photographs of the skin testing site are taken by the patient at 24 hours and 48 hours. Verbal and written instructions are provided to the patient to

send skin testing site photographs to the DAAS phone which are then reviewed by DAAS Medical staff.

- Patients may also send photographs of a delayed rash or other reaction (regardless of prior skin testing) to the DAAS phone for Medical review.

***Oral Trimethoprim-Sulfamethoxazole Challenge***

Patients with a moderate risk history, i.e.: recent ( <5 years) immediate symptoms OR remote ( >5 years) anaphylaxis, receive a two-dose oral challenge:

- 1 mL trimethoprim-sulfamethoxazole 40 mg-200 mg/5 mL oral suspension, then if no reaction after 30 minutes
- 9 mL trimethoprim-sulfamethoxazole 40 mg-200 mg/5 mL oral suspension

If not requiring acute TMP-SMX therapy, consider 3-day course of oral trimethoprim-sulfamethoxazole 160/800 mg, 1 tablet DAILY.

- If a patient has received skin testing, routine follow-up photographs of the skin testing site are taken by the patient at 24 hours and 48 hours. Verbal and written instructions are provided to the patient to send skin testing site photographs to the DAAS phone which are then reviewed by DAAS Medical staff.
- Patients may also send photographs of a delayed rash or other reaction (regardless of prior skin testing) to the DAAS phone for Medical review.

| **Author/Contributors:** |
| --- |

Updated - November 2022

A/Prof Jason Trubiano (Infectious Diseases Physician, Drug and Antibiotic Allergy Services)

Ms Elise Mitri (Antibiotic Allergy Pharmacist, Drug and Antibiotic Allergy Services)

Updated: February 2022

A/Prof Jason Trubiano (Infectious Diseases Physician, Drug and Antibiotic Allergy Services)

Dr Kyra Chua (Infectious Diseases Physician, Drug and Antibiotic Allergy Services)

Dr Jamie Waldron (Immunology Fellow, Austin Health)

Ms Elise Mitri (Antibiotic Allergy Pharmacist, Drug and Antibiotic Allergy Services)

Original Authors – 2019:

A/Prof Jason Trubiano (Infectious Diseases, Drug and Antibiotic Allergy Services)

Misha Devchand (Infectious Diseases Pharmacist)

Wendy Stevenson (Allergy Nurse Consultant)

| **Legislation/References/Supporting Documents:** |
| --- |

1. Trubiano JA, Chen C, Cheng AC, Grayson ML, Slavin MA, Thursky KA, et al. Antimicrobial allergy ‘labels’ drive inappropriate antimicrobial prescribing: lessons for stewardship. The Journal of Antimicrobial Chemotherapy. 2016; 71(6): 1715-22

2. Macy E, Contreras R. Healthcare use and serious infection prevalance associated with penicillin “allergy” in hospitalised patients: A cohort study. The Journal of Allergy and Clinical Immunology. 2015; 133(3): 790-6.

3. Bourke J, Pavlos R, James I, Phillips E. Improving the effectiveness of penicillin allergy de-labeling. The Journal of Allergy and Clinical Immunology in Practice. 2015; 3(3): 365-34 e1.

4. Blumenthal KG, Shenoy ES, Varughese CA, Hurwitz S, Hooper DC, Banerji A. Impact of a clinical guideline for prescribing antibiotics to inpatents reporting penicillin or cephalosporin allergy. Annals of allergy, asthma and immunology: official publication of the Americna College of Allergy, Asthma & Immunology. 2015; 11(4): 294-300 e2.

5. Trubiano J, Vogrin S, Copaescu A, Nasra M, Douglas A, Holmes N, et al. Direct oral penicillin challenge for penicillin allergy delabelling as a health services intervention: A multicentre cohort study. Allergy. 2021. <https://doi.org/10.1111/all.15169>

6. Krantz S, Stone, C, Abreo A, Phillips E. Oral challenge with trimethoprim-sulfamethoxazole in patients with “sulfa” antibiotic allergy. The Journal of Allergy and Clinical Immunology: In Practice. 2020; 8(2): 757-760

7. Rose M, Vogrin S, Chua K, Drewett G, Douglas A, Slavin M et al. The safety and efficacy of direct oral challenge in trimethoprim-sulfamethoxazole antibiotic allergy. The Journal of Allergy and Clinical Immunology: In Practice. 2021; 9(1): 3847-3849

8. Trubiano JA, Vogrin S, Chua KYL, Bourke J, Yun J, Douglas A, Stone CA, Yu R, Groenendijk L, Holmes NE, Phillips EJ. Development and Validation of a Penicillin Allergy Clinical Decision Rule. JAMA Intern Med. 2020 May 1;180(5):745-752. doi: 10.1001/jamainternmed.2020.0403.

9. Devchand M, Urbancic K, Khumra S, Douglas A, Smibert O, Cohen E et al. Pathways to improved antibiotic biotic allergy and antimicrobial stewardship practice – The validation of a beta-lactam antibiotic allergy assessment tool. The journal of allergy and clinical immunology in practice. 2018

10. Krantz M, Stone C, Abreo A, Phillips E. Reply to “the safety and efficacy of direct oral challenge in trimethoprim-sulfamethoxazole antibiotic allergy”. The Journal of Allergy and Clinical Immunology: In Practice. 2021; 9(10): 3849-3850

| **Authorised/endorsed by:** |
| --- |

A/Prof Jason Trubiano (Director of Drug and Antibiotic Allergy Services)

Drug and Therapeutics Committee (DTC), Austin Health, Nov 2022

| **Primary Person/Department Responsible for Document:** |
| --- |

A/Prof Jason Trubiano - Infectious Diseases/Drug and Antibiotic Allergy Services

| **Appendix 1: Clinical assessment of a patient’s antibiotic allergy history** |
| --- |


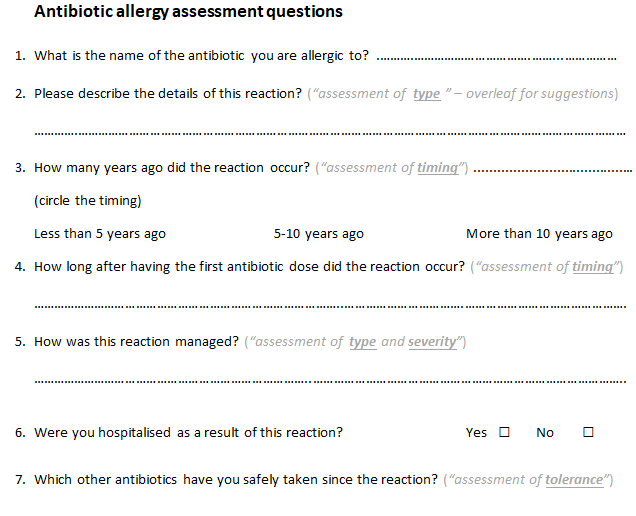


***Use the above answers to tick the correct allergy phenotype on the Antibiotic Allergy Assessment Tool*** [*(Appendix 2)*](#_Appendix_1:_Austin)


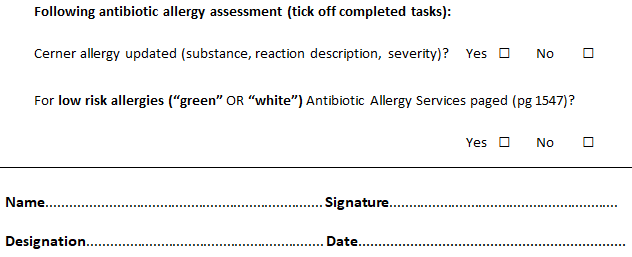


| **Appendix 2: Austin Health Antibiotic Allergy Assessment Tool** |
| --- |


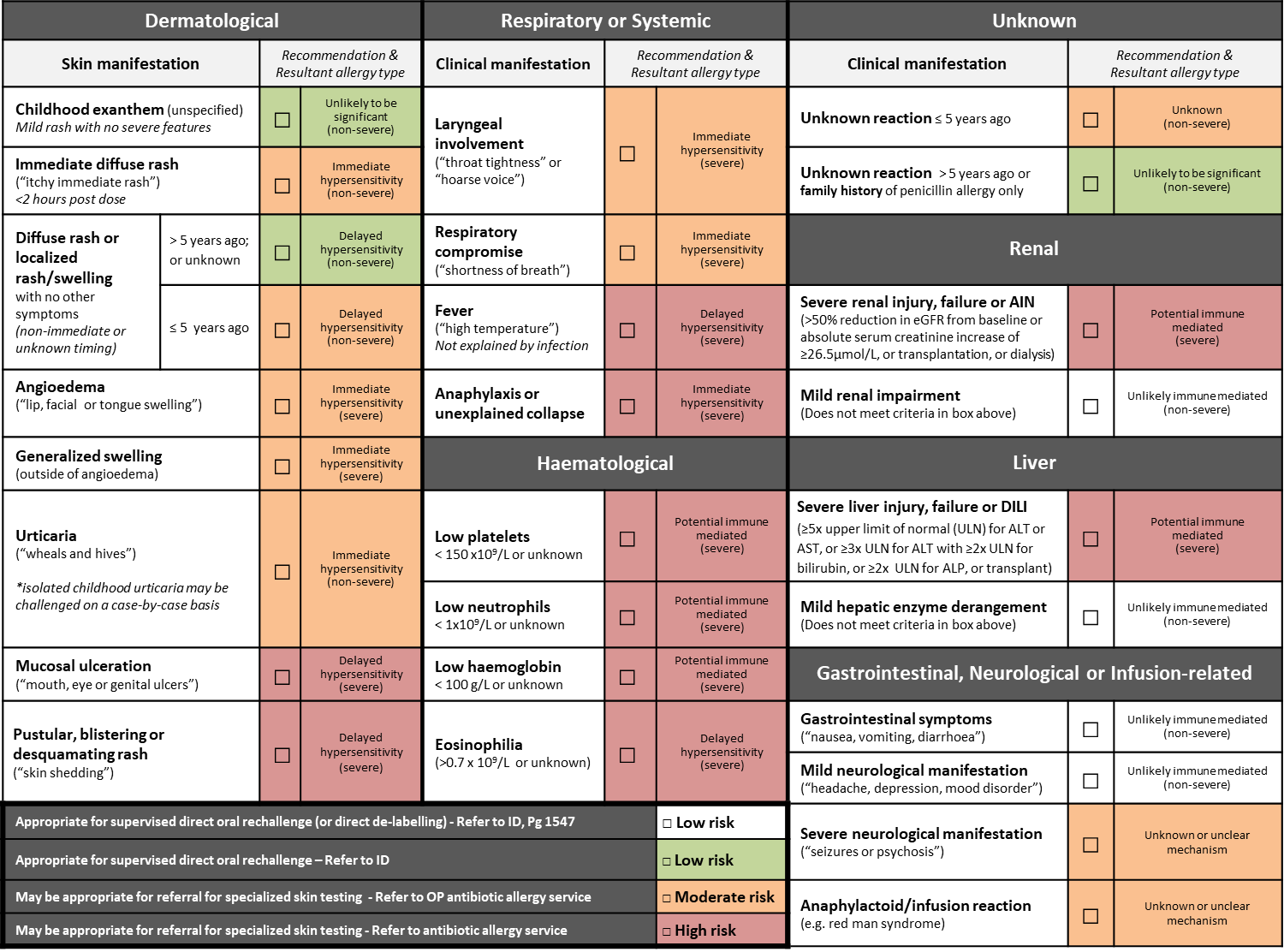


| **Appendix 3: PEN-FAST – Penicillin Allergy Clinical Decision Rule** |
| --- |

**Designed for point-of-care risk assessment of patient-reported penicillin allergies.**


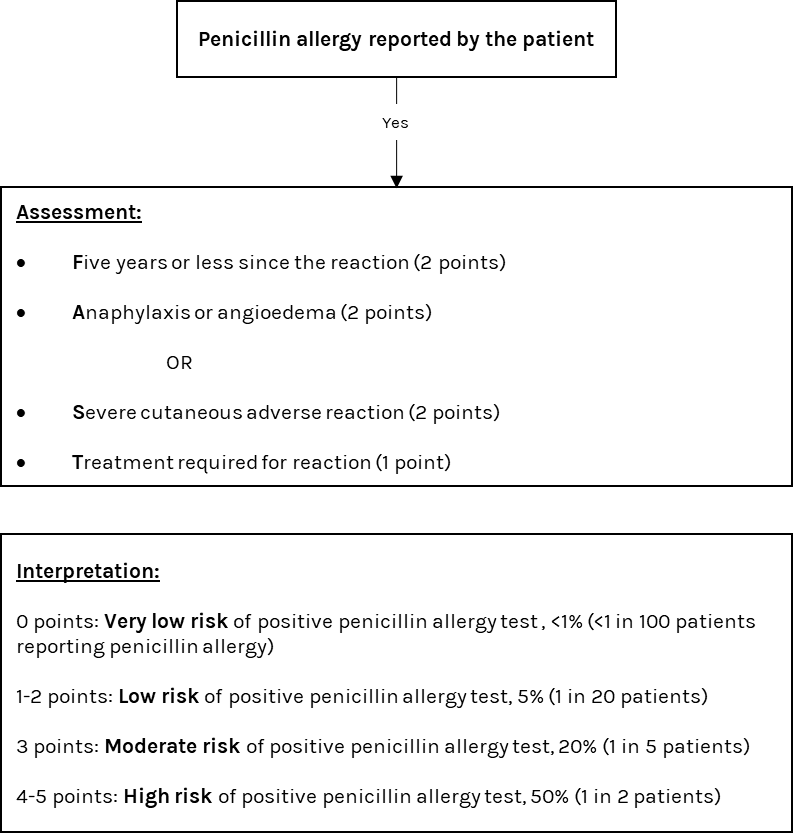


*Trubiano JA, Vogrin S, Chua KYL, Bourke J, Yun J, Douglas A, Stone CA, Yu R, Groenendijk L, Holmes NE, Phillips EJ. Development and Validation of a Penicillin Allergy Clinical Decision Rule. JAMA Intern Med. 2020 May 1;180(5):745-752. doi: 10.1001/jamainternmed.2020.0403.*

| **Appendix 4: Recommendations for antibiotic therapy in patients with a reported penicillin allergy** |
| --- |

***Table 1. Recommendations for antibiotic use in patients with a primary reported penicillin allergy.***

| **Allergy Phenotype** | **Recommendation** |
| --- | --- |
| **Immediate Penicillin Hypersensitivity (non-severe)**  *IgE-mediated*  E.g: urticaria | Avoid penicillins and 1^st^ generation cephalosporins (except cefazolin)  Consider ≥ 2^nd^ generation cephalosporin if history of remote immediate hypersensitivity or mild presentation (urticaria only)  Safe for carbapenem  Safe for monobactams (aztreonam) |
| **Immediate Penicillin Hypersensitivity (severe)**  *IgE-mediated*  E.g: anaphylaxis | Avoid penicillins, 1^st^ and 2^nd^ generation cephalosporins  Consider ≥ 3^rd^ generation cephalosporin  Consider carbapenem  Safe for monobactams (aztreonam) |
| **Delayed Penicillin Hypersensitivity (non-severe)**  *T-cell-mediated*  E.g: mild rash | Avoid penicillins  Avoid aminocephalosporins (cephalexin/cefaclor) only if primary allergy to aminopenicillins (amoxicillin/ampicillin)  Safe for cefazolin  Safe for ≥ 2^nd^ generation cephalosporins  Safe for carbapenems and monobactams |
| **Delayed Penicillin Hypersensitivity (severe)**  *T-cell-mediated*  E.g: SCAR | Avoid all beta-lactams (penicillins, cephalosporins)  Consider carbapenem  Safe for monobactams (aztreonam) |

| 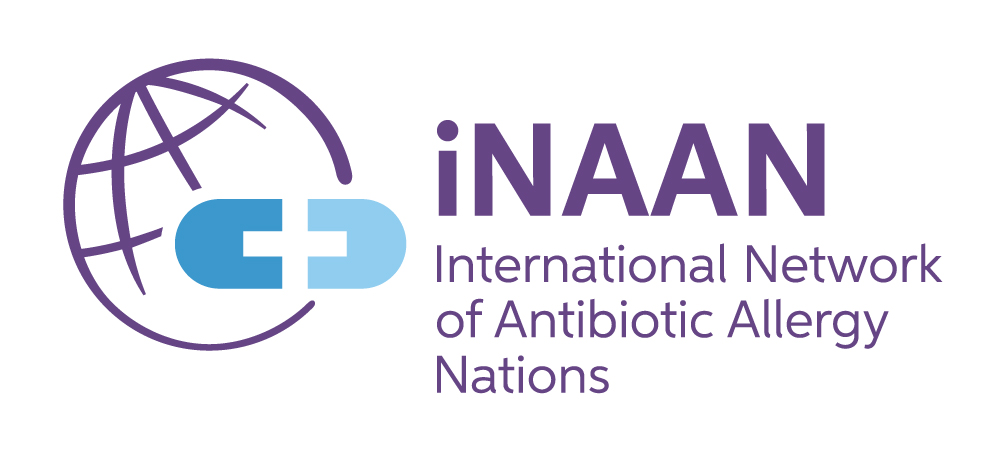 |
| --- |
| International Network of Antibiotic Allergy Nations (iNAAN) |
| Appendix 4: NAAN Smartphone App |
| Version: 1.0  Date: 27 May 2022 |

# National Antibiotic Allergy Network Smartphone App

| 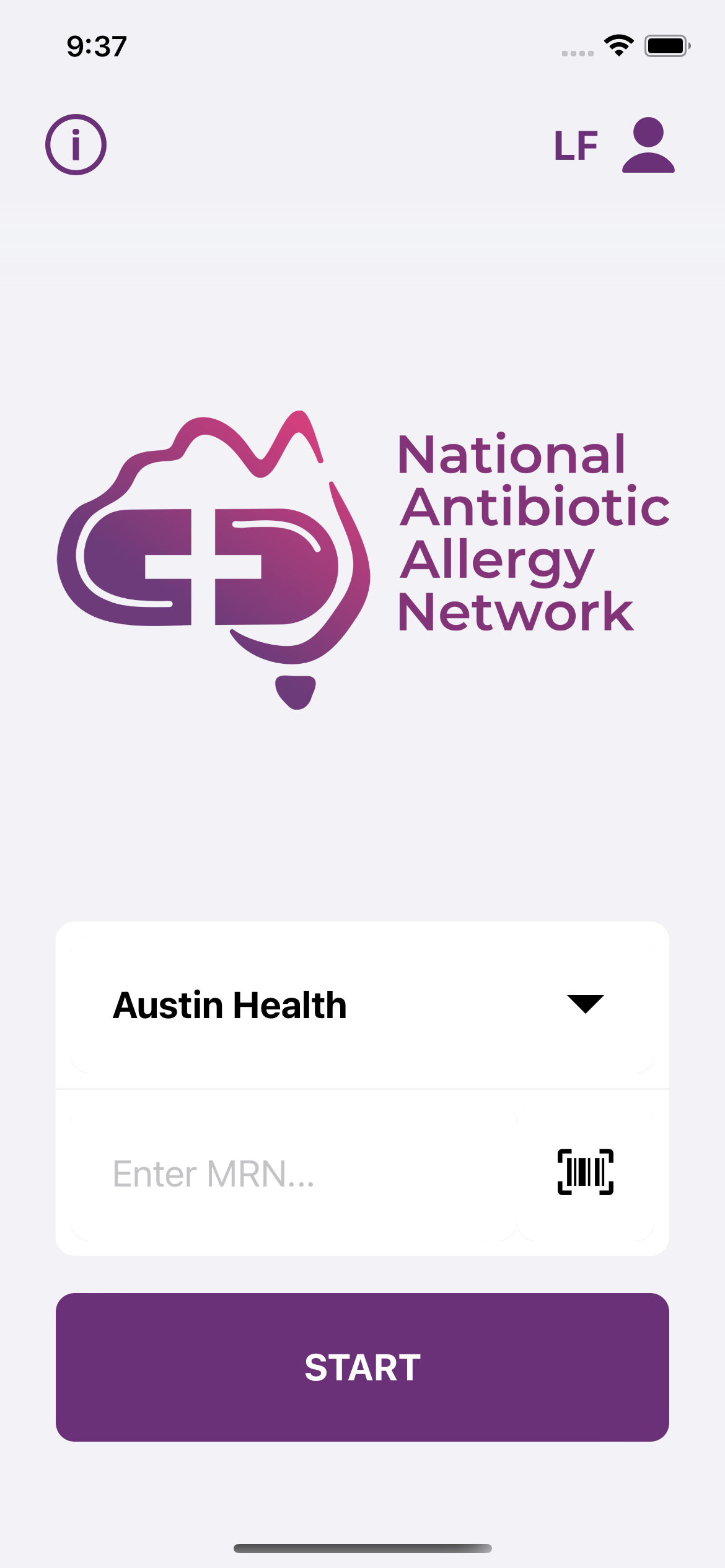 | 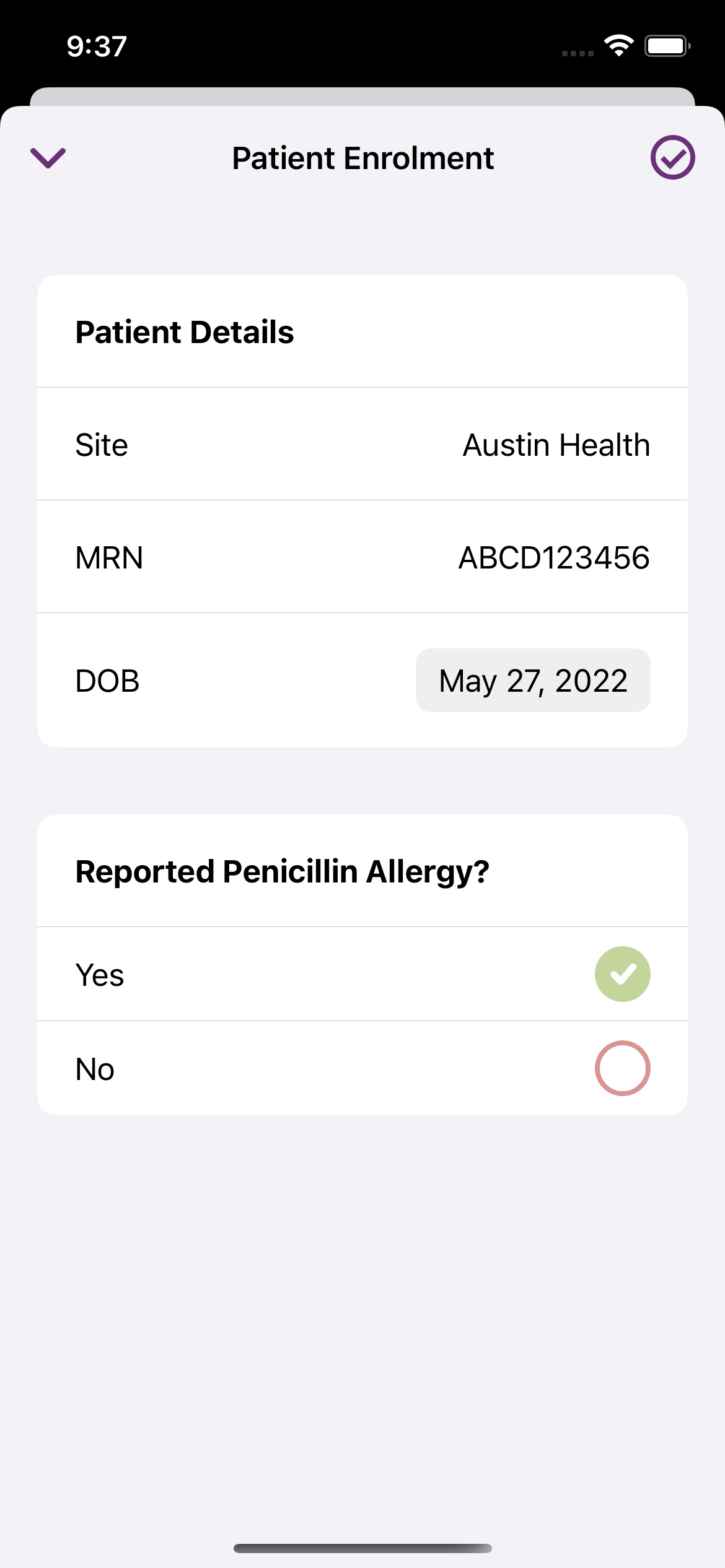 | 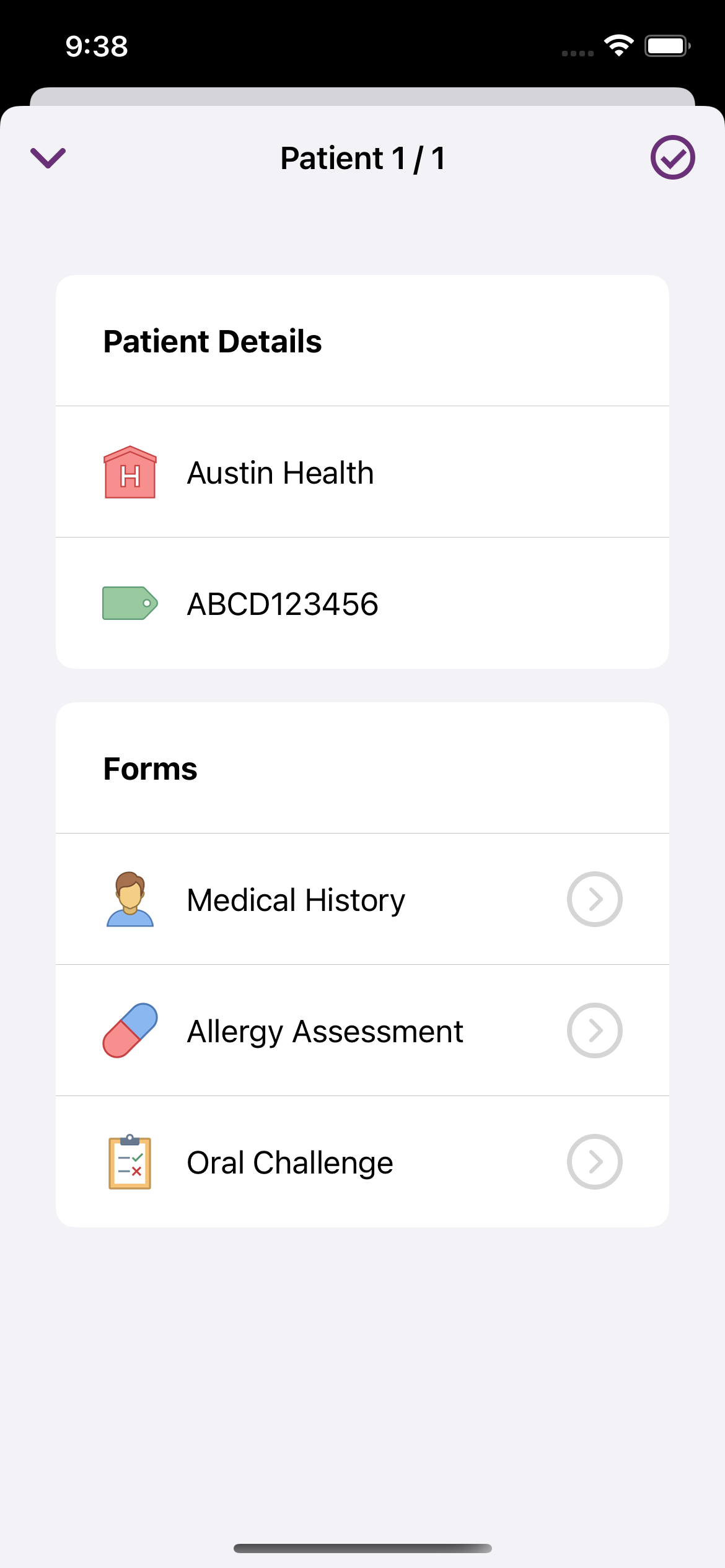 |
| --- | --- | --- |

**Introduction**

The National Antibiotic Allergy Network (NAAN) smartphone app is a digital tool designed for assessing and collecting data on antibiotic allergy reactions and direct oral penicillin challenges. It provides a user friendly and intuitive way of collecting data at the bedside and allows this data to be securely entered into the REDCap database, hosted by BioGrid.

**Use:**

The smartphone app is intended to be used by study investigators who are collecting data from patients at the time of antibiotic allergy assessment and when performing a direct oral penicillin challenge.

**Setup:**

The smartphone app is built for the Apple iOS platform and can be used on iPhone or iPad devices. Study investigators will be able to download the app to their personal device or can utilise a work device. Study investigators will have unique username and password details emailed to them to login to the smartphone app. Only authorised users will be able to use the app.

**Home Screen:**

After accepting the Legal Disclaimer and successfully logging in, investigators are asked to select their clinical site, and enter the patient’s hospital unique record number. This can be done manually, or by scanning a patient’s bradma barcode.

**Enrolment:**

If a patient is not yet enrolled in the study, the enrolment form is presented, where the following information is collected: clinical site, unique record number, date of birth, sex, ethnicity and ATSI status, and confirmation of a reported penicillin allergy.

| 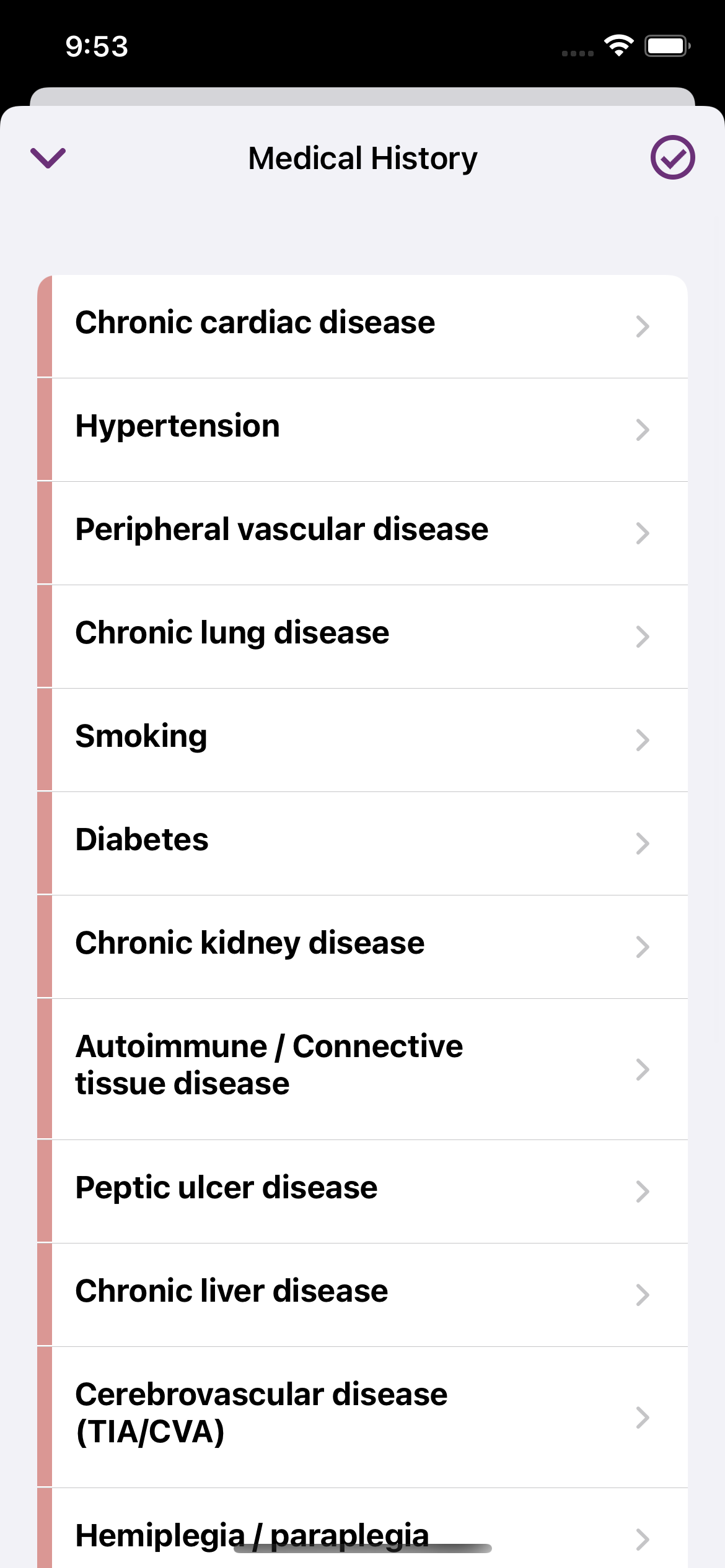 | 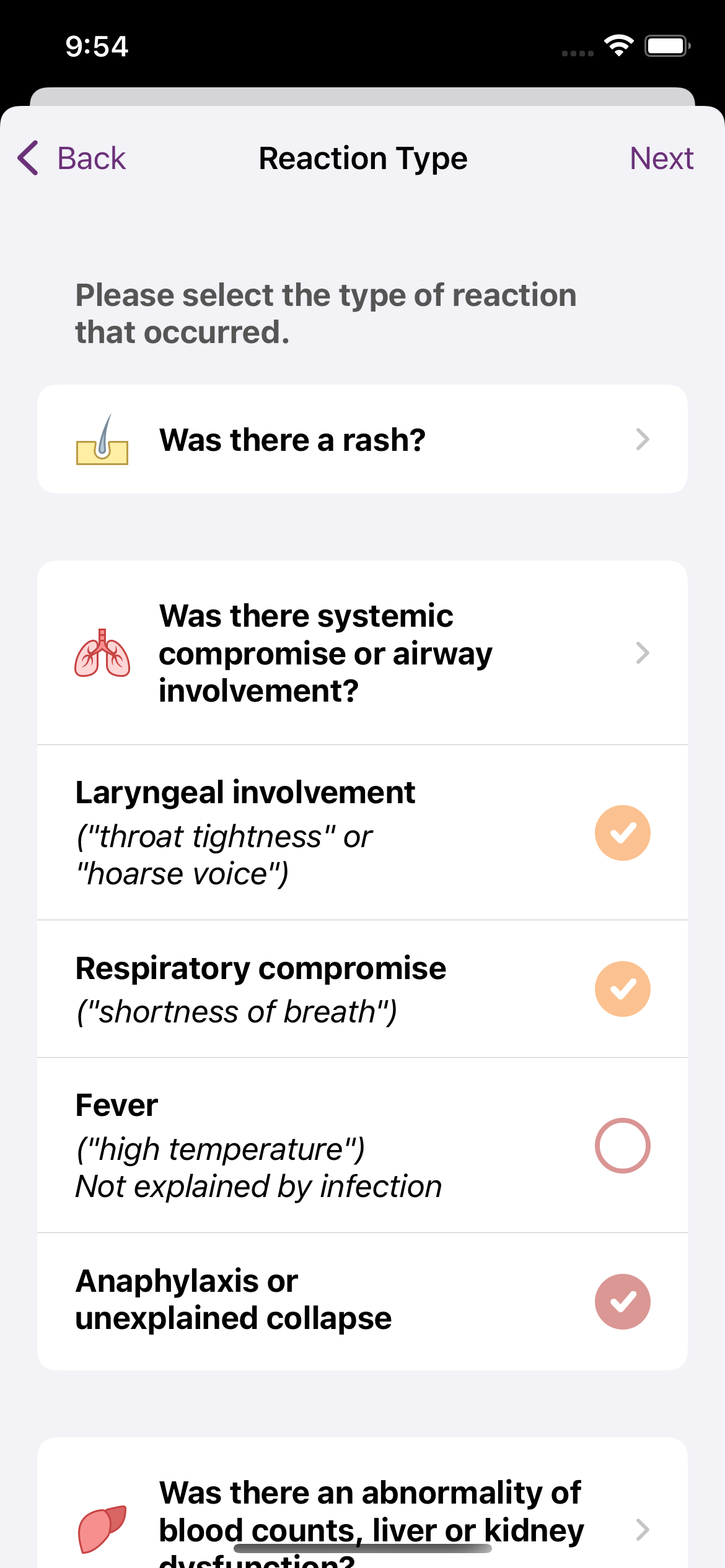 | 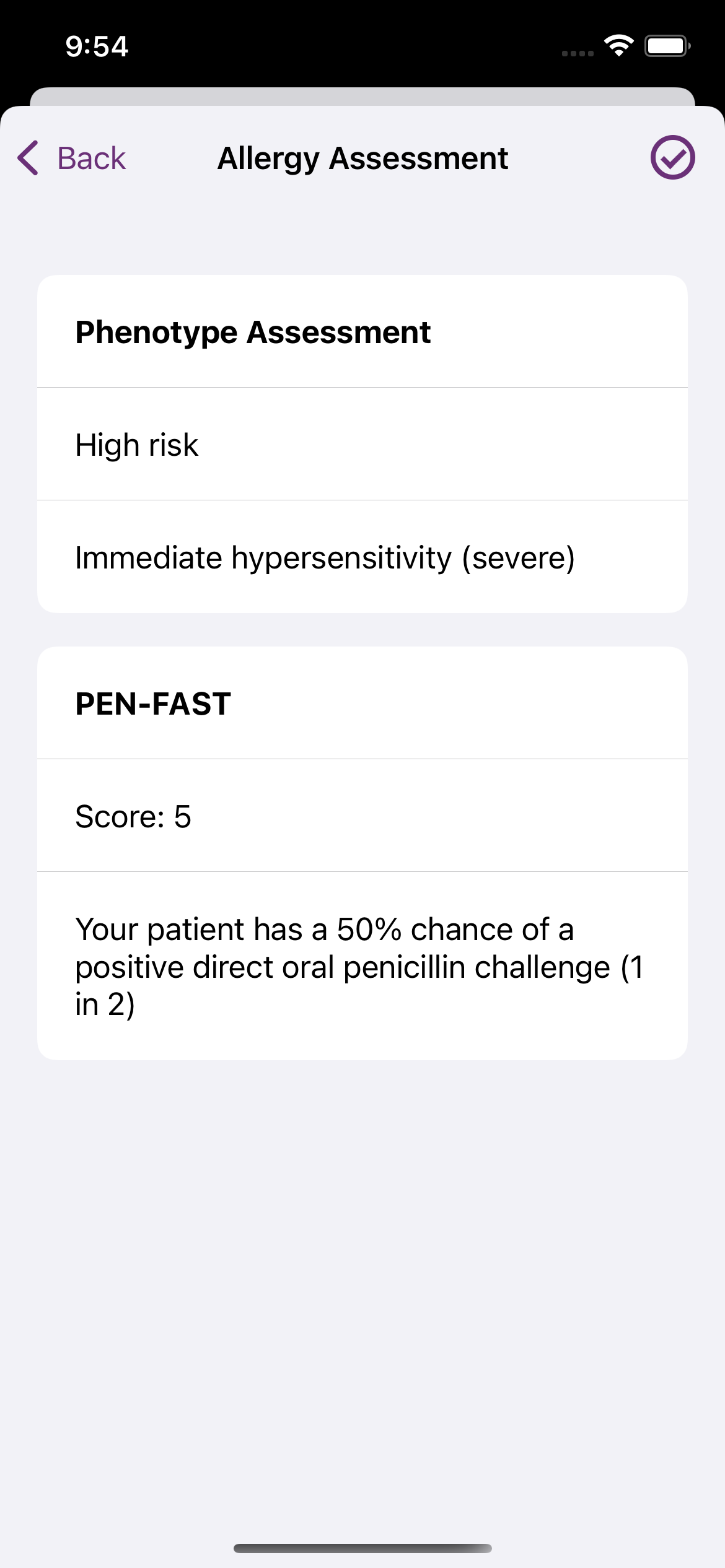 |
| --- | --- | --- |

**Forms:**

After enrolment, investigators will have the ability to complete the following forms:

- Medical history
- Allergy Assessment:
  - Antibiotic Allergy Assessment Tool (see Appendix 1).
  - PEN-FAST score (see Appendix 2).
- Oral Challenge (see Appendix 3) – which automatically assesses eligibility for participation in a direct oral penicillin challenge, in accordance with the criteria in this protocol.

Upon completion of these forms, data are automatically uploaded securely to the REDCap database, hosted by BioGrid.

**Multi-Patient Data Collection:**

Multiple patients can be opened simultaneously by the same user on one device to permit group direct oral penicillin challenge testing. Incomplete forms may be temporarily stored in an encrypted format on the device for up to three hours, after which, they are automatically deleted if not completed and uploaded to REDCap.

**Additional Information:**

- Authenticated smartphone app users have write-only access and can upload data to REDCap to facilitate data collection but cannot access previously collected patient data from REDCap.
- Users can only upload data to clinical sites that they are associated with and are unable to upload data from other sites.
- All data are transmitted to REDCap securely, and once submitted, no patient data are stored on the user’s device.
- The smartphone app IP is owned by the chief investigator.
- The smartphone app does not provide advice regarding specific allergy testing, nor does it provide instruction on how to perform allergy testing.

| 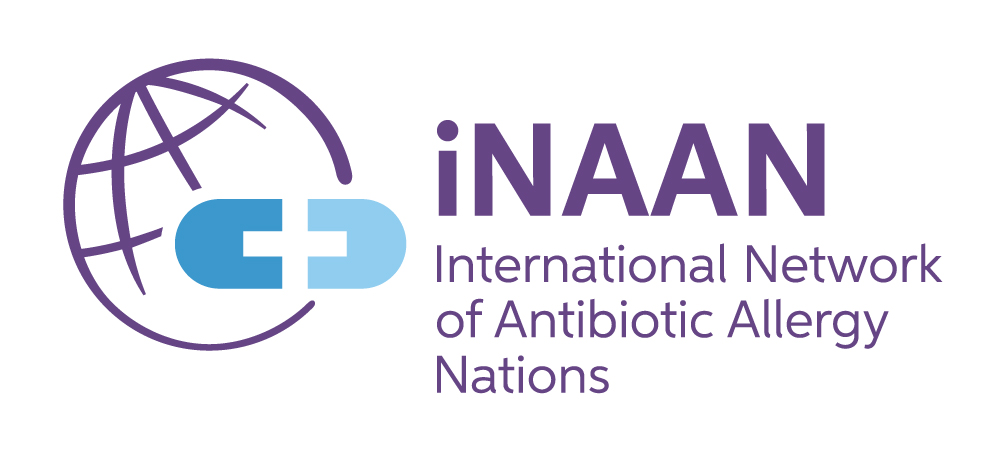 |
| --- |
| International Network of Antibiotic Allergy Nations (iNAAN) |
| Appendix V: REDCap Case Report Form |
| Version: 2.0  Date: 28 March 2023 |


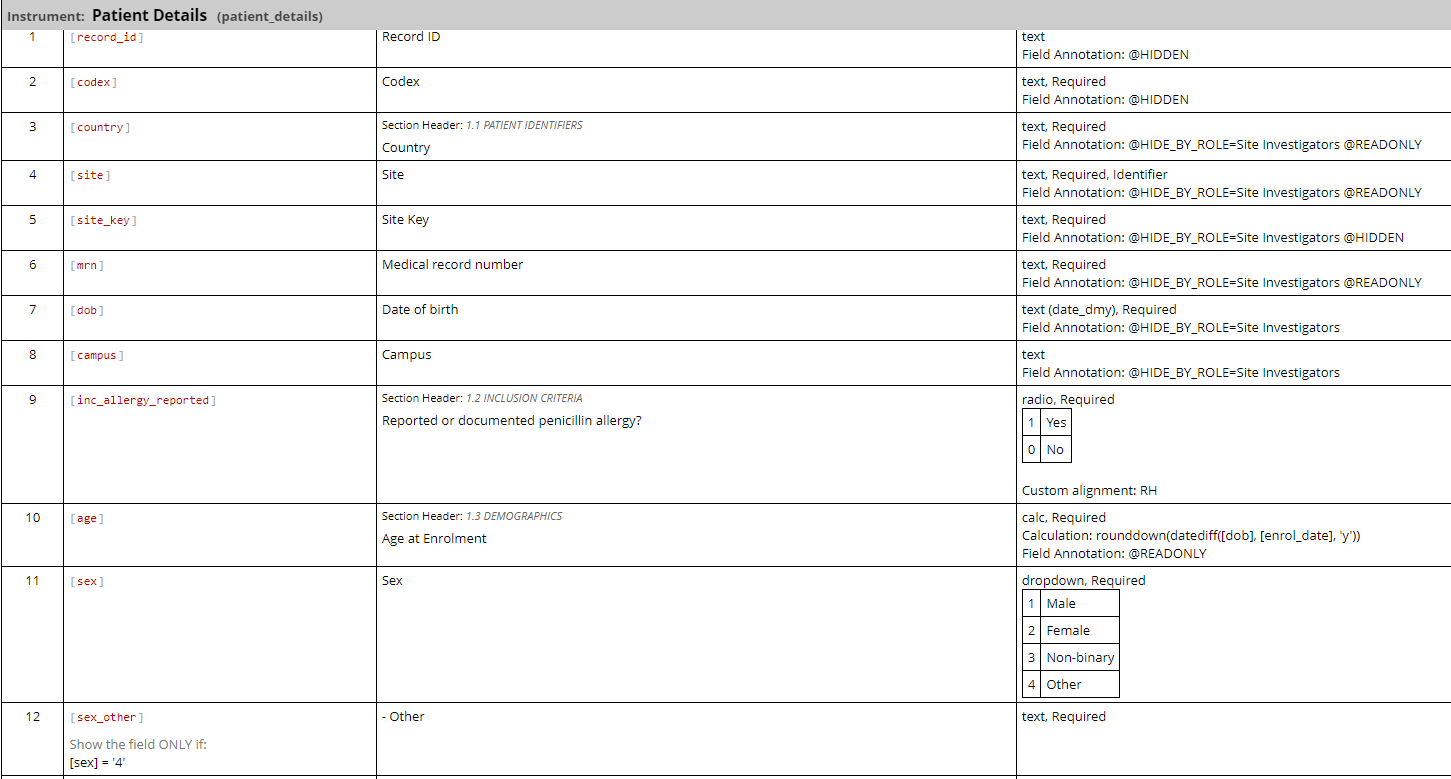


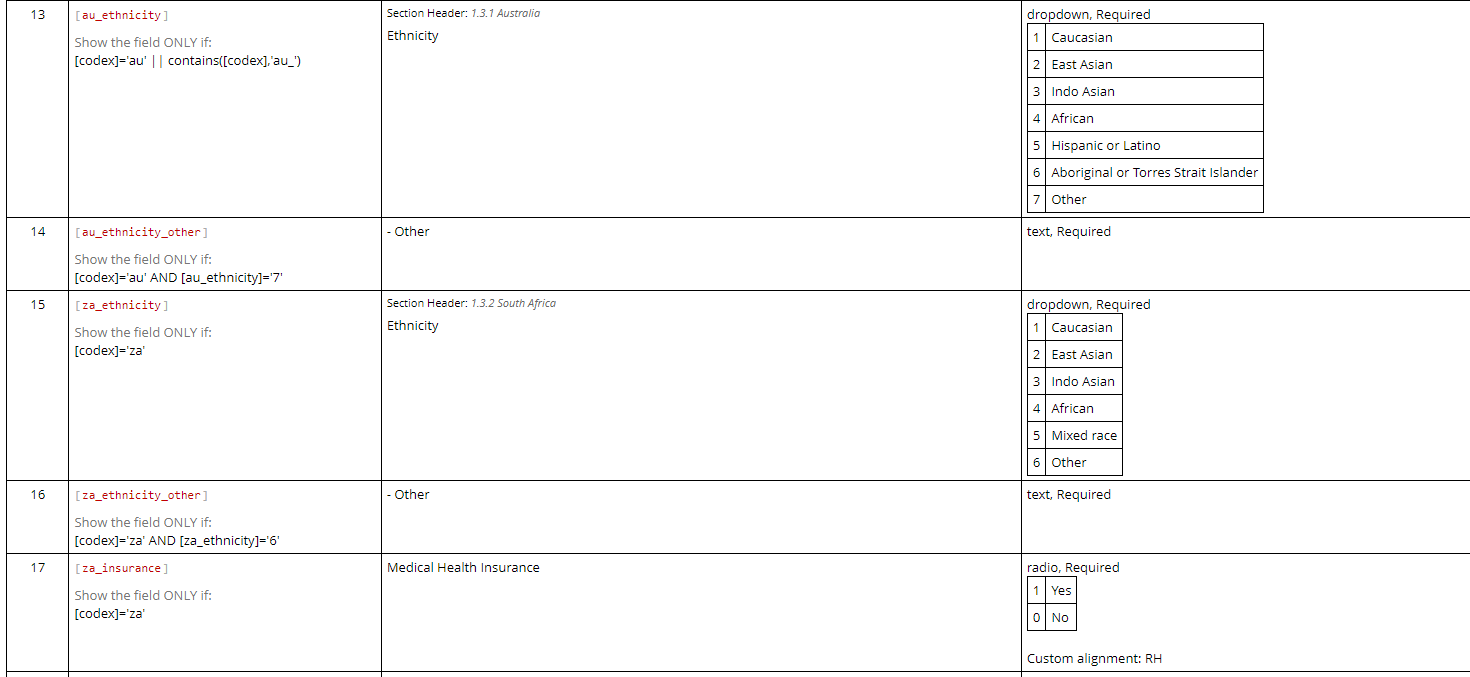


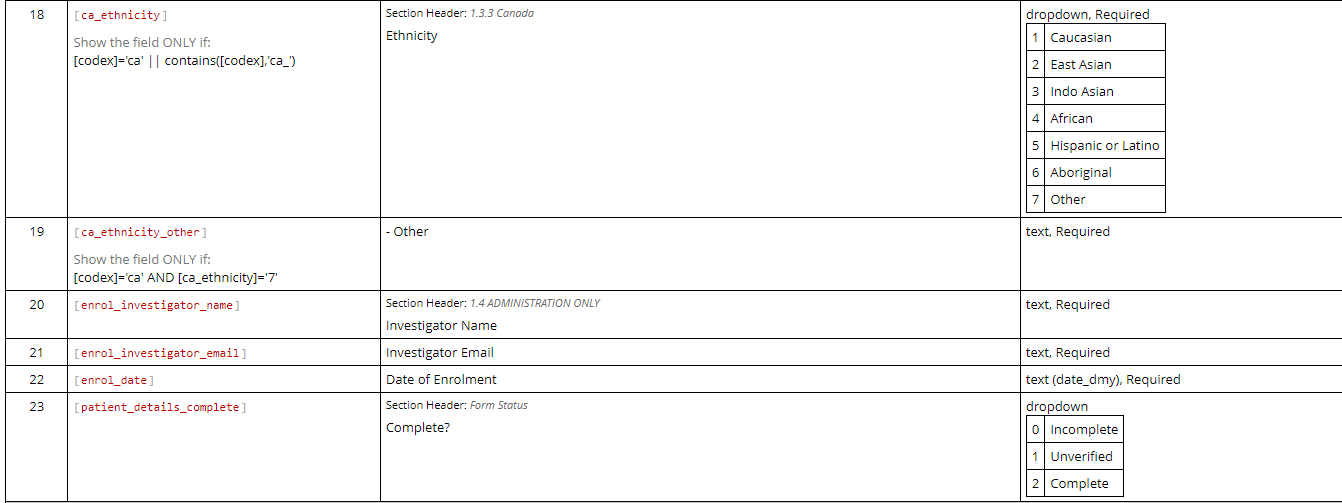


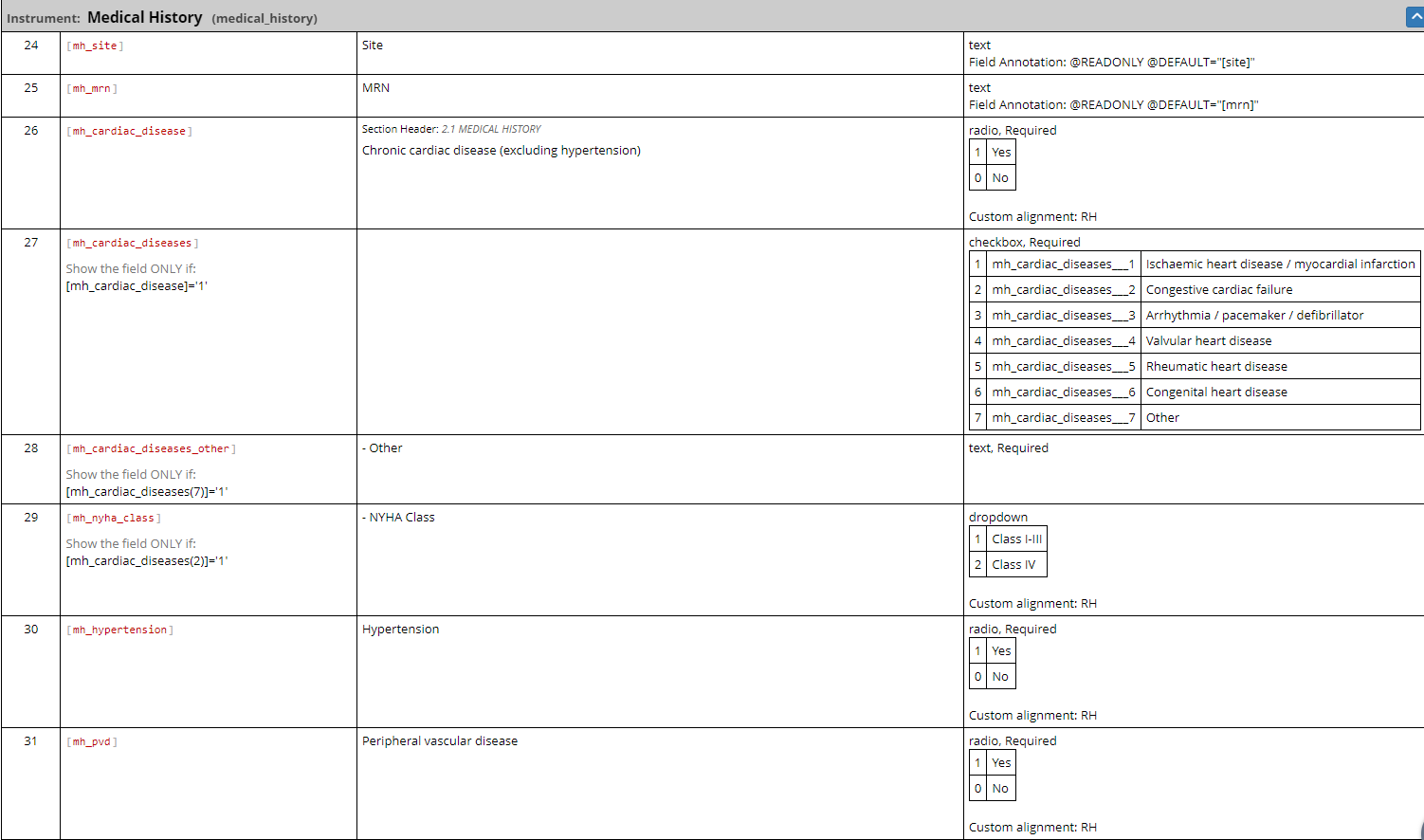


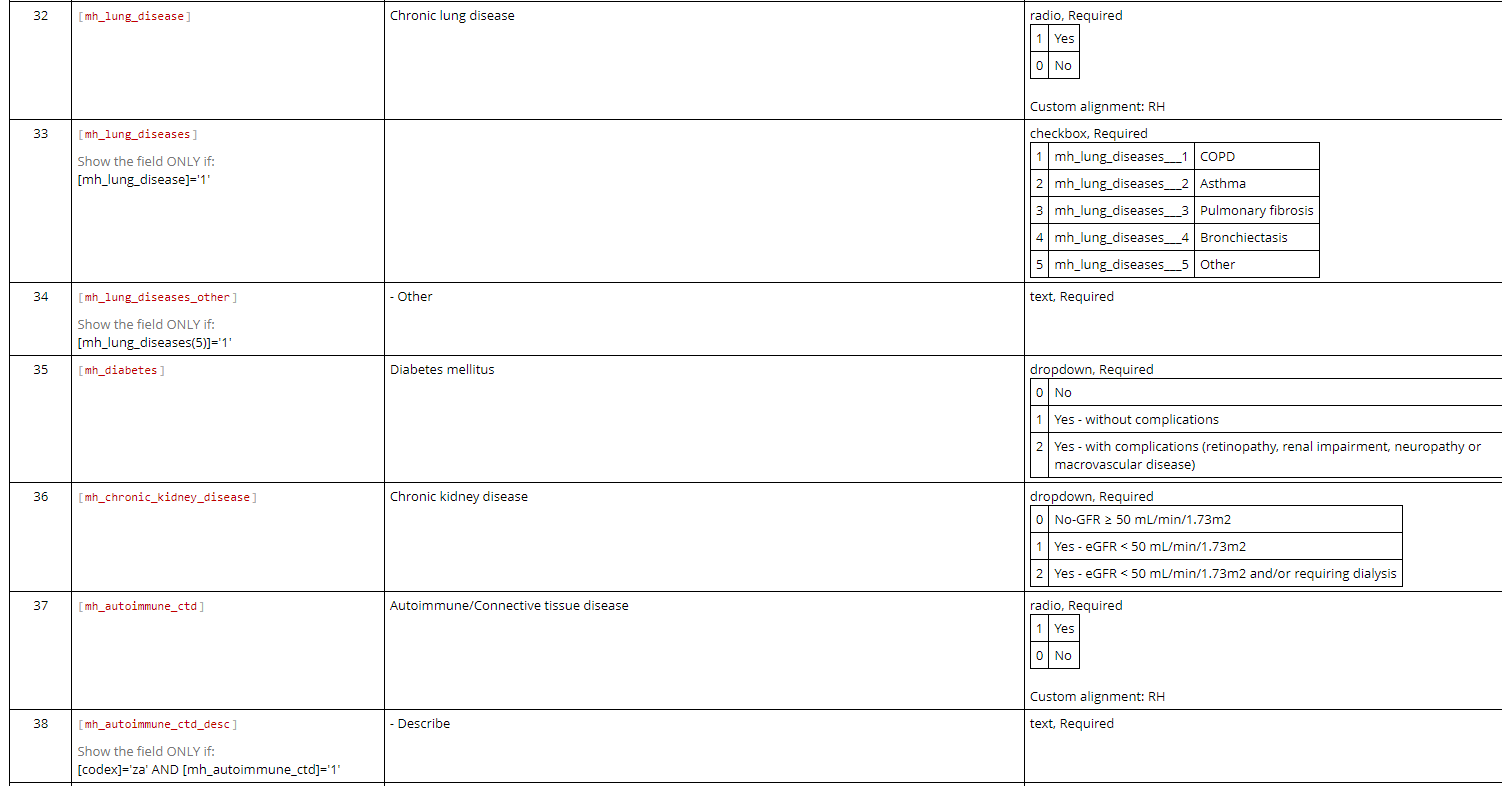


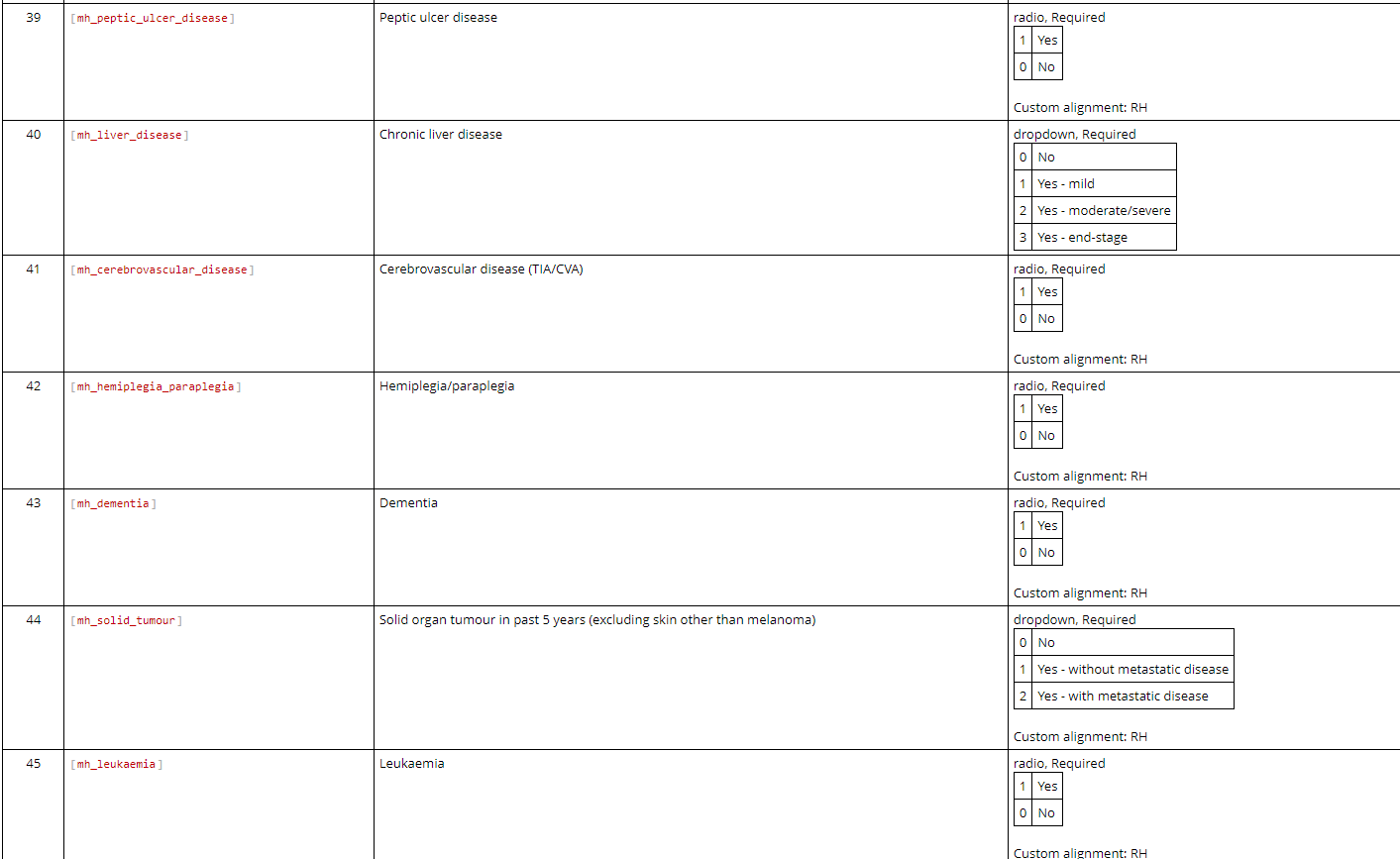


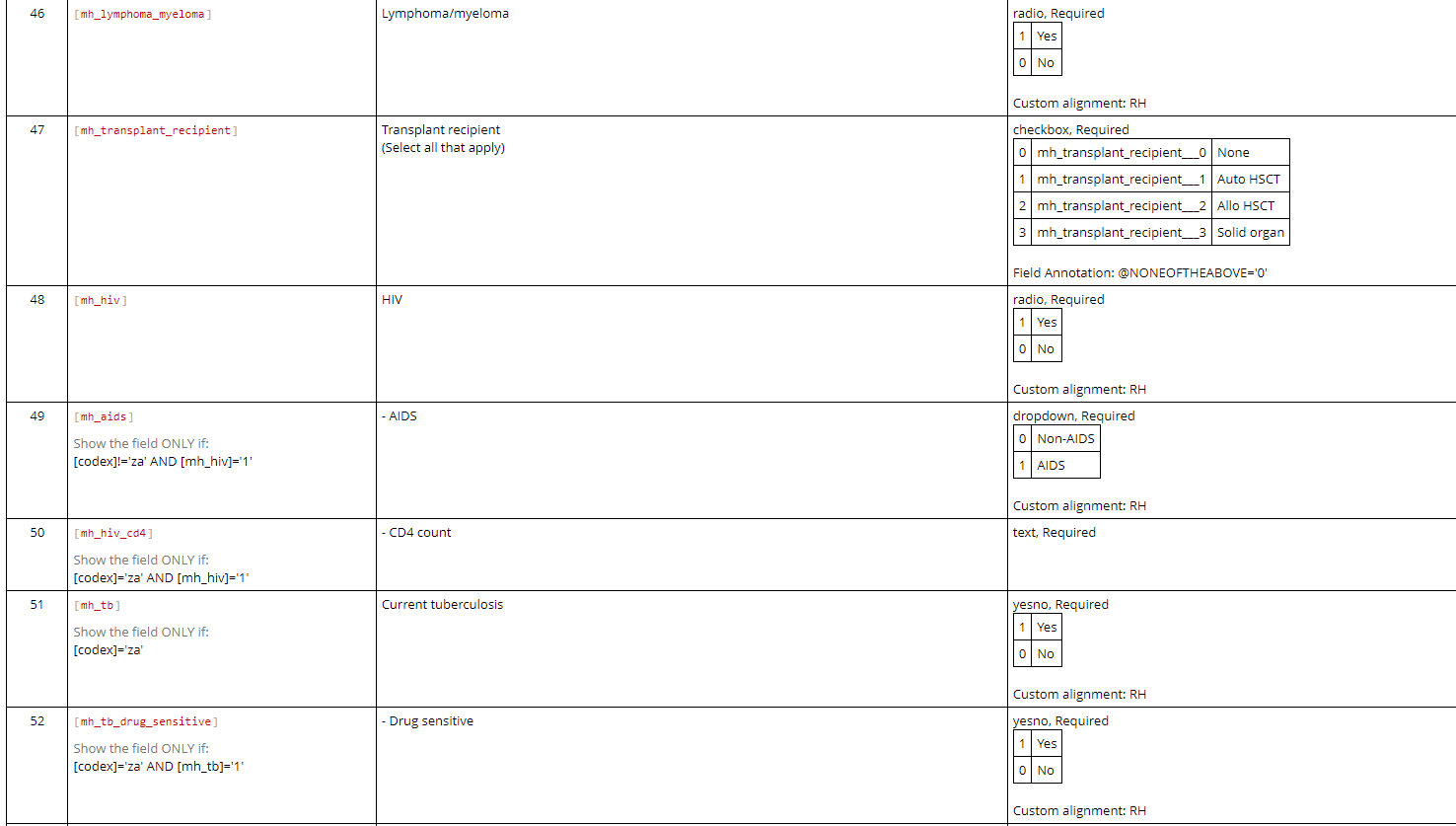


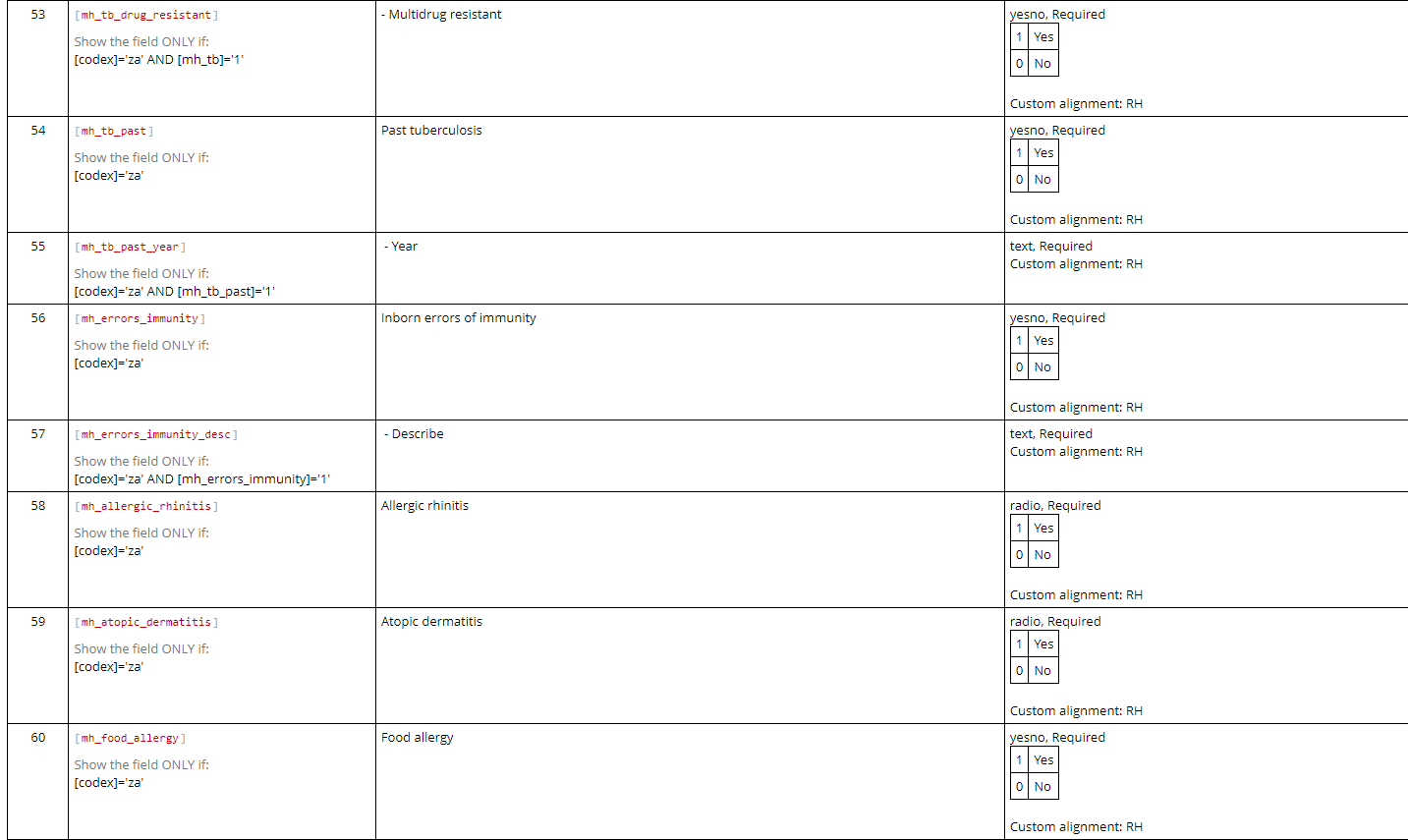


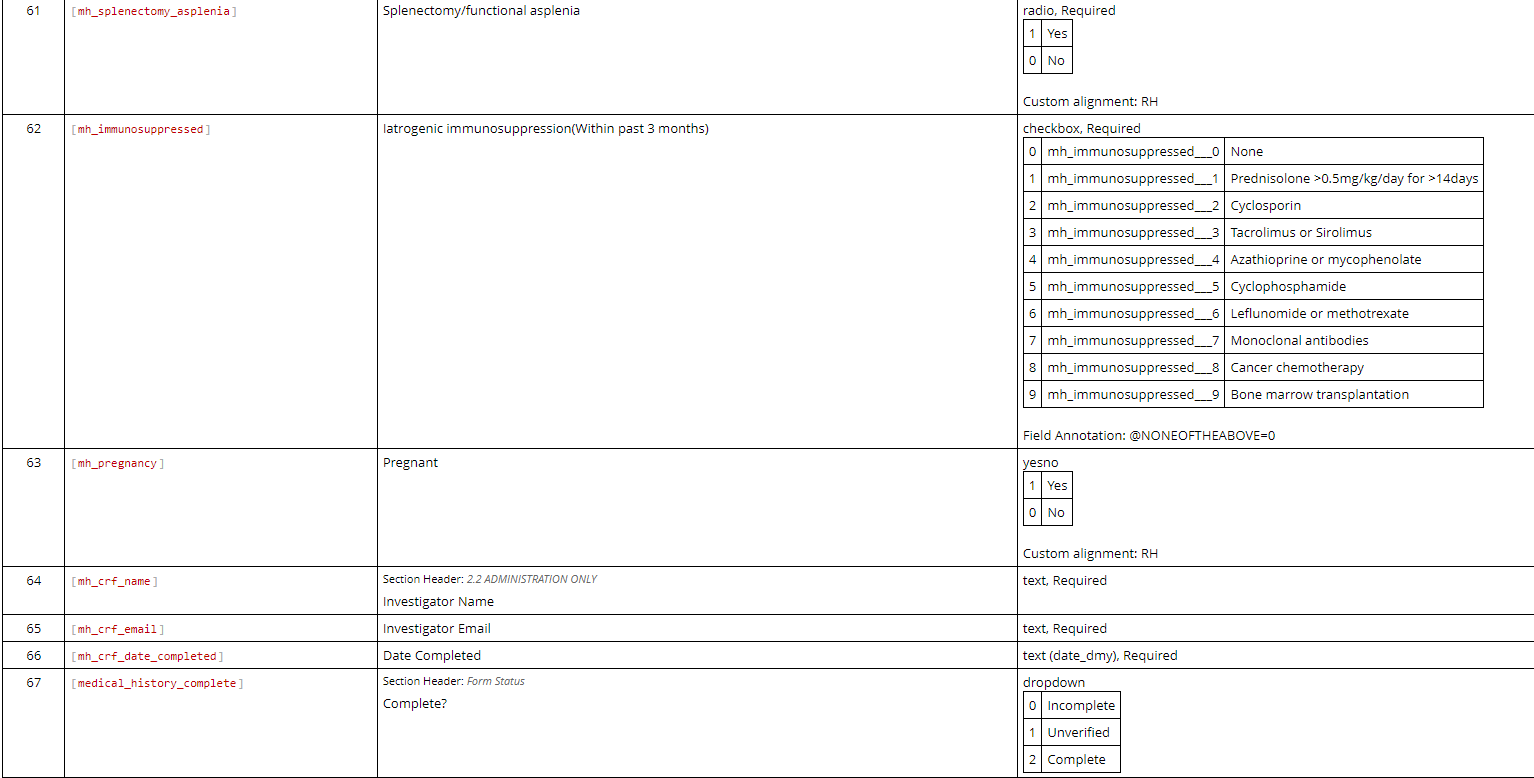


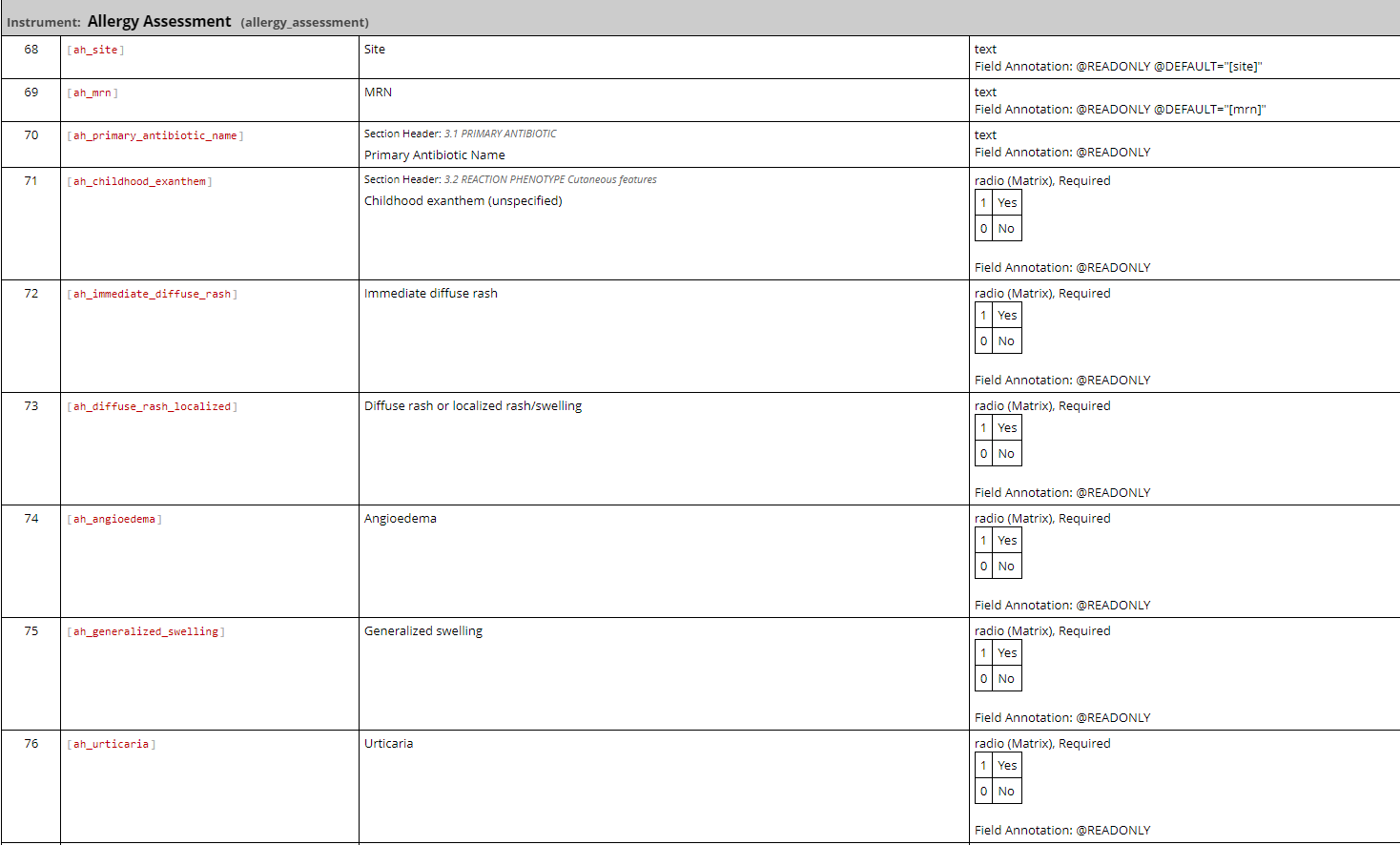


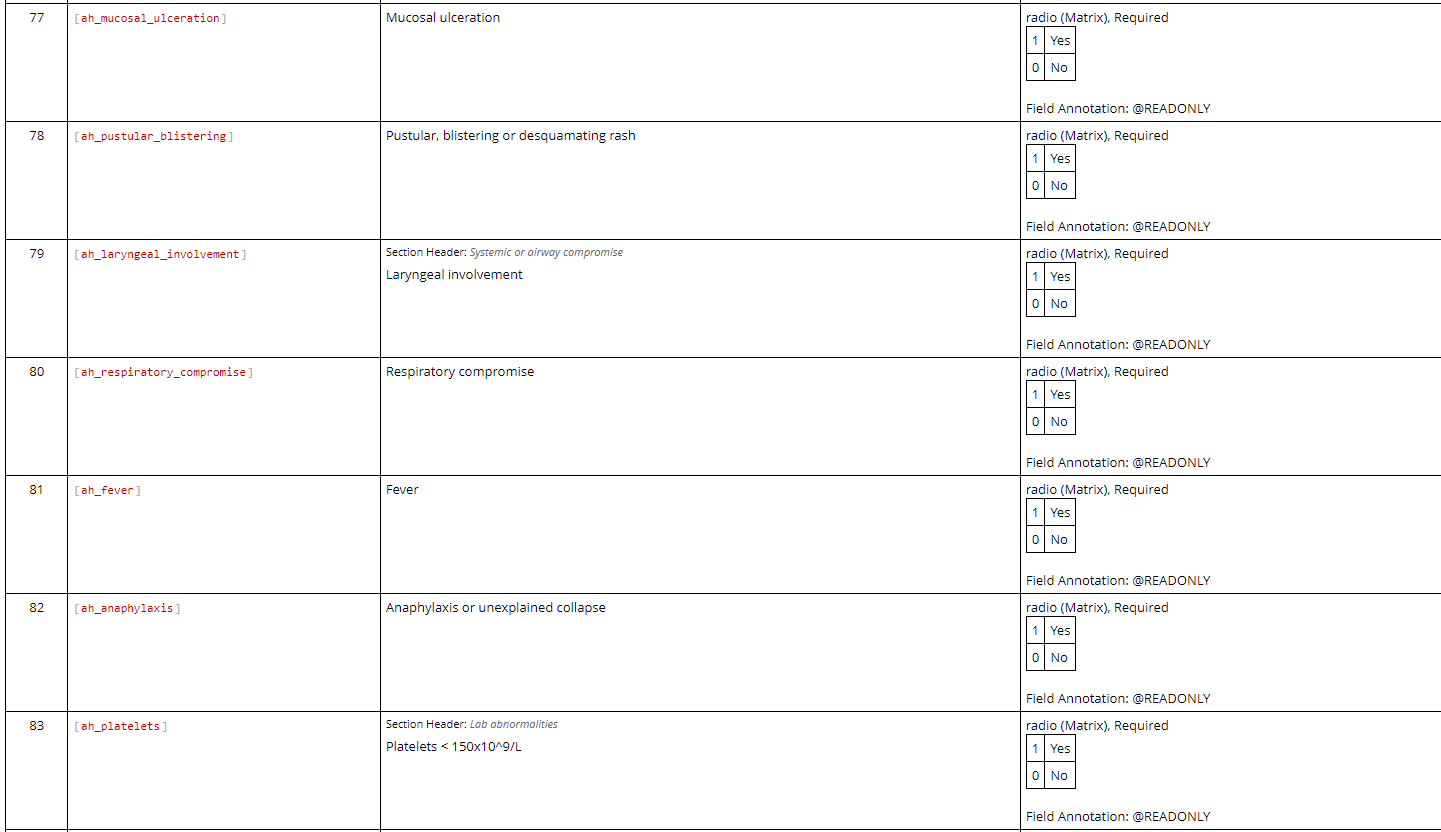


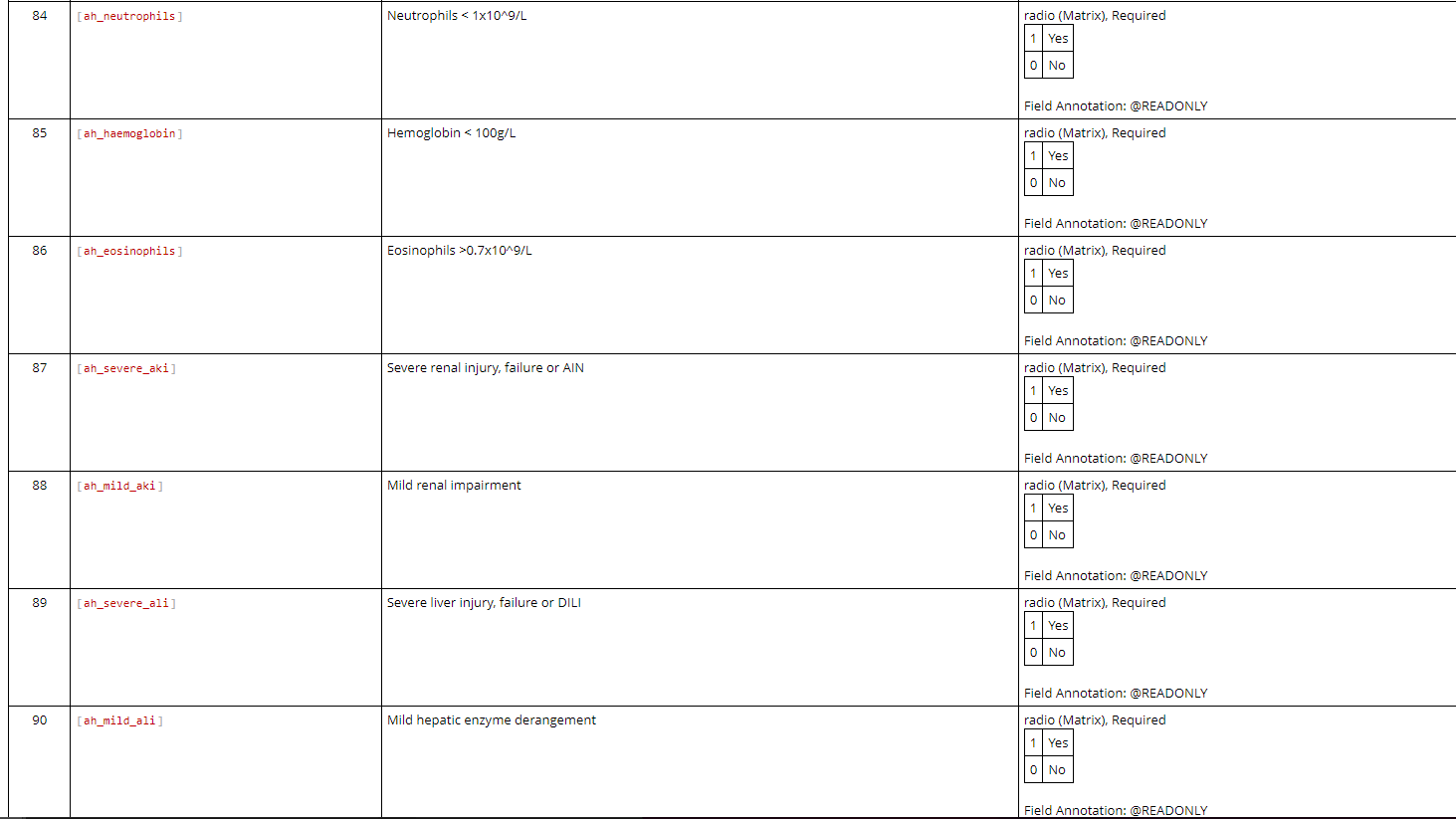


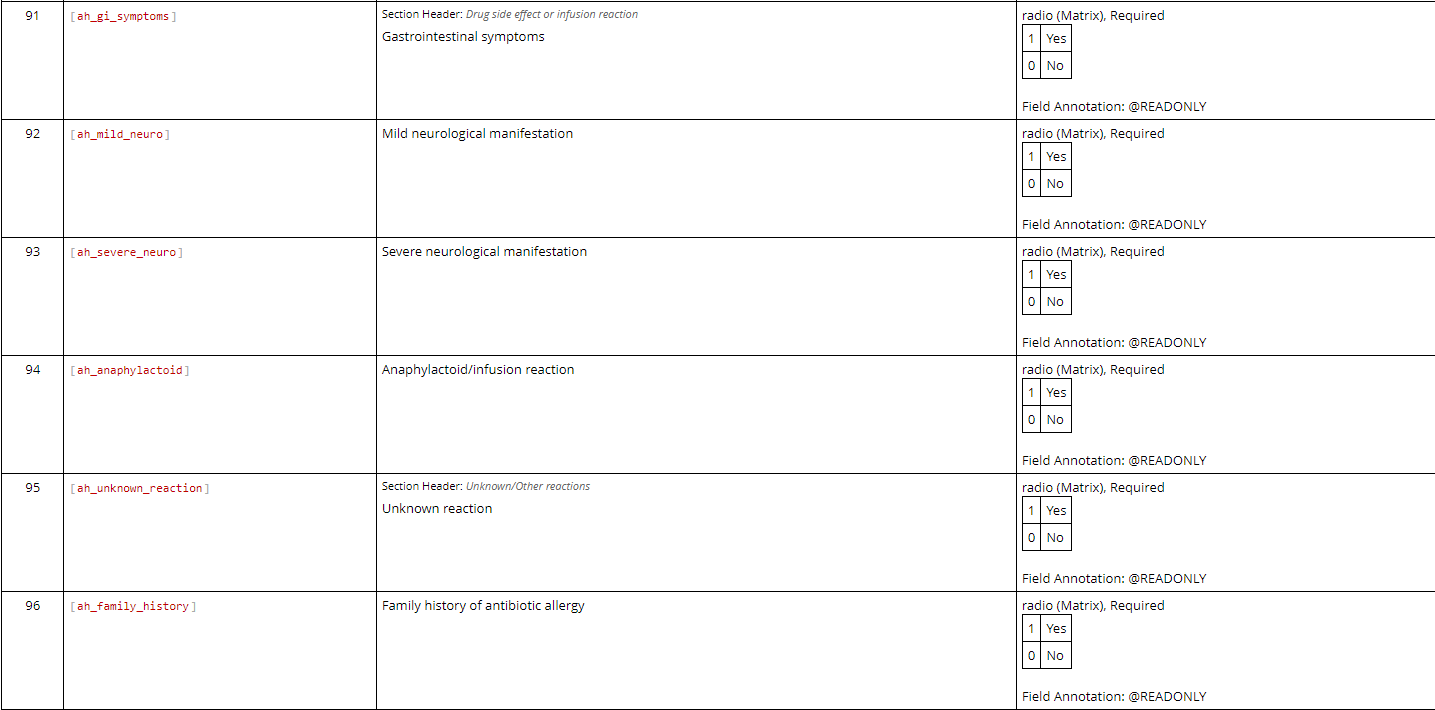


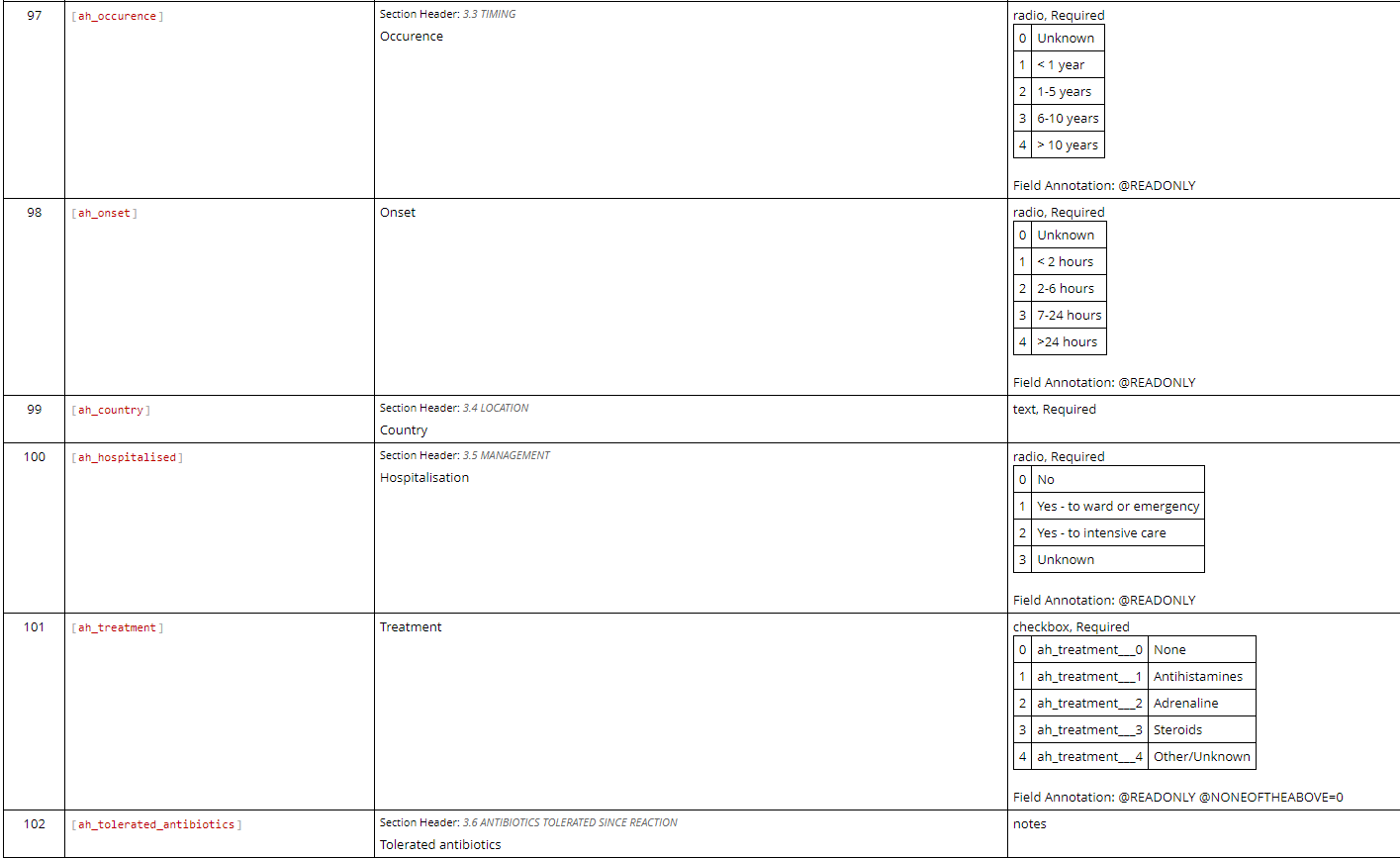


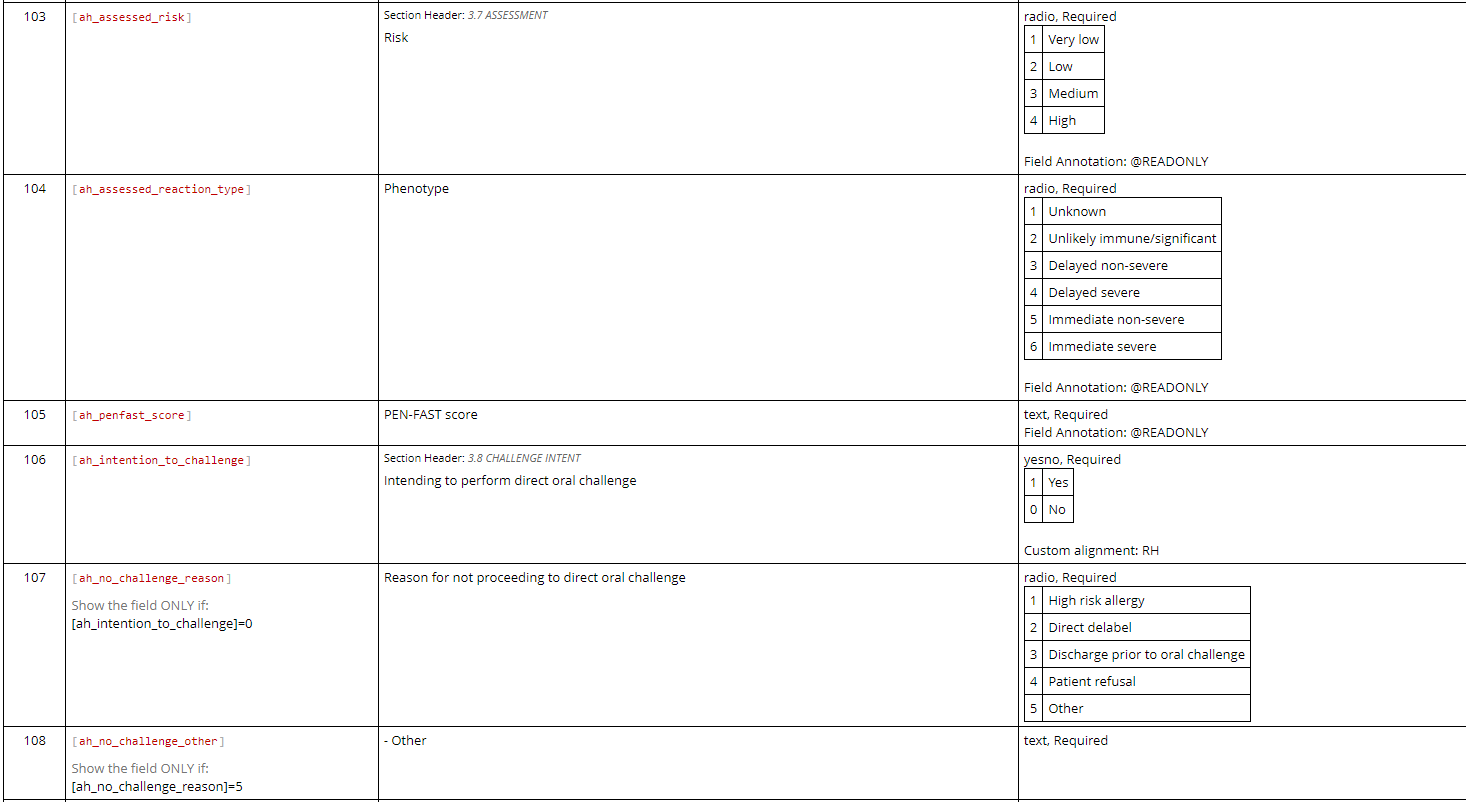


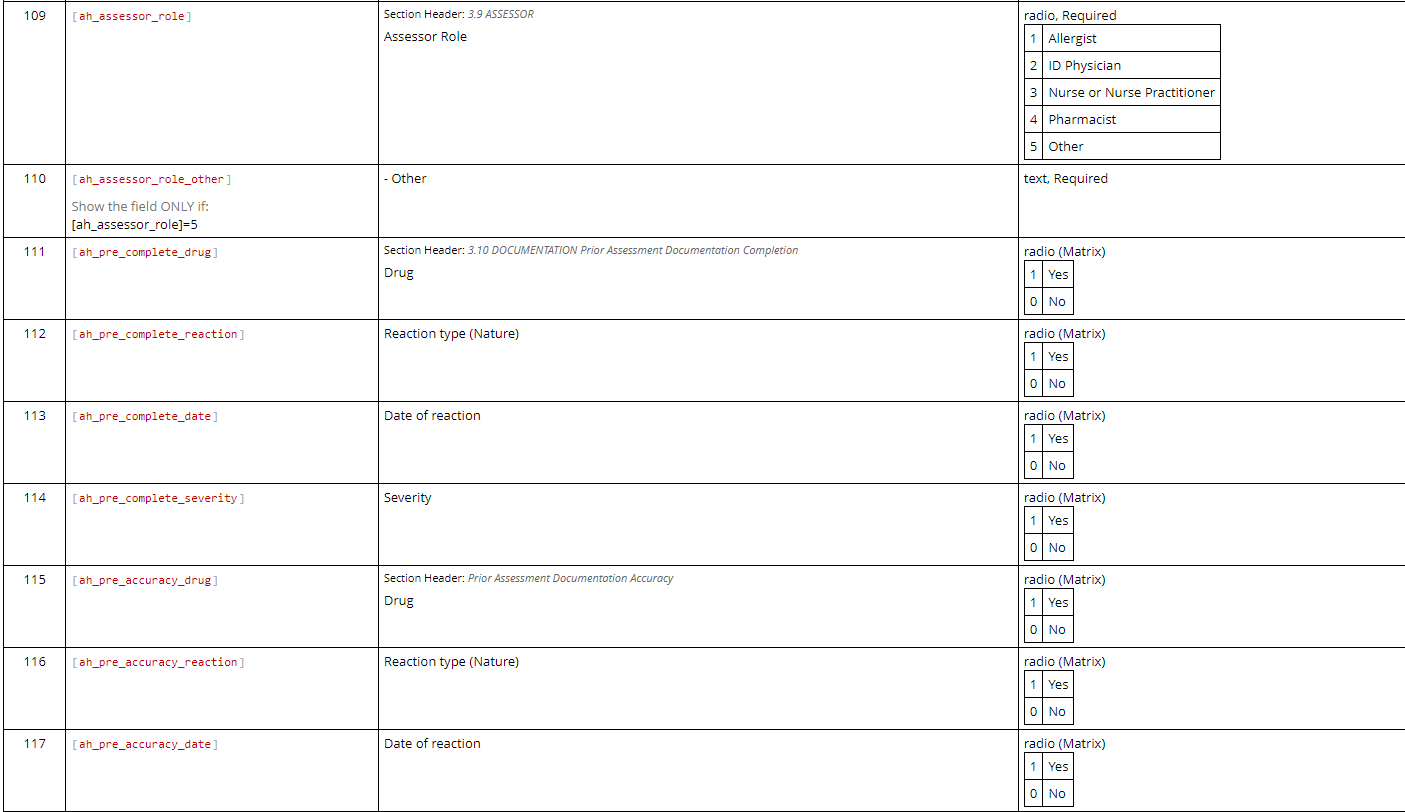


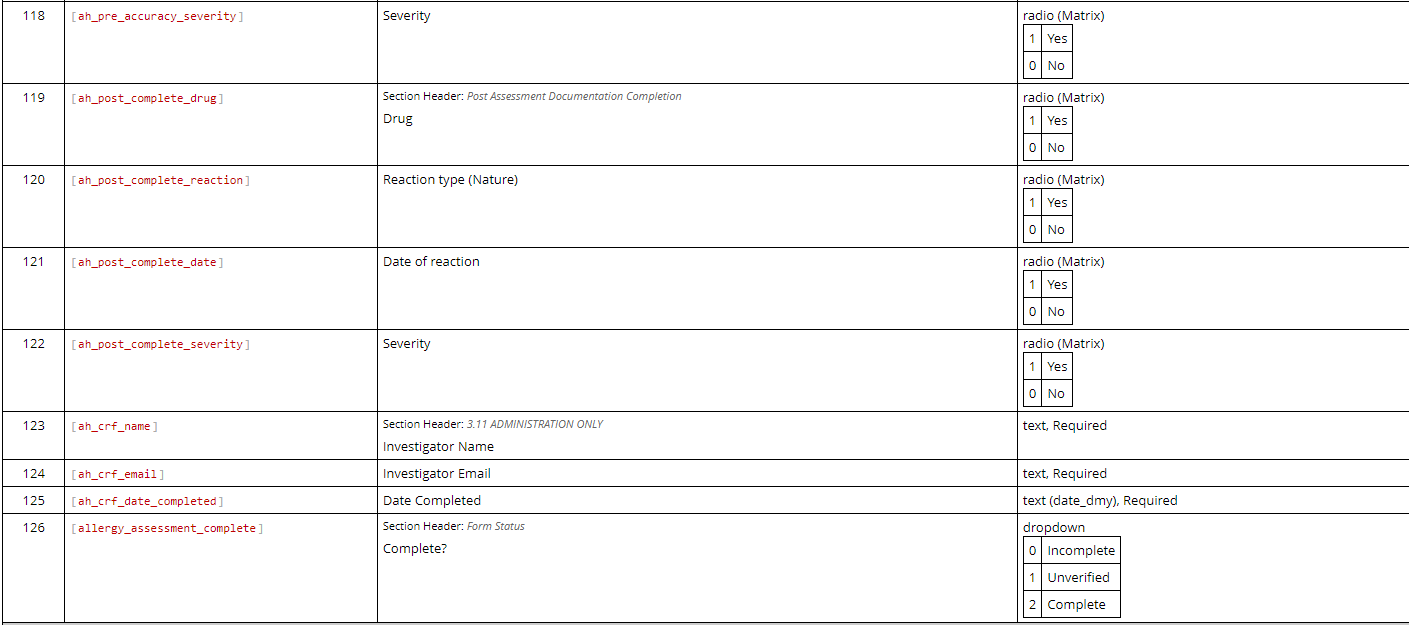


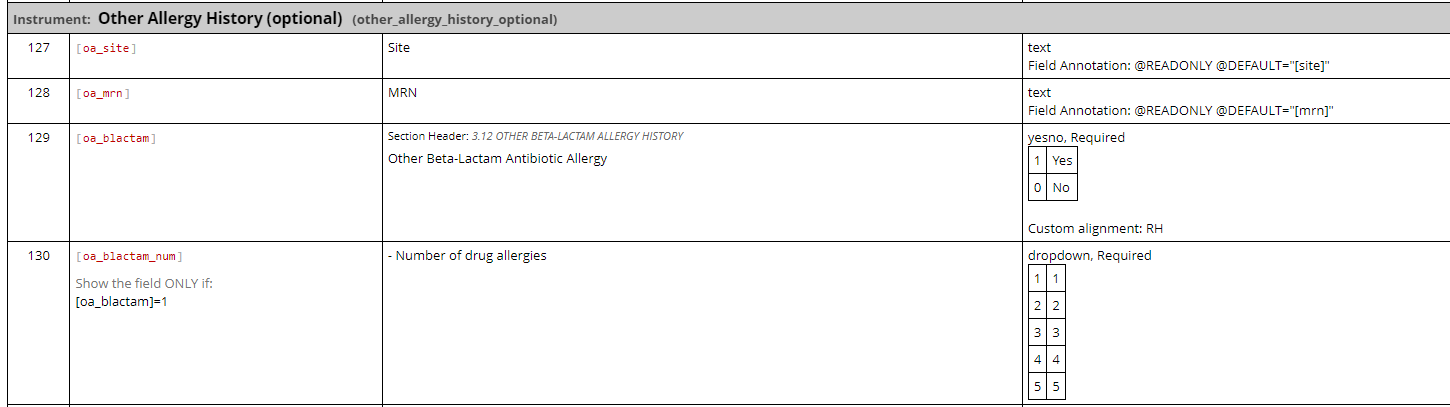


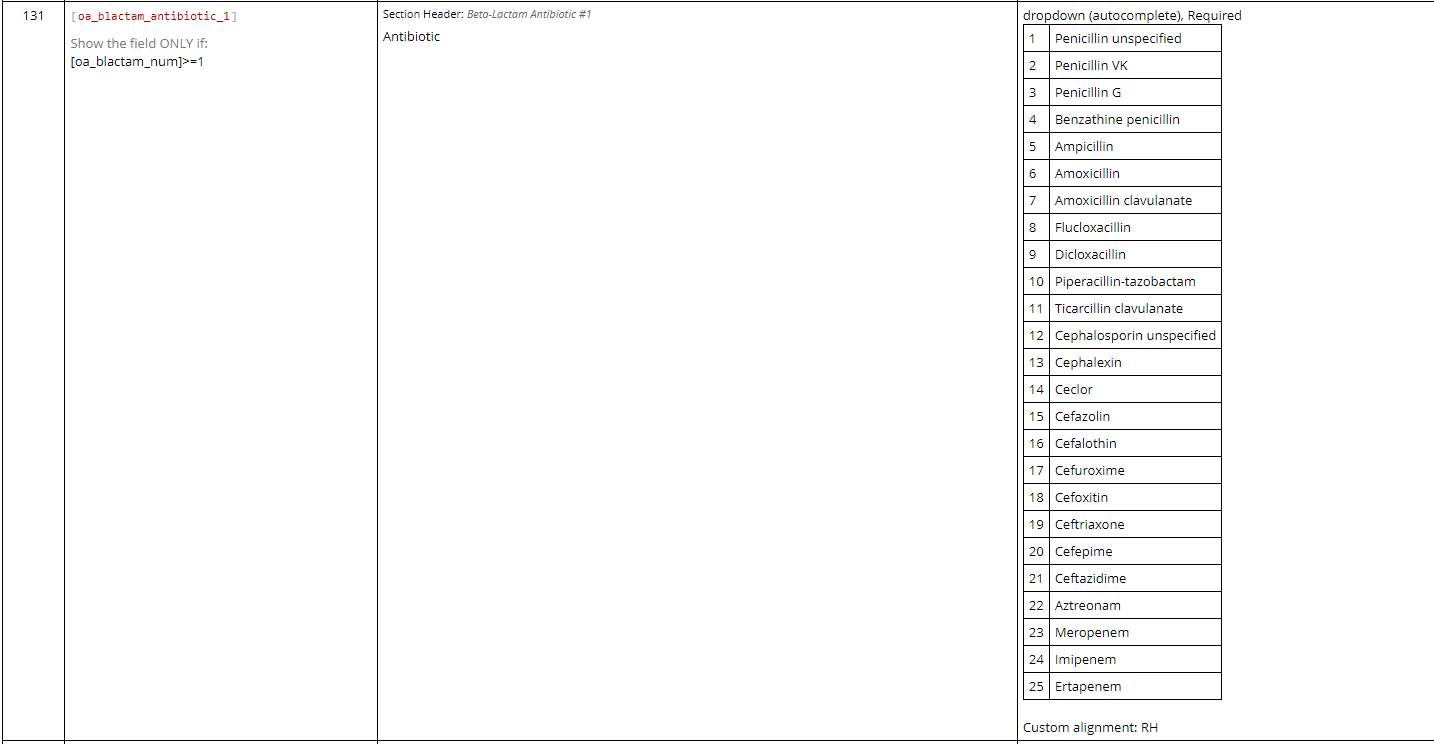


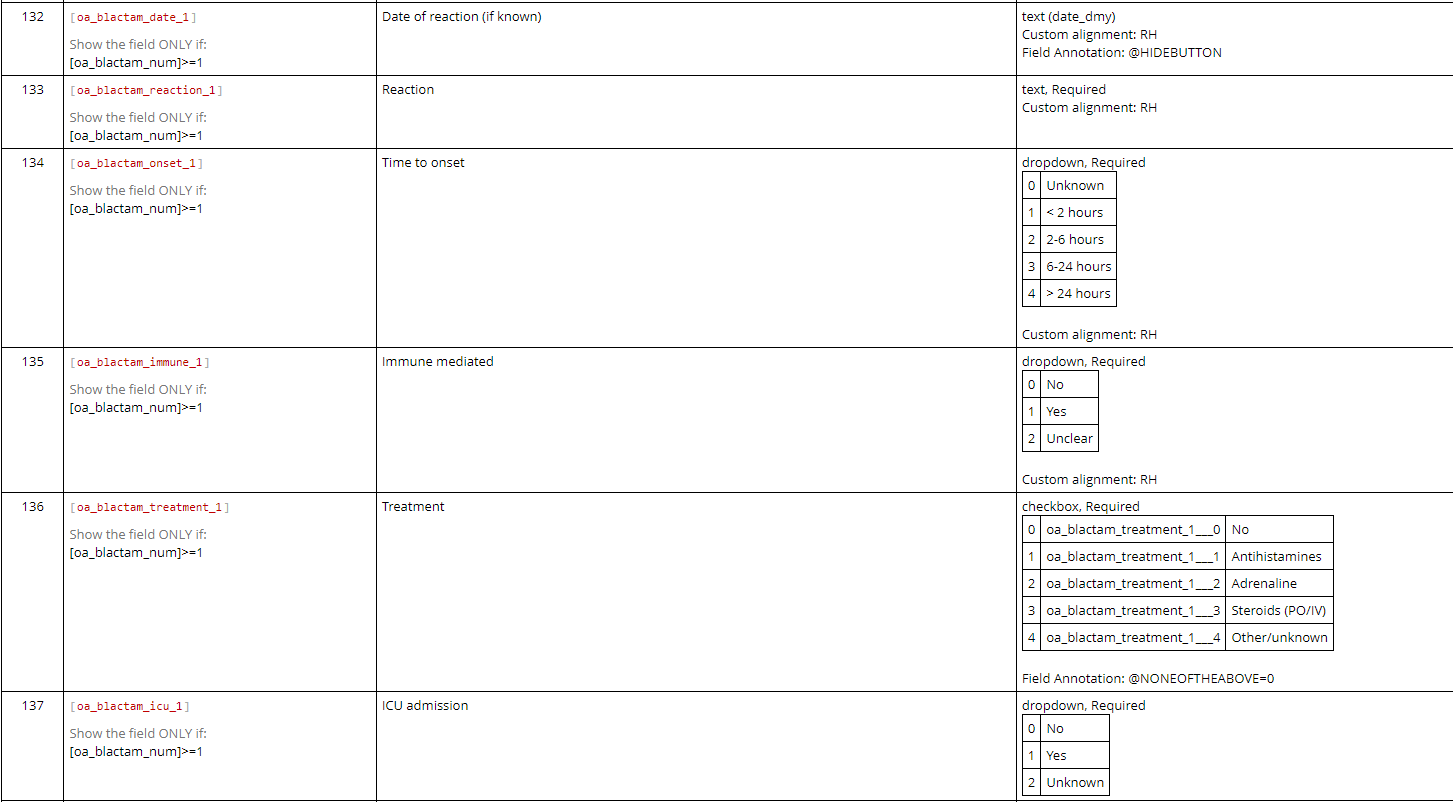


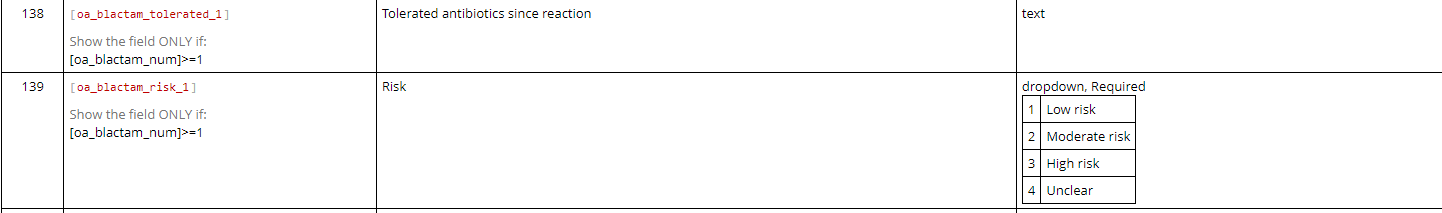


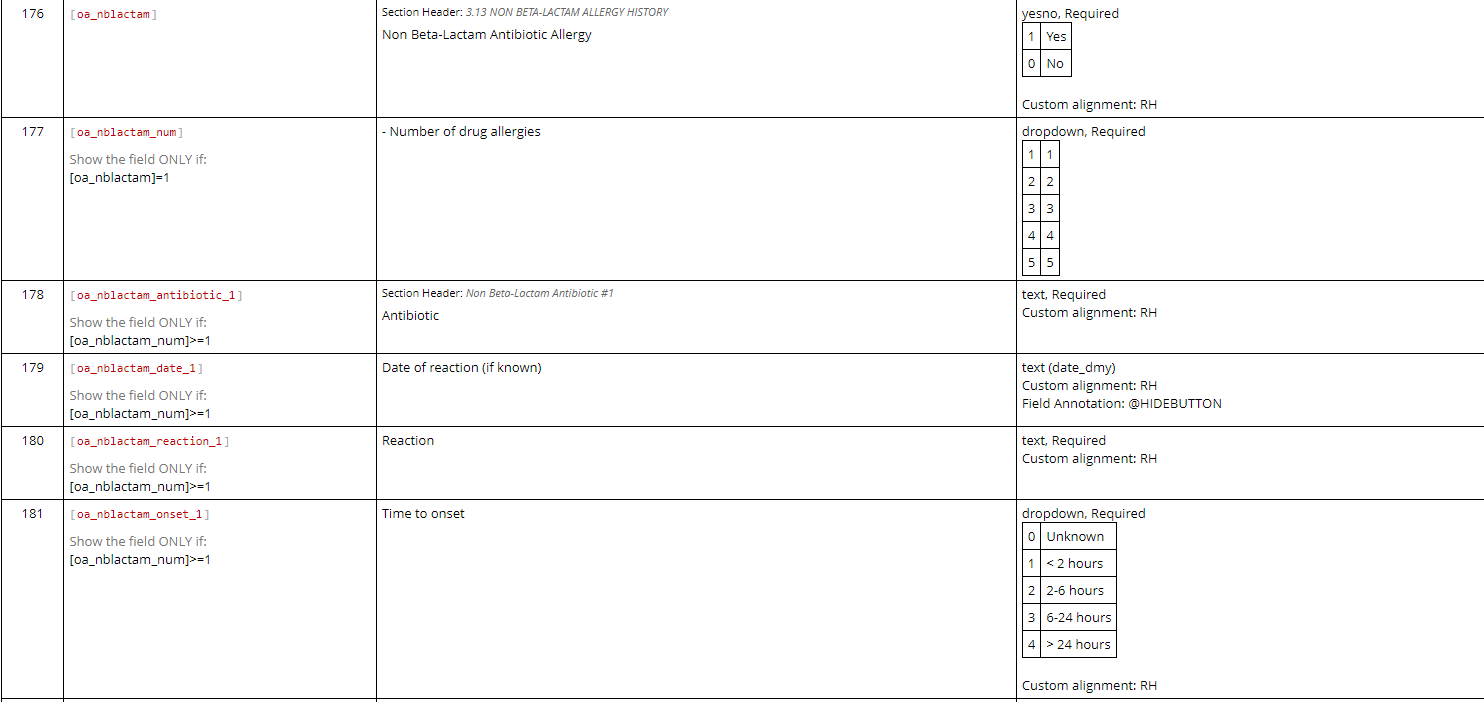


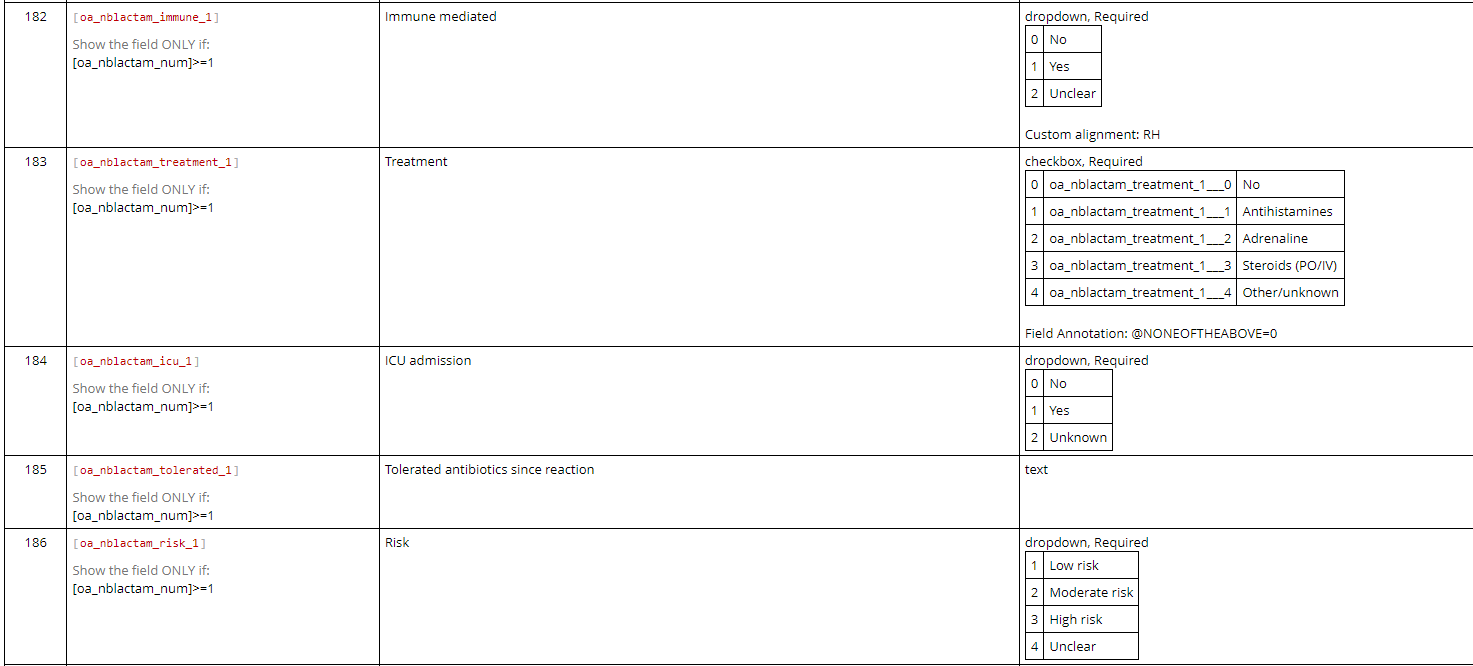


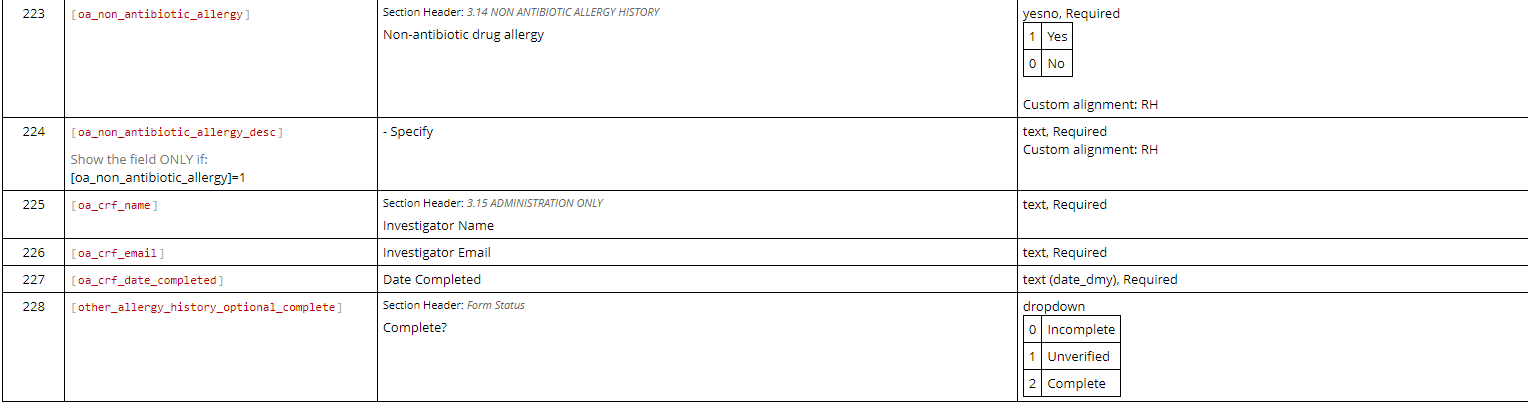


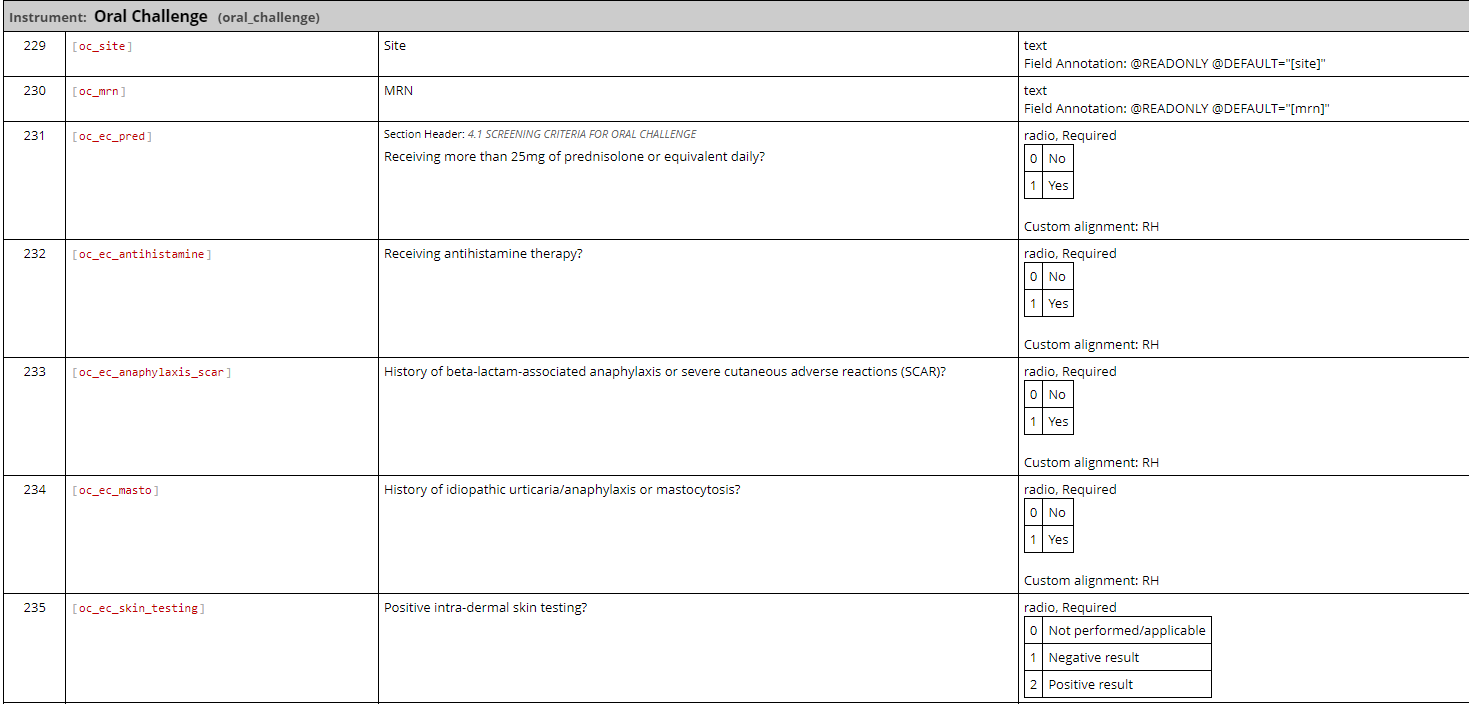


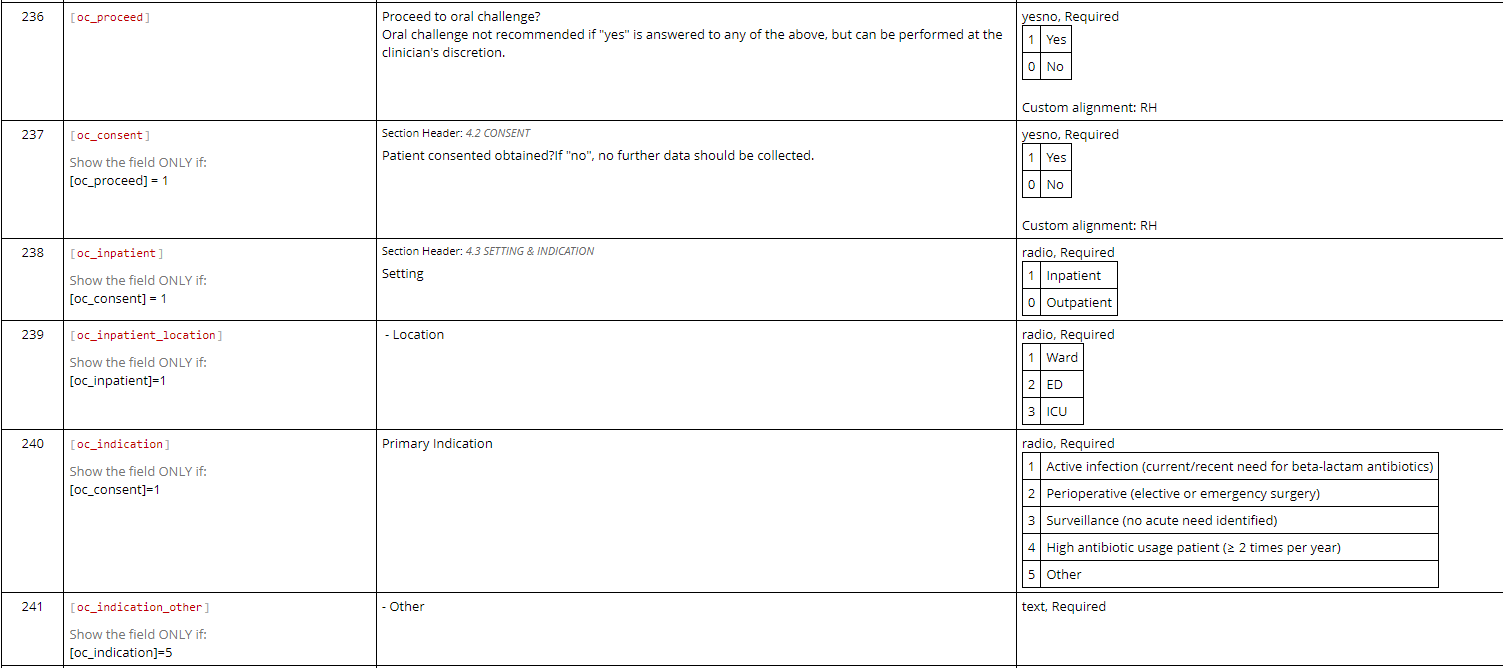


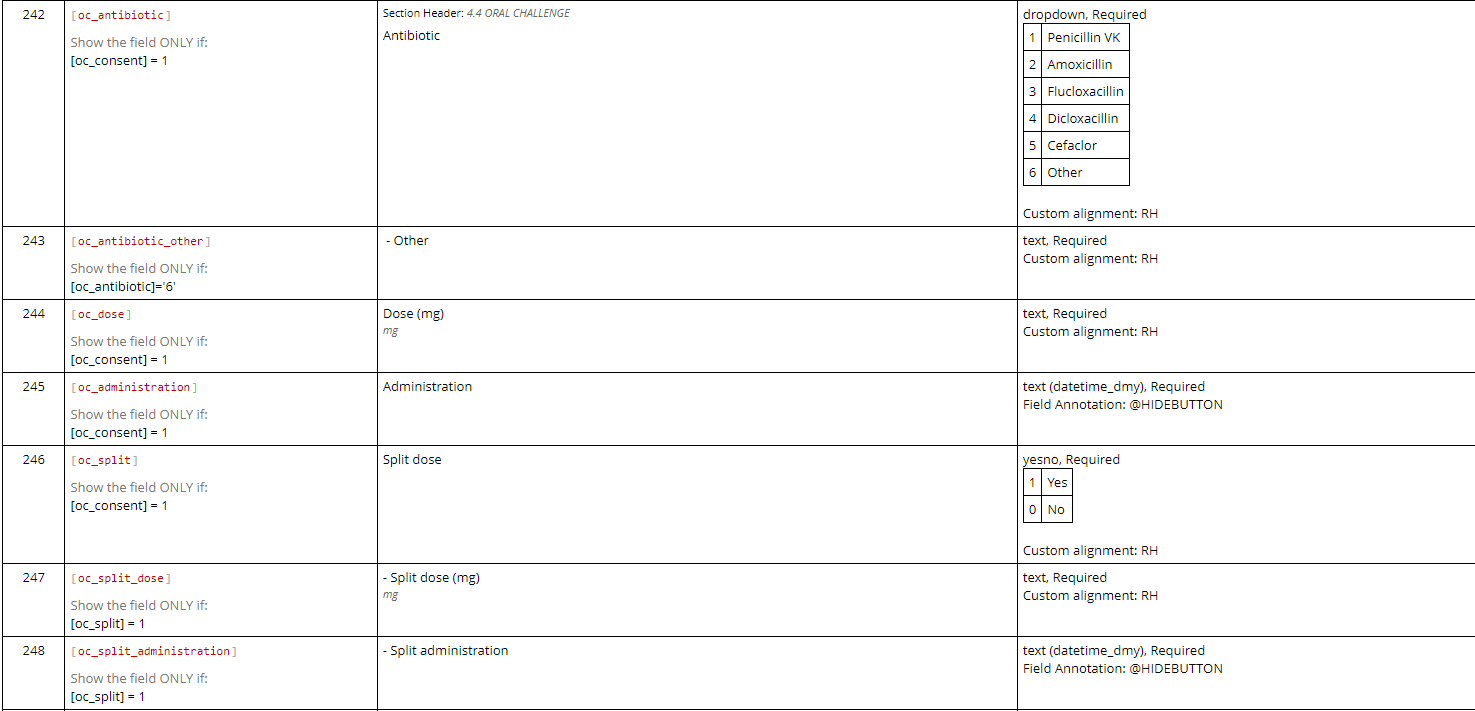


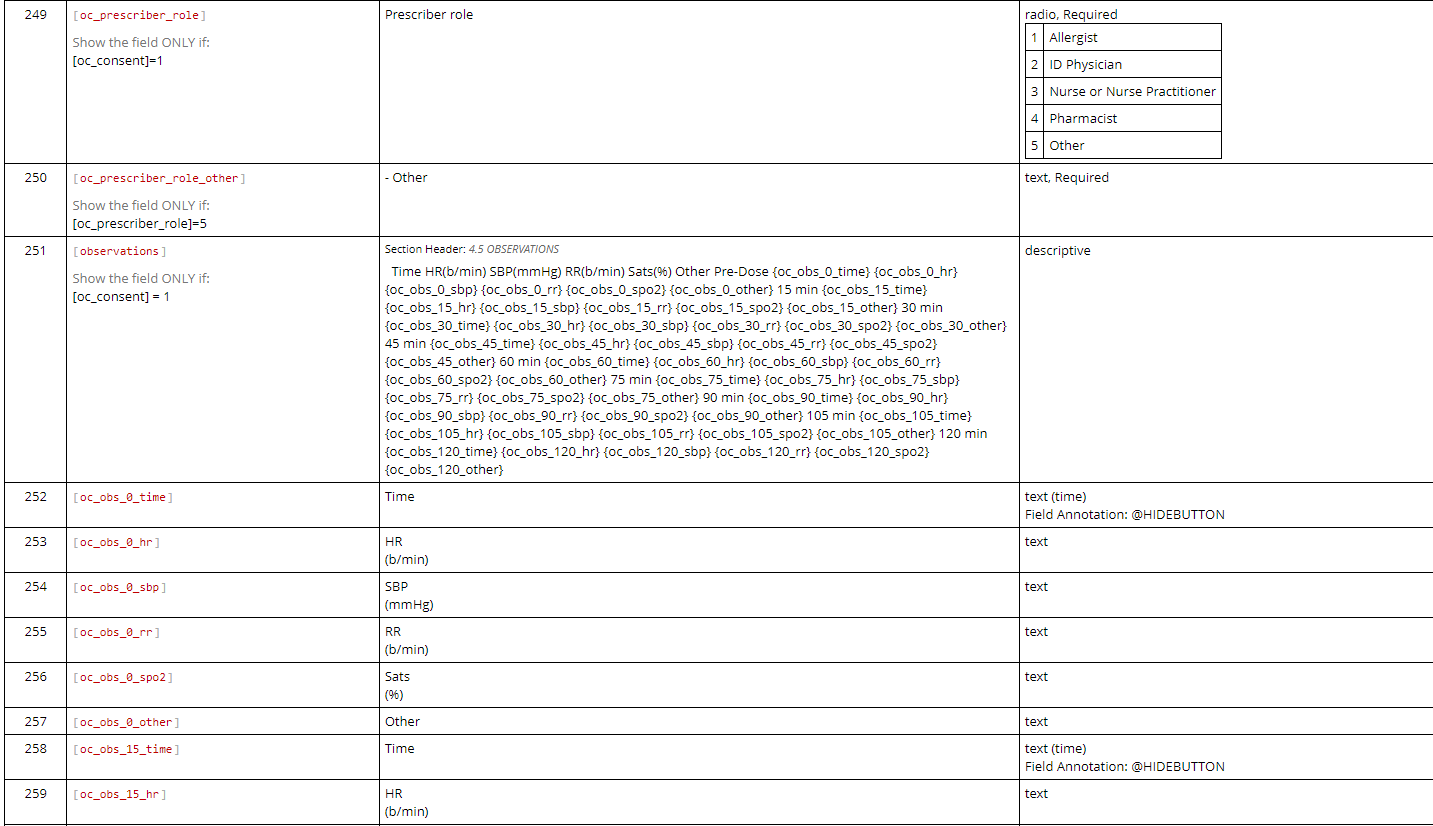


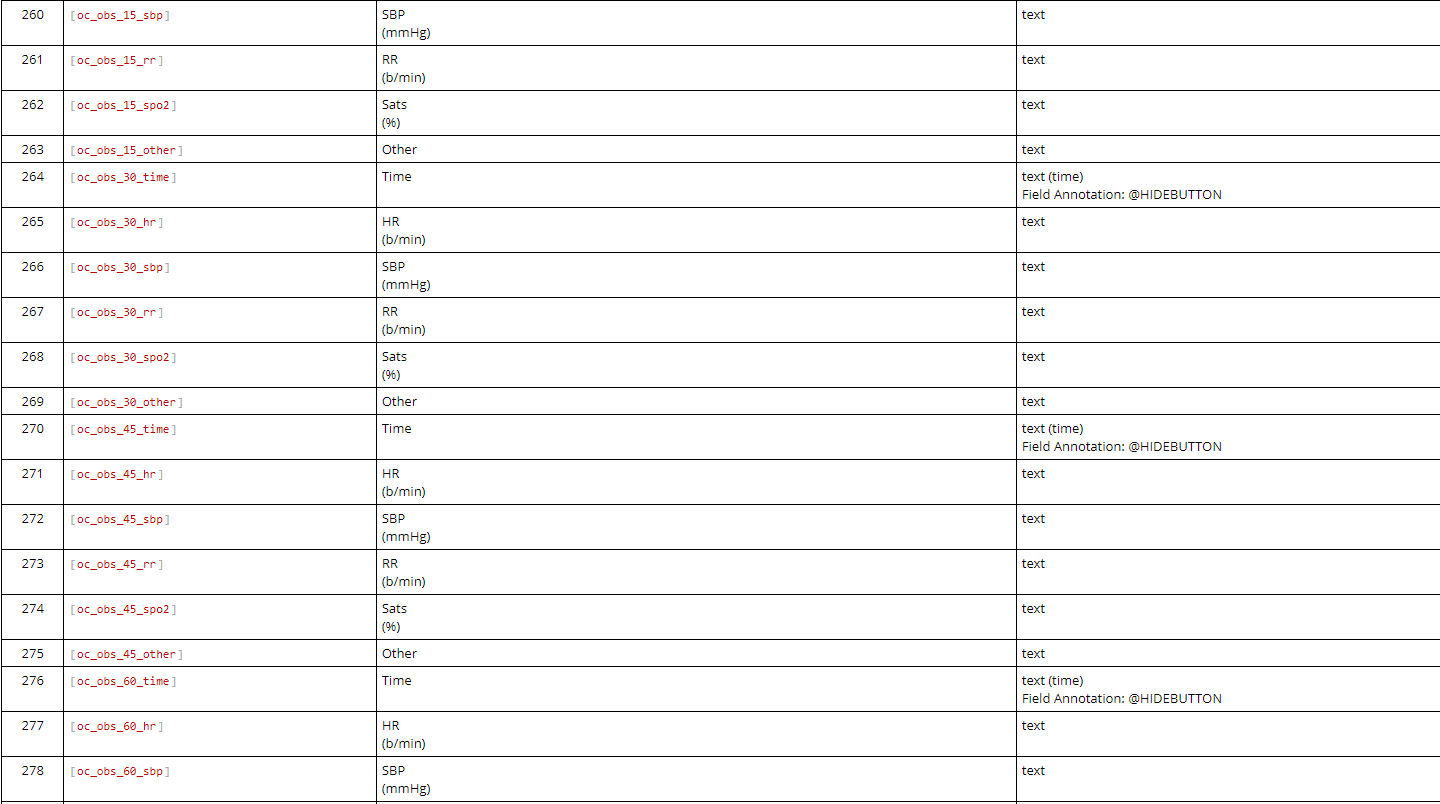

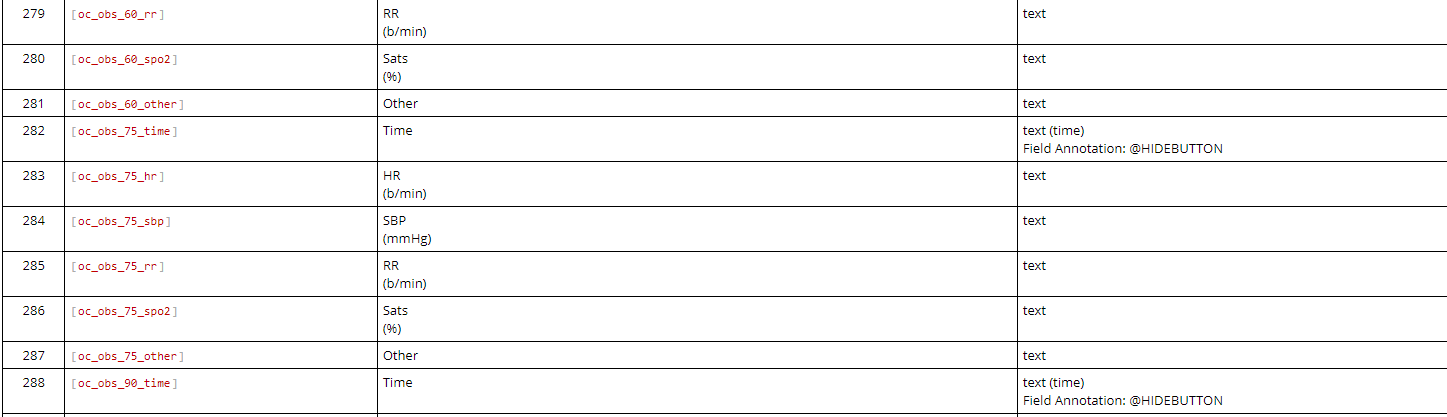


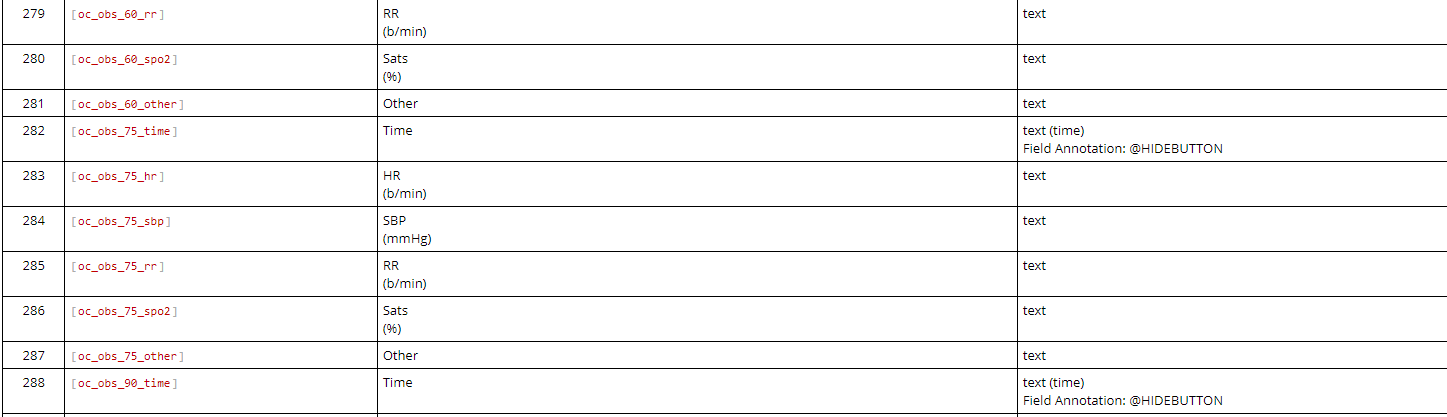


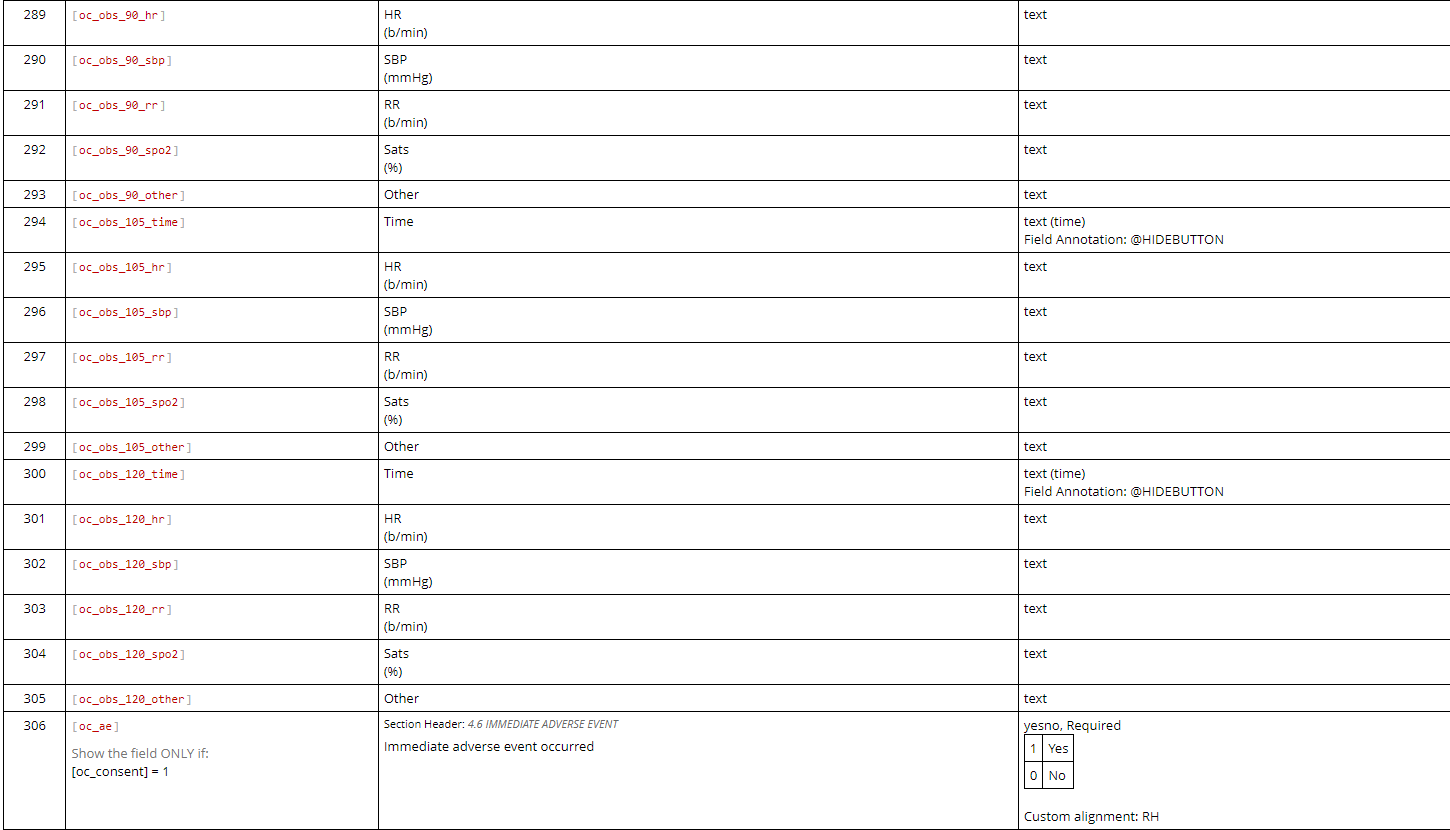


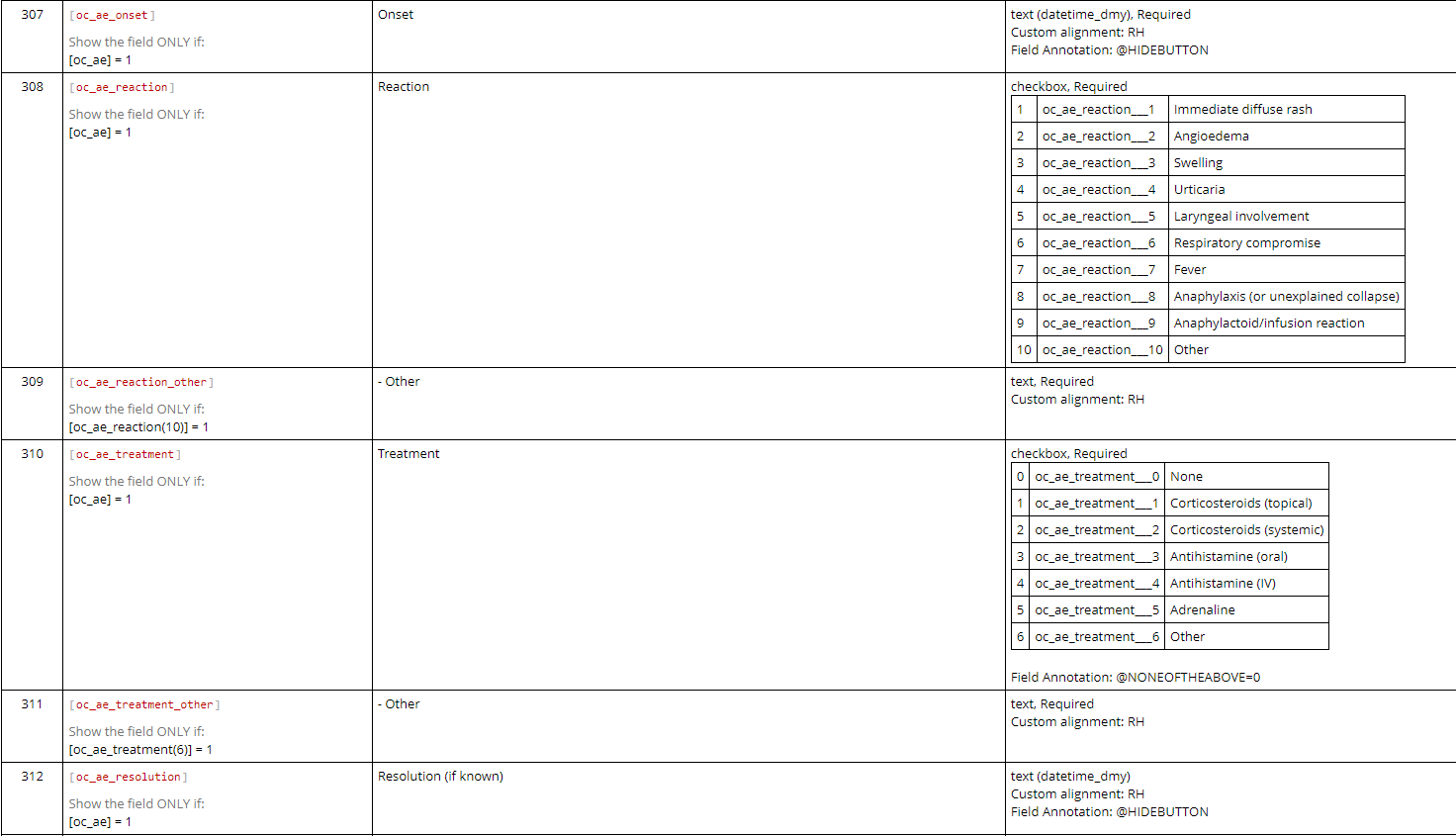


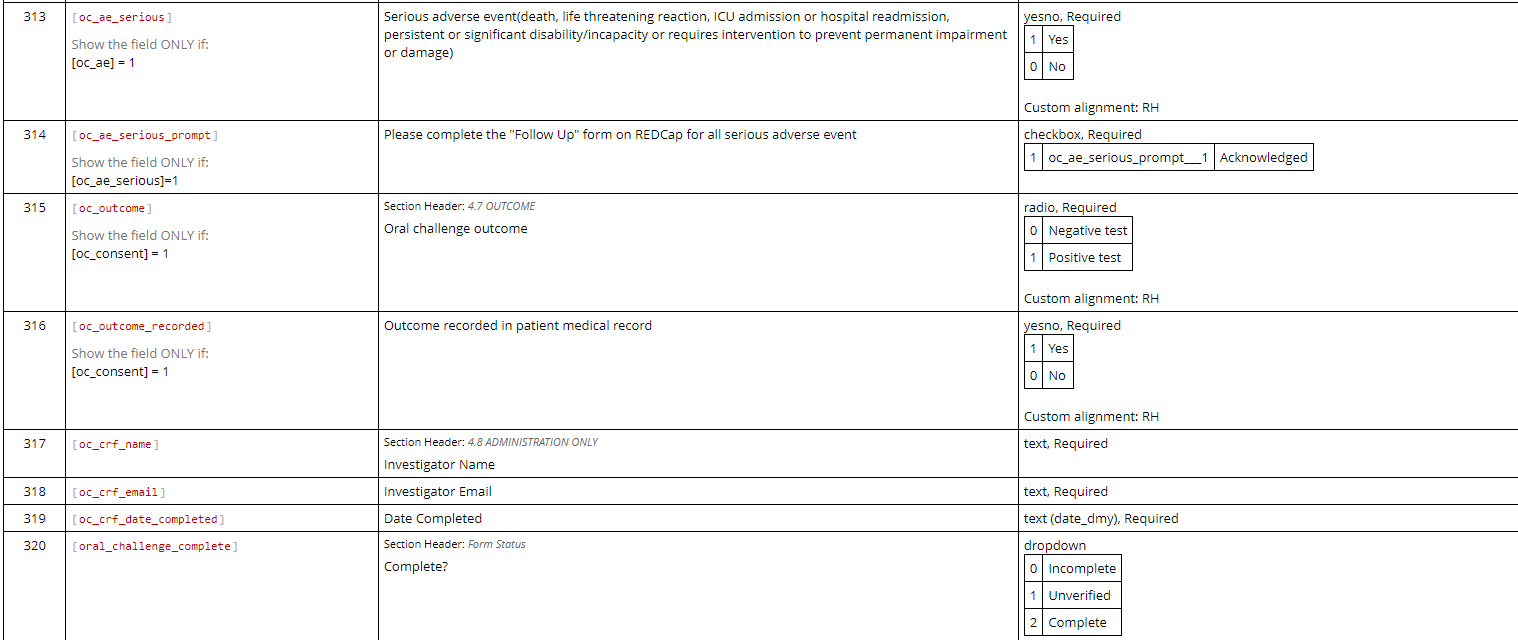


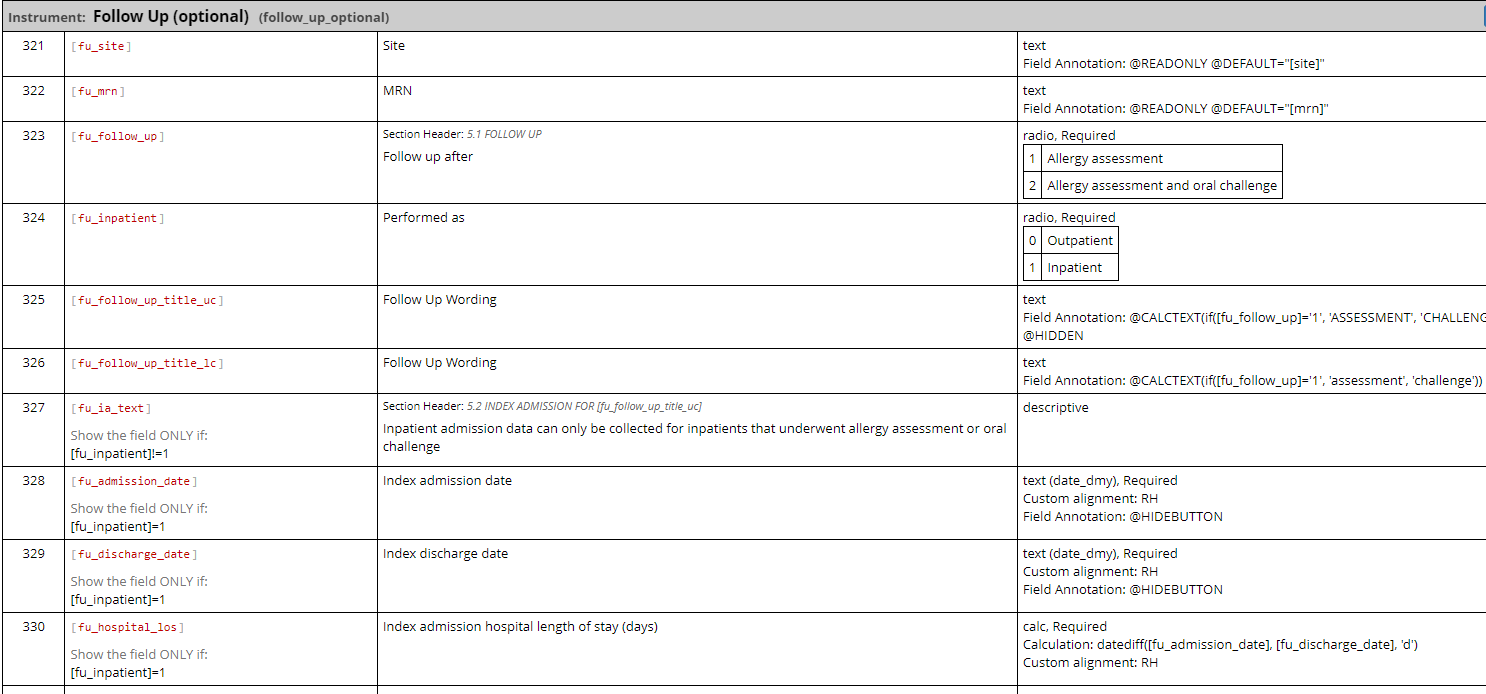


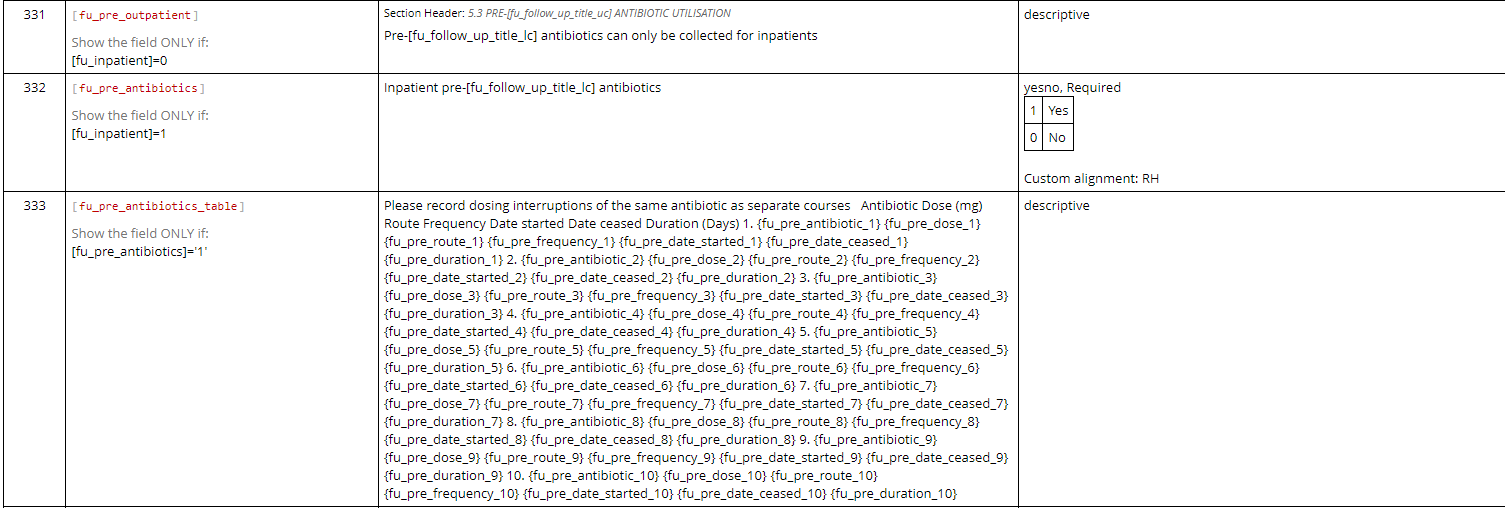


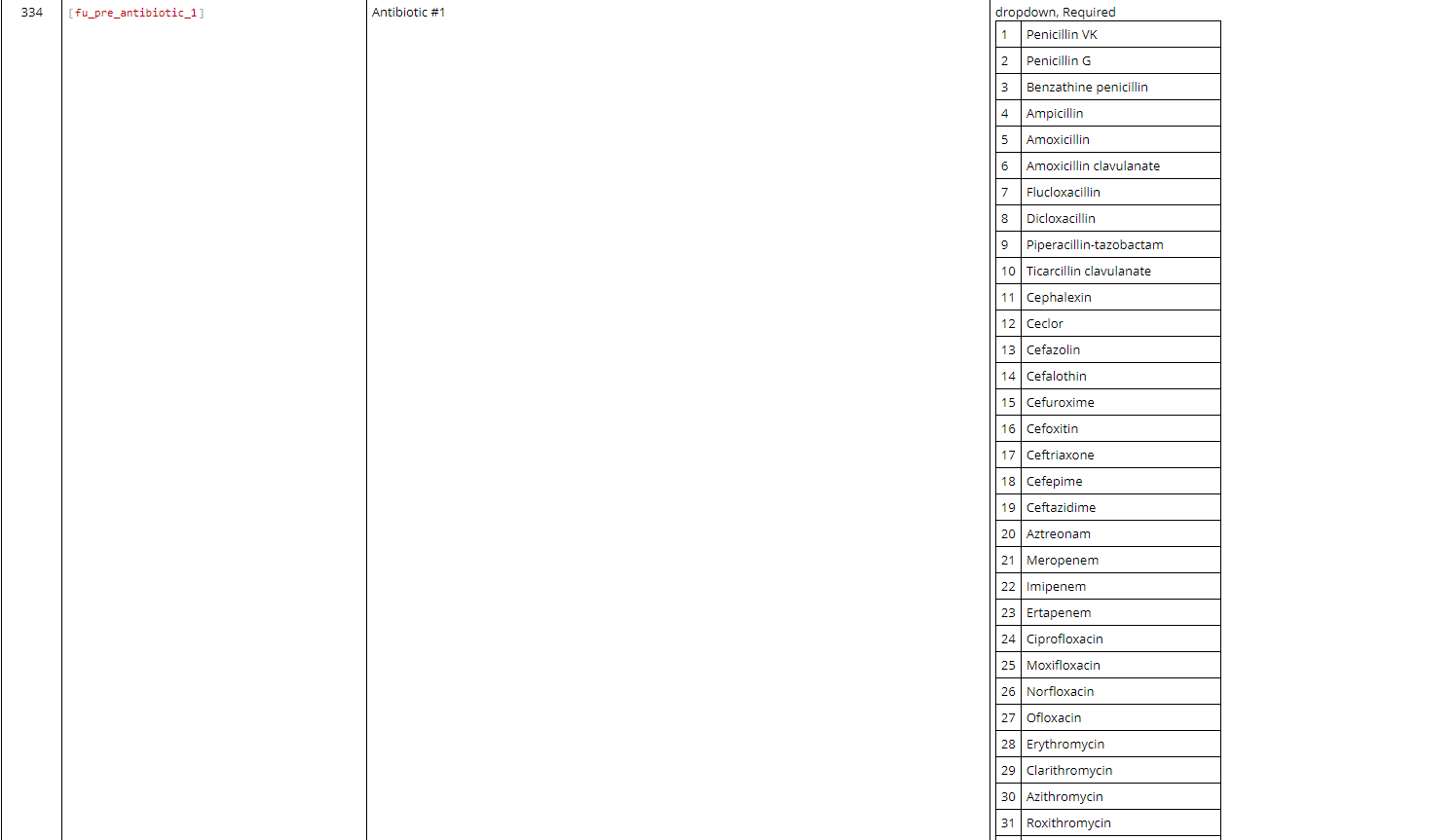


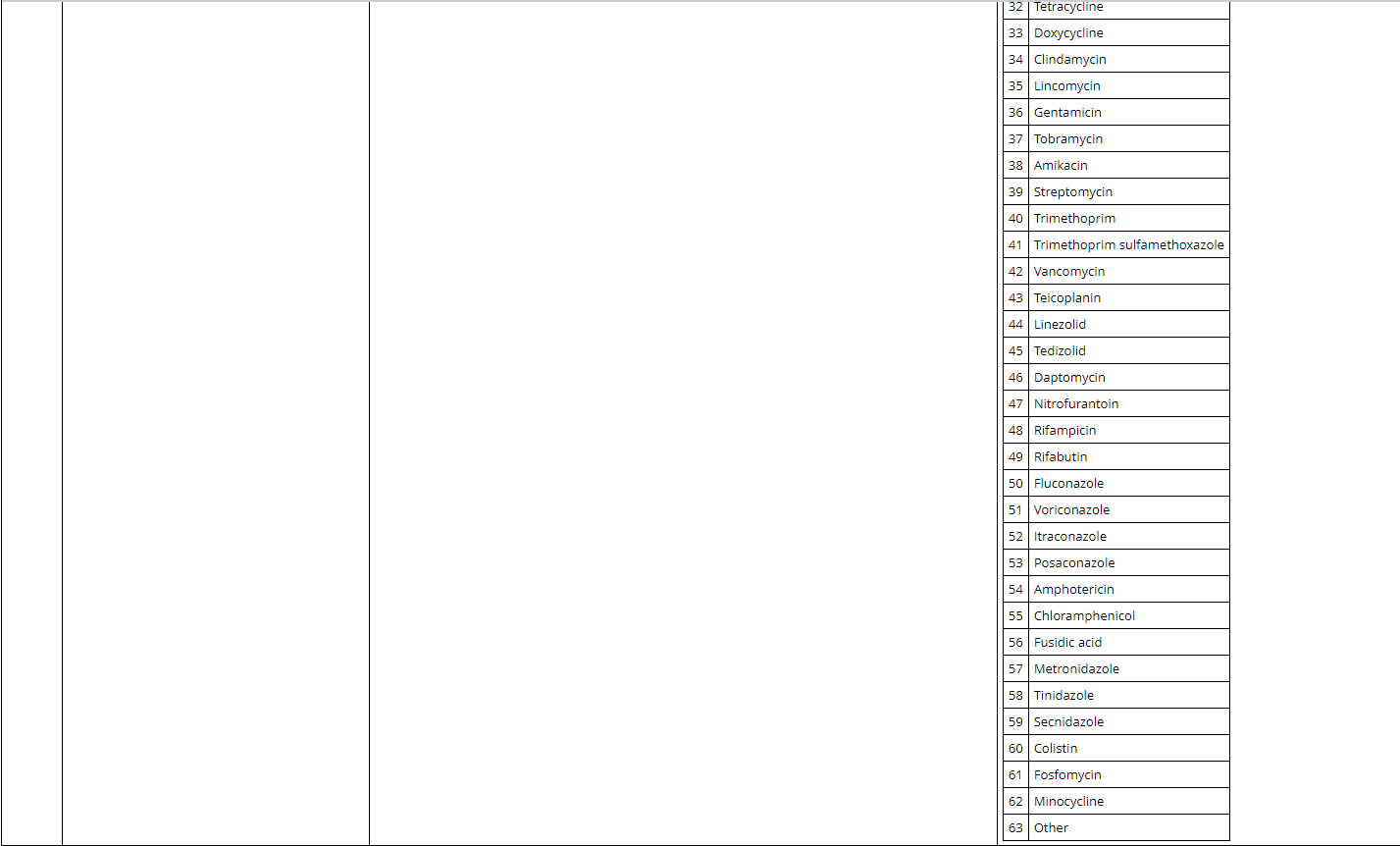


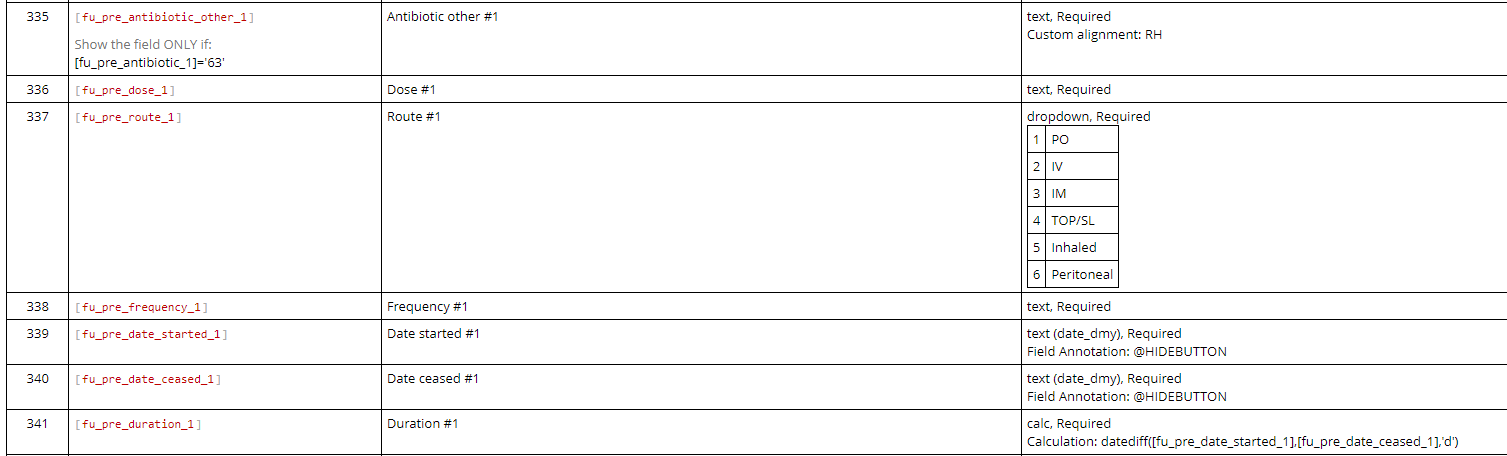


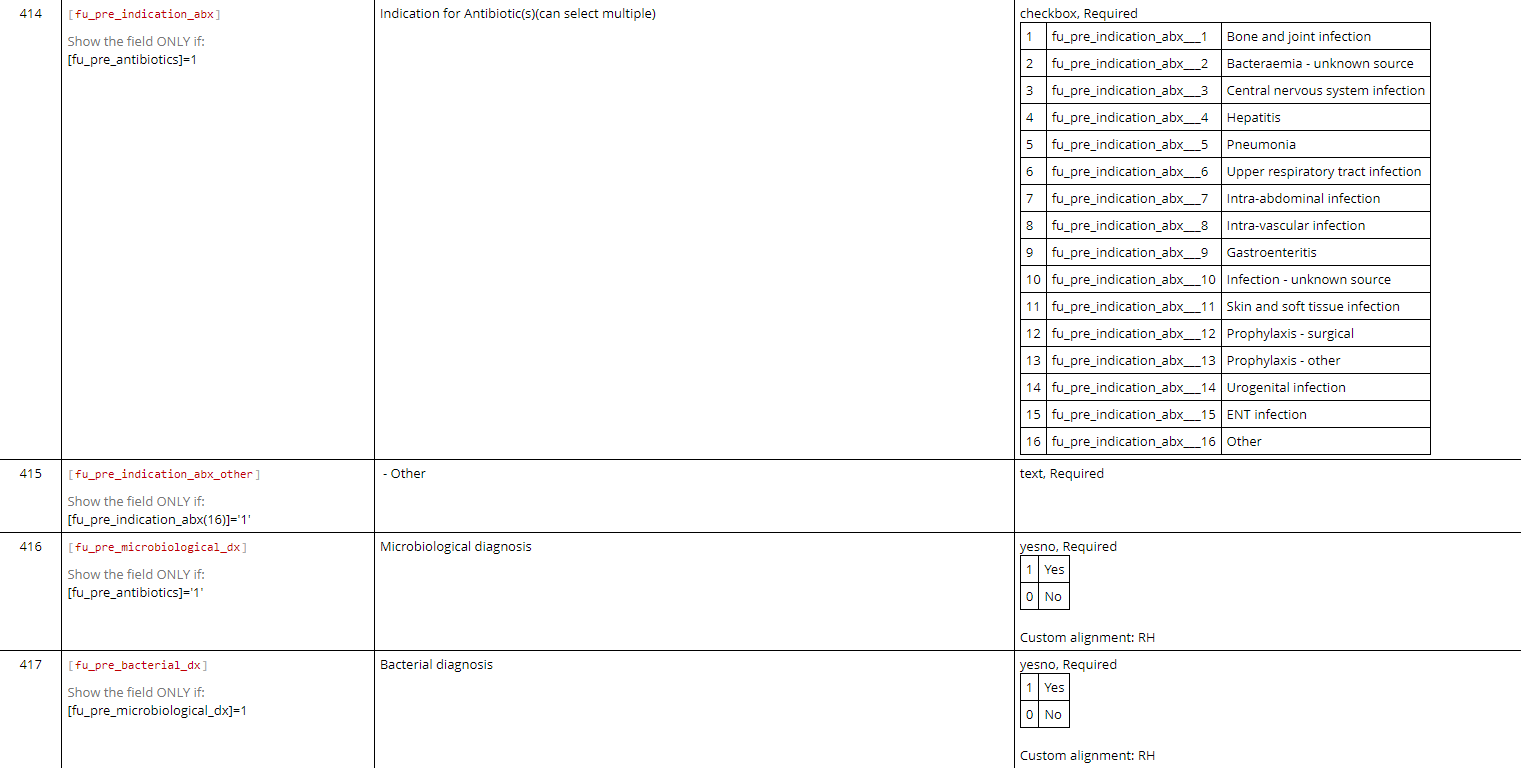


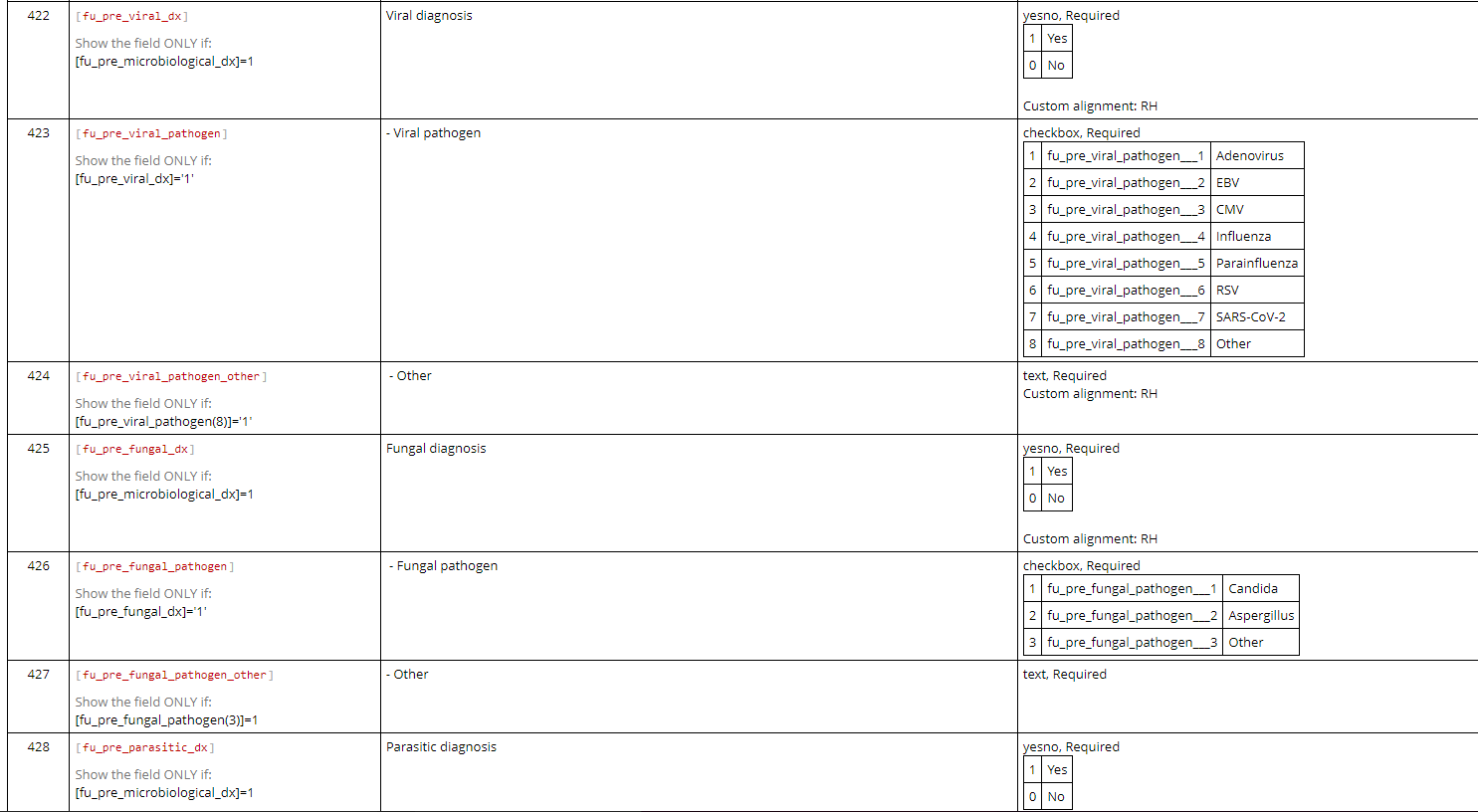

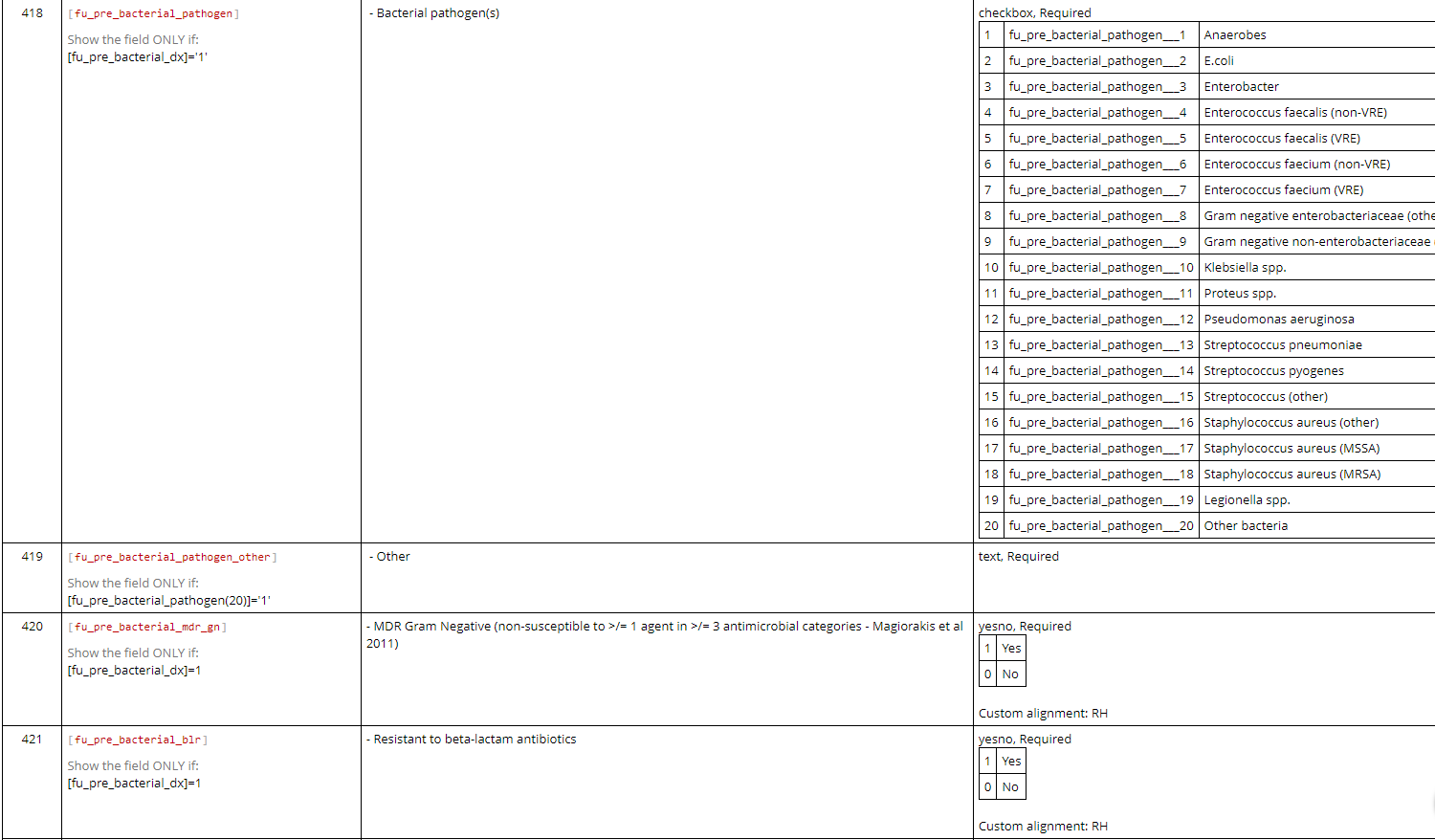


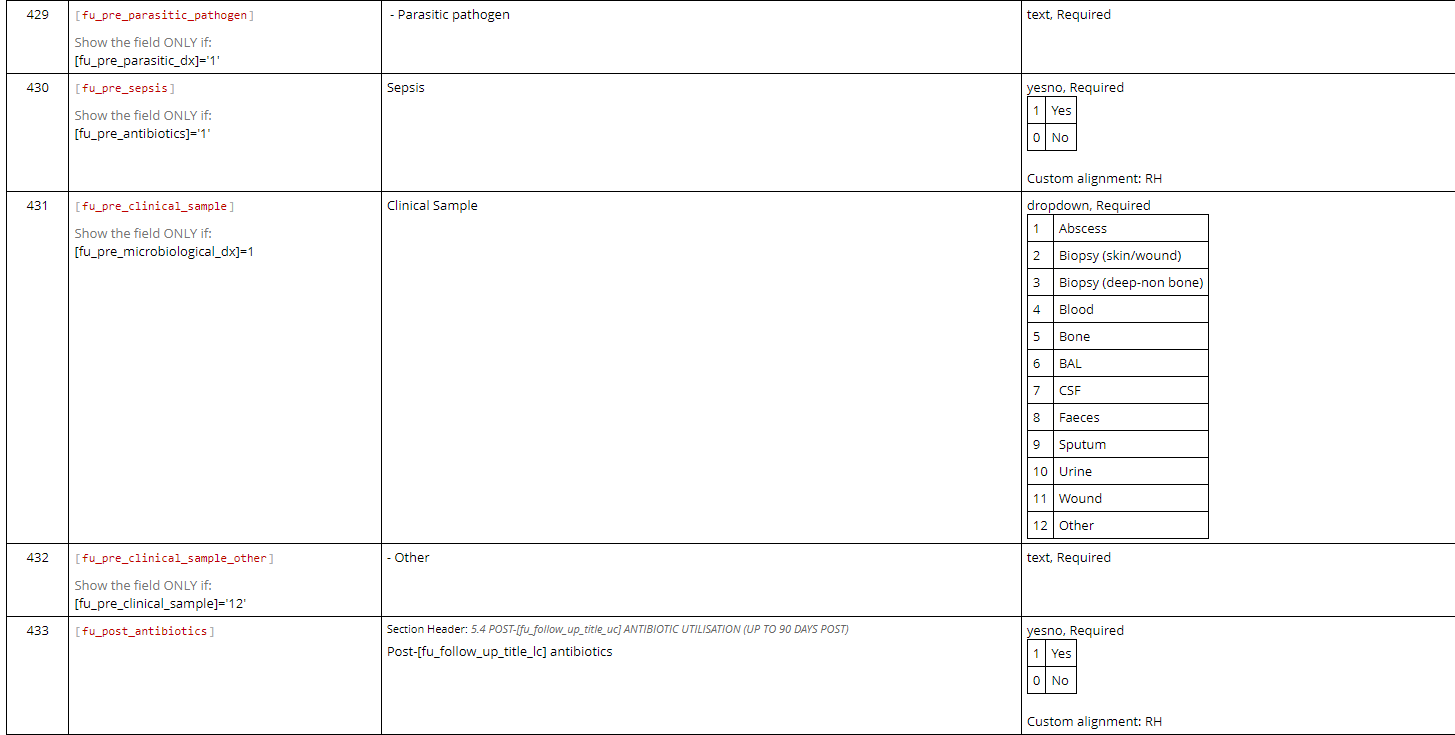


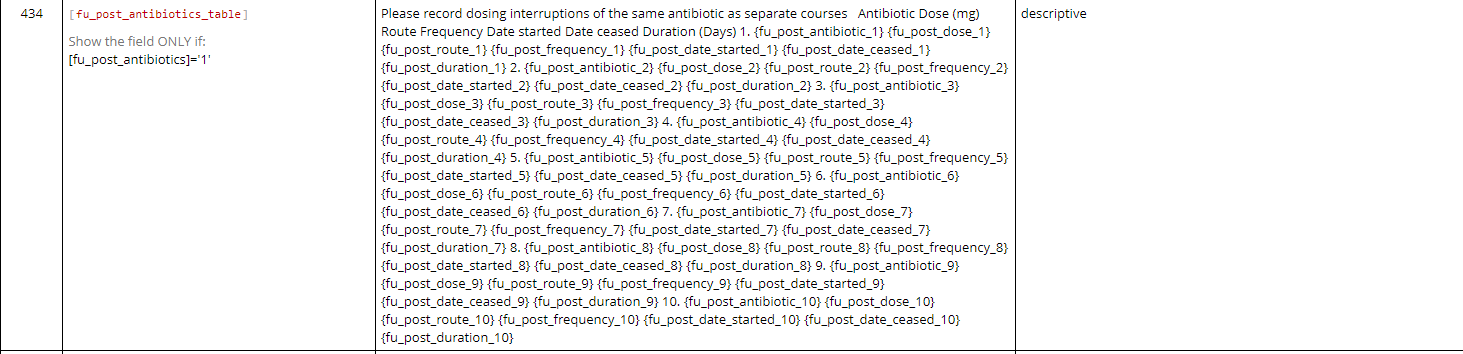


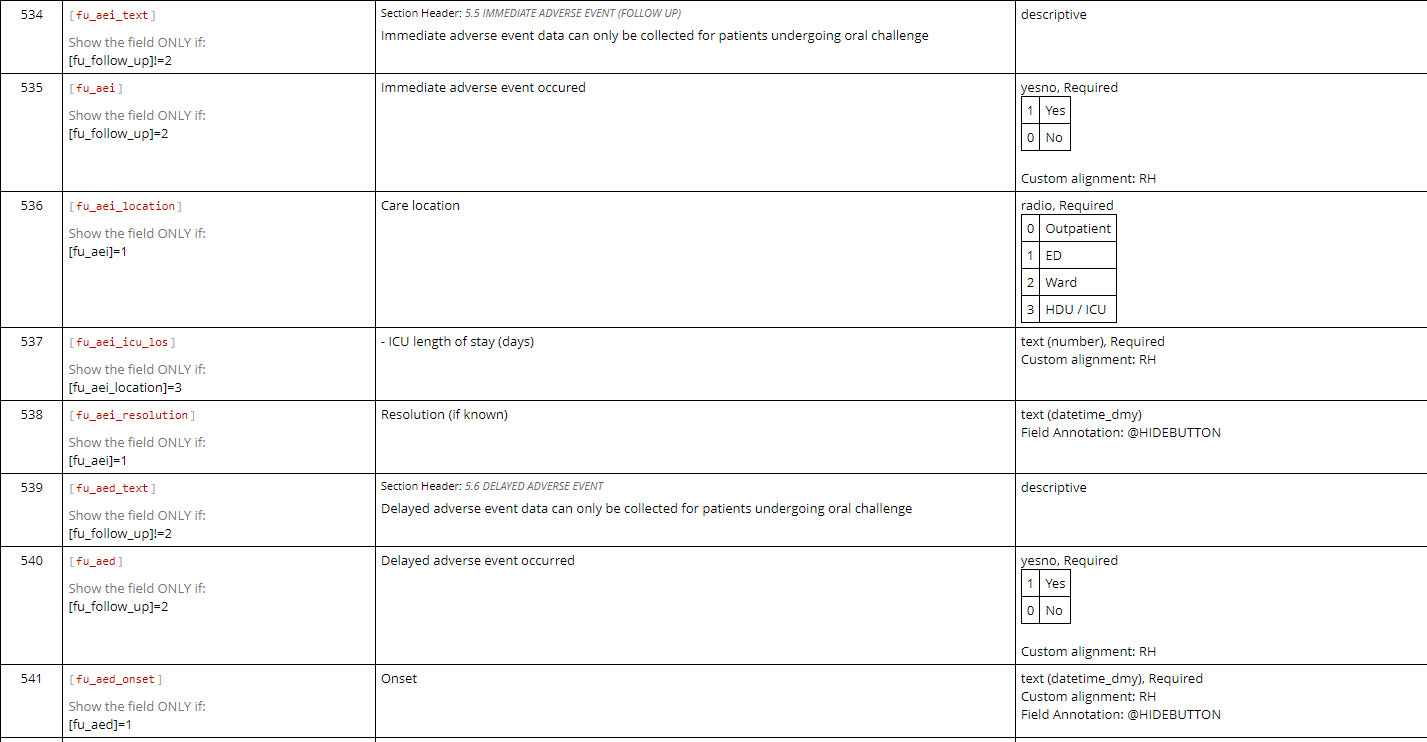


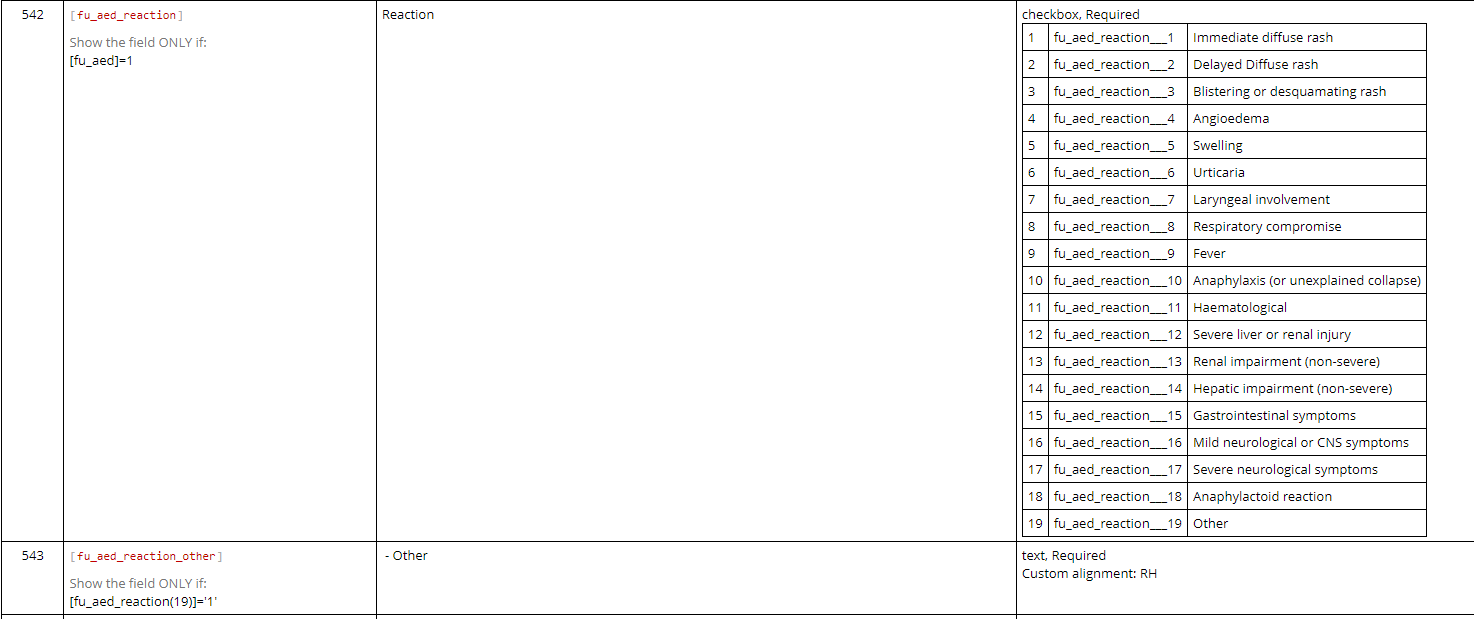


| 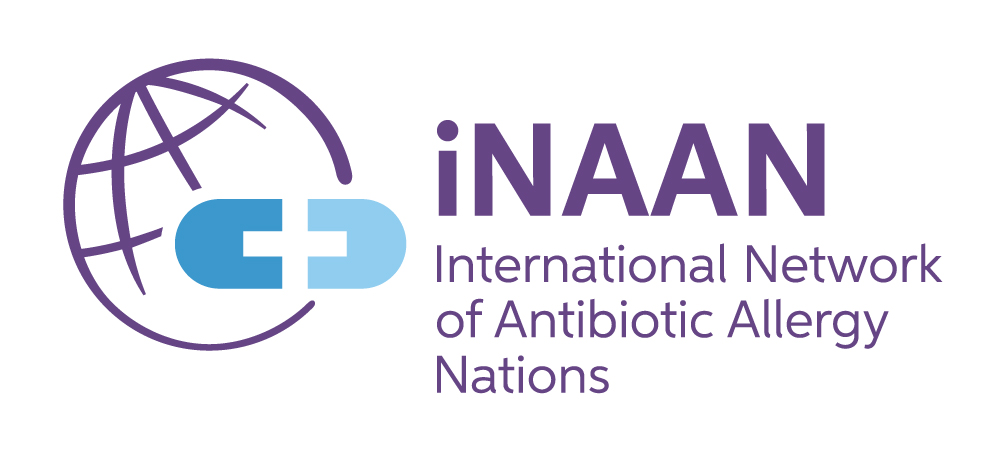 |
| --- |
| International Network of Antibiotic Allergy Nations (iNAAN) |
| Appendix 6: NAAN App and Health Service Report Acceptability Survey |
| Version: 2.0  Date: 26 April 2024 |

***NAAN Smartphone Application***

**Introduction:**

The NAAN app encompasses a penicillin allergy toolkit to enable clinicians to perform a penicillin allergy assessment and record outcomes of direct oral challenge procedures.

A NAAN app acceptability survey, based on the Theoretical Framework of Acceptability (TFA) questionnaire(27), will trigger beginning 6 months post site activation, on a 6-monthly basis, to NAAN app users. The survey is designed to assess opinions and acceptability of the NAAN app with the embedded penicillin allergy toolkit and to enable an avenue to provide feedback.

The survey will be delivered via the NAAN app and data will be collected and stored in the existing iNAAN REDCap database, hosted by BioGrid Australia.

**Survey Questions:**

**Q1.** Do you like or dislike the NAAN App?

**Options:** 1 Strongly dislike / 2 Dislike / 3 No opinion / 4 Like / 5 Strongly like

**Q2.** How much effort does it take to use the NAAN App?

**Options:** 1 No effort at all / 2 A little effort / 3 No opinion / 4 A lot of effort / 5 Huge effort

**Q3.** There are moral or ethics consequences to using/engaging with the NAAN App.

**Options:** 1 Strongly disagree / 2 Disagree / 3 No opinion / 4 Agree / 5 Strongly agree

**Q4.** The NAAN App has improved my ability to perform a point of care penicillin allergy risk assessment and consider oral challenge as a potential delabeling strategy in appropriately assessed patient

**Options:** 1 Strongly disagree / 2 Disagree / 3 No opinion / 4 Agree / 5 Strongly agree

**Q5.** It is clear to me the NAAN App will help improve access to antibiotic allergy assessment and appropriate penicillin allergy delabelling strategies?

**Options:** 1 Strongly disagree / 2 Disagree / 3 No opinion / 4 Agree / 5 Strongly agree

Q6. How confident do you feel about using the NAAN App?

**Options:** 1 Very unconfident / 2 Unconfident / 3 No opinion / 4 Confident / 5 Very confident

**Q7:** The NAAN App interfered with my other priorities.

**Options:** 1 Strongly disagree / 2 Disagree / 3 No opinion / 4 Agree / 5 Strongly agree

**Q8:** How acceptable was the NAAN App to you?

**Options:** 1 Completely unacceptable / 2 Unacceptable / 3 No opinion / 4 Acceptable / 5 Completely Acceptable

Q9. Do you have any feedback or comments about the NAAN App?

**Option:** “freetext” field

***iNAAN Health Services Report***

**Introduction:**

The iNAAN study uses a digitally delivered Audit and Feedback implementation strategy. Feedback is provided via a Health Services Report (HSR) to participating investigators and non-investigator clinicians at each site. An iNAAN HSR acceptability survey, based on the Theoretical Framework of Acceptability (TFA) questionnaire(27), will be delivered electronically to participating iNAAN investigators and non-investigator clinicians with the accompanying HSR. The survey is designed to assess different constructs of acceptability pertaining to the iNAAN HSR and to enable an avenue to provide feedback. The information will inform changes to the HSR.

The online survey will be conducted via Qualtrics software, hosted by the University of Melbourne to meet data management requirements, and takes approximately 5-10 minutes to complete.

**Survey Questions:**

**Q1:** Do you work in an Australian health service?

**Options:** Yes / No

**If yes,**

**Q1a:** Please select your Hospital Peer Group category, in accordance with the Australian Institute of Health and Welfare (AIHW).

**Options:** Principal referral hospitals / Public Acute Group A hospitals / Other acute specialised hospitals / Large Hospitals – regional and remote / Public Acute Group C hospitals / Private Acute Group A hospitals / Children’s hospitals / Other

**Q2:** Please select your role in the iNAAN study at your health service:

**Options:** Investigator (with access to REDCap database and the NAAN App) / Non-investigator clinician (with access to NAAN App)

**Q3:** What is your healthcare discipline?

**Options:** Medical Practitioner (Allergist/Immunologist) / Medical Practitioner (non-Allergist) /

Nurse or Nurse Practitioner / Pharmacist / Other clinician - Specify:

**Q4:** Do you like or dislike the Health Services Report (HSR)?

Options: 1 Strongly dislike / 2 Dislike / 3 No opinion / 4 Like / 5 Strongly like

**Q5:** How much effort does it take to use the HSR?

**Options:** 1 No effort at all / 2 A little effort / 3 No opinion / 4 A lot of effort / 5 Huge effort

**Q6:** There are moral or ethics consequences to using the HSR?

**Options:** 1 Strongly disagree / 2 Disagree / 3 No opinion / 4 Agree / 5 Strongly agree

**Q7:** The HSR has improved my understanding of how to optimise my use of the NAAN App to positively impact patient care at my hospital?

**Options:** 1 Strongly disagree / 2 Disagree / 3 No opinion / 4 Agree / 5 Strongly agree

Q8: It is clear to me how the HSR will help improve access to antibiotic allergy assessment and appropriate penicillin allergy delabelling strategies?

**Options:** 1 Strongly disagree / 2 Disagree / 3 No opinion / 4 Agree / 5 Strongly agree

**Q9:** How confident do you feel about using the information in the HSR?

**Options:** 1 Very unconfident / 2 Unconfident / 3 No opinion / 4 Confident / 5 Very confident

**Q10:** Reading the HSR interfered with my other priorities.

**Options:** 1 Strongly disagree / 2 Disagree / 3 No opinion / 4 Agree / 5 Strongly agree

**Q11:** How acceptable was the HSR to you?

**Options:** 1 Completely unacceptable / 2 Unacceptable / 3 No opinion / 4 Acceptable / 5 Completely Acceptable

**Q12:** Do you have other feedback or comments about the HSR?

**Option:** “freetext field”

| 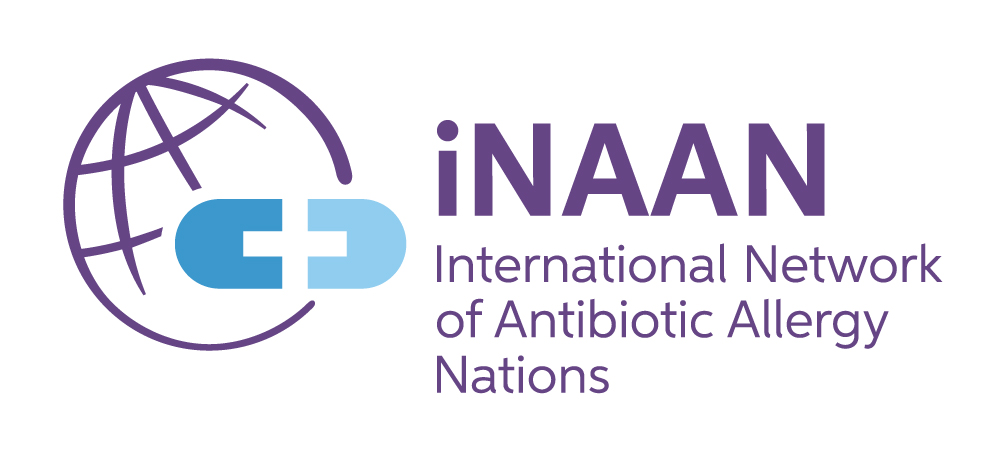 |
| --- |
| International Network of Antibiotic Allergy Nations (iNAAN) |
| Appendix 7: iNAAN Project Request Form |
| Version: 1.0  Date: 22 January 2024 |


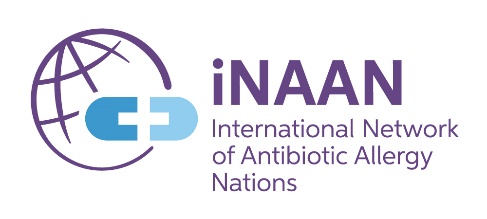


**International Network of Antibiotic Allergy Nations (iNAAN): Project Request Form**

Please return the completed form to: [NAAN@austin.org.au](mailto:NAAN@austin.org.au)

| **Project Title** |  |
| --- | --- |
| **iNAAN Principal Investigator** | [Name] |
|  | [Institution] |
|  | [Department] |
|  | [Email] |
|  | [Phone number] |
| **Other Investigators or people who will have access to the data *(expand the list as required)*** | [Name] |
|  | [Institution] |
|  | [Department] |
|  | [Email] |
|  | [Phone number] |
| **Brief explanation of why the iNAAN database is important for your project** |  |
| **Project Plan** | [Hypothesis] |
|  | [Objectives] |
|  | [Study design] |
|  | [Sample size and Population Characteristics] |
|  | [Description of plan to translate research findings into clinical care, education, and training] |
| **Specific data required**  **(REDCap instruments +/- data fields)** |  |
| **Security and archiving** | [Description of the security and archiving arrangements for data] |
| **Project timeline** |  |
| **Proposed method of publication / presentation of results** |  |
| **Authorship Structure** |  |


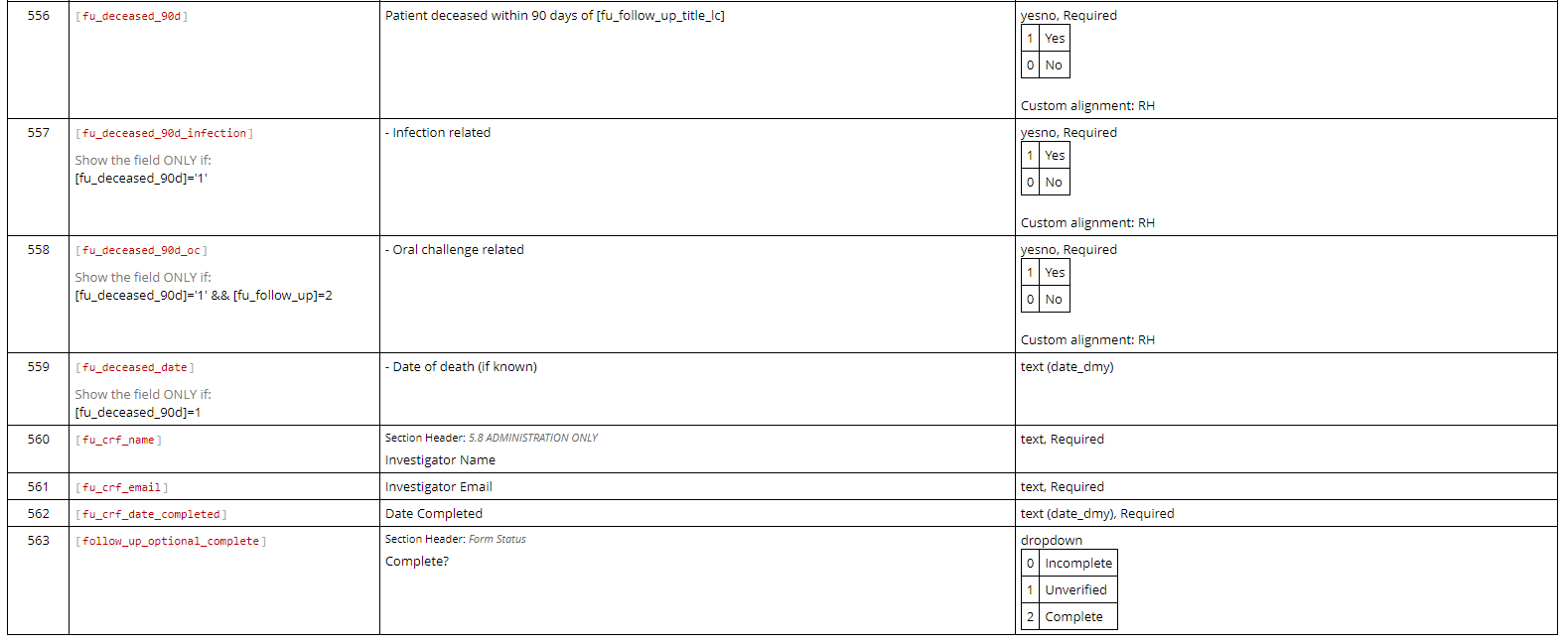

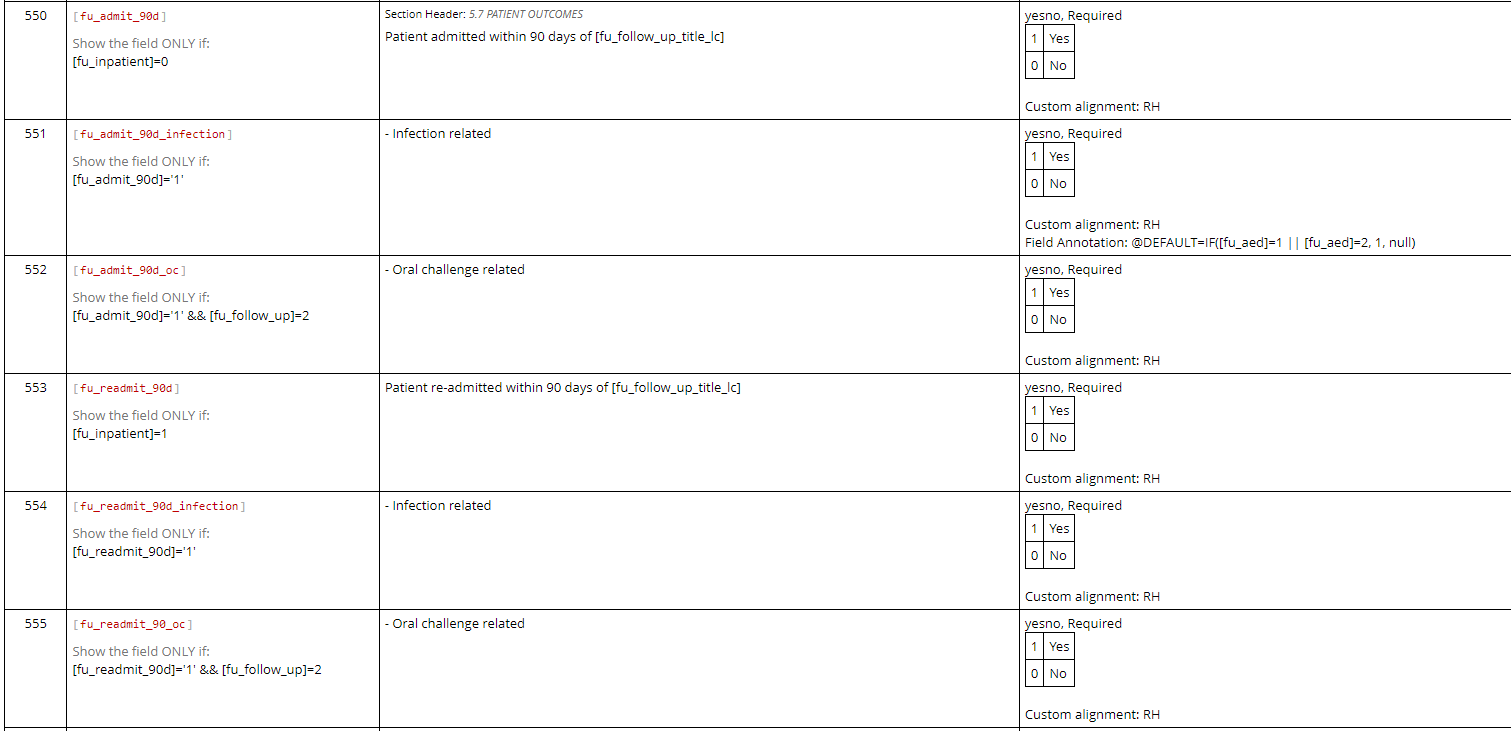

Supplement: S1 File — (DOCX) [file pone.0330724.s009.docx]
